# Examining the relationship between secondhand smoke and non-malignant digestive system diseases: Mendelian randomization evidence

**DOI:** 10.18332/tid/200338

**Published:** 2025-02-14

**Authors:** Yujun Yu, Yongyun Jin

**Affiliations:** 1 Department of Colorectal Surgery, Hangzhou Red Cross Hospital, Hangzhou, China

**Keywords:** secondhand smoke, non-malignant digestive system diseases, ulcerative colitis, Mendelian randomization

## Abstract

**INTRODUCTION:**

Secondhand smoke (SHS) may exacerbate the global disease burden, particularly in workplace settings. Observational studies have implicated SHS as a risk factor for various non-malignant digestive system diseases (NMDSD), yet establishing a causal relationship remains challenging. Therefore, we conducted a Mendelian randomization (MR) study to explore whether workplace exposure to SHS is associated with NMDSD.

**METHODS:**

This study utilized a secondary dataset analysis based on Genome-Wide association study (GWAS) summary data. Genetic variants associated with exposure to SHS in the workplace were used as instrumental variables. Genome-wide association study (GWAS) summary data for SHS were obtained from the UK Biobank. GWAS summary data for NMDSD were sourced from the FinnGen study, the International Inflammatory Bowel Disease Genetics Consortium (IIBDGC), and a large-scale study conducted in Japan. We employed inverse variance-weighted (IVW), MR-Egger, and weighted median methods for MR analysis. Additionally, sensitivity analyses were conducted to ensure the robustness of our findings.

**RESULTS:**

According to the IVW model, SHS in the workplace was positively associated with ulcerative colitis (UC) (OR=2.03; 95% CI: 1.03–4.05; p=0.04). There was no evidence of horizontal pleiotropy biasing causality (p>0.05), and leave-one-out analysis confirmed the stability and robustness of this association.

**CONCLUSIONS:**

Our study identifies an association between regular exposure to SHS in the workplace and an increased risk of ulcerative colitis. However, the potential influence of active smoking or exposure to SHS from other sources cannot be excluded. Further research is needed to confirm these findings.

## INTRODUCTION

Non-malignant digestive system diseases (NMDSD) impose substantial healthcare utilization and expenditures, constituting a significant medical and economic burden^1^. Exposure to secondhand smoke (SHS), also known as passive or involuntary smoking, is a major public health issue associated with tobacco, contributing significantly to the global disease burden. Despite a gradual decline in smoking rates over the past half-century^2^, an assessment of passive smoking exposure in the American workforce revealed that nearly one-fifth of non-smoking employees are exposed to SHS at work, with over half encountering SHS at least twice weekly^3^. It is estimated that passive smoking contributes to hundreds of thousands of deaths annually^4^. Numerous epidemiological studies indicate associations between tobacco exposure and various NMDSD, including gastroesophageal reflux disease (GERD)^5^, irritable bowel syndrome (IBS)^6^, pancreatitis^7,8^, and inflammatory bowel disease (IBD)^9-12^. Evidence linking SHS to other NMDSD risks is limited and inconsistent. However, whether these associations are causal remains uncertain, as most evidence is derived from observational studies susceptible to bias from reverse causation and confounding. Establishing a relationship between SHS in the workplace and NMDSD is crucial, as it may provide valuable insights for future public policies and clinical interventions.

Mendelian randomization (MR) is an epidemiological analysis method that uses single nucleotide polymorphisms (SNPs) as instrumental variables (IVs) to assess causal relationships between exposure and outcomes^13^. This method avoids confounding factors such as environmental exposures and reduces the impact of reverse causation, thereby enhancing the persuasiveness of results^14^. In this study, we conducted a Mendelian randomization (MR) study to further investigate the causal relationship between workplace exposure to SHS and NMDSD.

## METHODS

**Figure 1:**
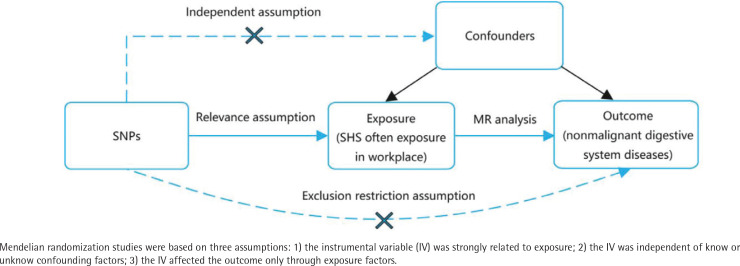
Overview of the design

The design of the two-sample Mendelian randomization (MR) study is illustrated in Figure 1. In this study, SHS in the workplace was regarded as exposure data and NMDSD was regarded as outcome data. SNPs were selected as IVs for further analyses. MR analyses must fulfill the following three assumptions: 1) Genetic variants should be significantly associated with the exposure; 2) Genetic variants should be associated with the exposure but not related to any confounding factors associated with the outcome; and 3) Genetic variants should not influence the outcome through pathways related to the exposure or confounding factors. Failure to meet any of these assumptions can make causal inference challenging^15^. It is important to note that the data used in this study are publicly available and free of charge, so there is no need to provide further ethical review and informed consent.

### Data sources and instrumental variable selection

We extracted independent SHS-related SNPs from the second round of GWAS results from the UKB (http://www.nealelab.is/uk-biobank): workplace had a lot of cigarette smoke from other people smoking (self-reported: often), sex (male, female), cases (n=14941), controls (n=74862), which has been previously utilized in the study by Wang et al.^16^. We used p<5.0×10^-5^ as the genome-wide significance level to select genetic variants associated with ‘workplace had a lot of cigarette smoke from other people smoking’. To ensure a minimum of 20 residual SNPs after clumping for independence and harmonizing with outcome data, the threshold was lowered from p<5.0×10^-8^ to p<5.0×10^-5^ to include a sufficient number of SNPs. Subsequently, linkage disequilibrium tests were performed on these SNPs to ensure their independence (r^2^ <0.001; kb >10000). Finally, we searched all 106 SNPs associated with SHS in the workplace in the LDtrait Tool (https://ldlink.nih.gov/?tab=ldtrait) to assess whether any of these variants were associated to potential confounders or directly influenced the outcome (p<5×10^-8^). No SNPs were excluded during this process. In addition, we calculated the F-statistic for instrumental variables to mitigate bias caused by weak instruments, from F=R^2^(N-2)/(1-R^2^), with the condition that F>10. SNPs significantly associated with SHS are shown in Supplementary file Table 1. The GWAS summary statistics for the outcome data in our study included eight diseases: GERD, IBS, cholelithiasis, acute pancreatitis, chronic pancreatitis, IBD, ulcerative colitis (UC), and Crohn’s disease (CD). Summary data for GWAS on non-malignant digestive system diseases were sourced from the FinnGen study (https://www.finngen.fi/en), the International Inflammatory Bowel Disease Genetics Consortium (IIBDGC)^17^, and a large-scale study conducted in Japan by Sakaue et al.^18^. Characteristics of exposure and outcome GWAS samples are detailed in Table 1.

**Table 1:** Detailed information of the genome-wide association study (GWAS) used in this study, involving a European population

*Dataset*	*Exposure/Outcome*	*Year*	*Sample size* *(cases/controls)*
ukb-d-22611_2	Workplace had a lot of cigarette smoke from other people smoking: often	2018	14941/74862
ebi-a-GCST90018848	GERD	2021	32957/434296
finn-b-K11_IBS	IBS	2021	4605/182423
finn-b-K11_CHOLELITH	Cholelithiasis	2021	19023/195144
finn-b-K11_ACUTPANC	Acute pancreatitis	2021	3022/195144
finn-b-K11_CHRONPANC	Chronic pancreatitis	2021	1737/195144
ieu-a-31	IBD	2015	12882/21770
ieu-a-32	UC	2015	6968/20464
ieu-a-30	CD	2015	5956/14927

GERD: gastroesophageal reflux disease. IBS: irritable bowel syndrome. IBD: inflammatory bowel disease. UC: ulcerative colitis. CD: Crohn’s disease.

### Statistical analysis

In this study, MR analysis was conducted using the TwoSampleMR package in R software. We employed three analysis methods: MR Egger, weighted median, and inverse variance-weighted (IVW), to assess the causal relationship between SHS and NMDSD. Specifically, IVW was used as the primary MR analysis method, employing weighted regression of SNP-specific Wald ratios to evaluate the causal effect of exposure on the outcome^19^. MR Egger and weighted median were used as supplementary analyses to test the robustness of results: 1) Weighted median^20^, this method provides consistent causal effect estimates even when up to 50% of the IVs are invalid; and 2) MR Egger^21^, this method assesses pleiotropic effects of genetic variants on the outcome and provides consistent causal effect estimates under weaker assumptions, though it may increase Type I error rates. By combining these methods, we robustly assessed the causal impact of SHS on NMDSD. Given that the outcome was binary, the effect estimates were presented as odds ratios (ORs) along with their corresponding 95% confidence intervals (CIs).

### Sensitivity analysis

This study used several sensitivity analyses to assess the robustness of the results. Cochran’s Q test^22^ was used to assess heterogeneity among individual SNPs. If the p>0.05, it indicates no heterogeneity, and the fixed-effects inverse variance-weighted (IVW) method is employed. If the p<0.05, a random-effects IVW model is used. The MR Egger^14^ method was used to detect horizontal pleiotropy. The intercept of MR Egger regression indicates the pleiotropic effects of genetic variants on the outcome and provides consistent causal effect estimates under weaker assumptions. A p>0.05 suggests no horizontal pleiotropy, indicating no confounding in the study. Finally, a leave-one-out analysis was conducted to determine if the MR results were significantly influenced by any single SNP.

## RESULTS

### MR analysis of SHS in the workplace and non-malignant digestive system diseases

**Figure 2:**
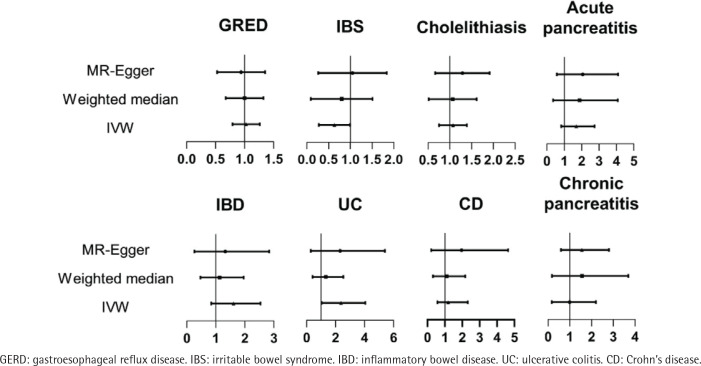
Forest plot of OR for secondhand smoking on nonmalignant digestive system diseases

In this study, the overall F-value was 19.16. The study demonstrates a positive association between SHS in the workplace and UC (OR=2.03; 95% CI: 1.03–4.05; p=0.04). However, there was no causal relationship found between SHS in the workplace and GERD (OR=1.01; 95% CI: 0.80–1.27; p=0.94), IBS (OR=0.62; 95% CI: 0.38–1.00; p=0.05), cholelithiasis (OR=1.04; 95% CI: 0.77–1.41; p=0.80), acute pancreatitis (OR=1.51; 95% CI: 0.83–2.74; p=0.18), chronic pancreatitis (OR=1.28; 95% CI: 0.59–2.80; p=0.54), IBD (OR=1.45; 95% CI: 0.84–2.54; p=0.18), and CD (OR=1.15; 95% CI: 0.57–2.32; p=0.69) (Table 2, Figure 2).

**Table 2:** MR results of secondhand smoking on non-malignant digestive system diseases

*Diseases*	*IVW*			*MR-Egger*			*Weighted median*		
	*OR*	*95% CI*	*p*	*OR*	*95% CI*	*p*	*OR*	*95% CI*	*p*
GERD	1.01	0.80–1.27	0.94	0.88	0.56–1.38	0.58	0.97	0.69–1.34	0.84
IBS	0.62	0.38–1.00	0.05	0.85	0.38–1.92	0.70	0.59	0.22–1.59	0.30
Cholelithiasis	1.04	0.77–1.41	0.80	1.19	0.72–1.96	0.51	0.97	0.57–1.66	0.91
Acute pancreatitis	1.51	0.83–2.74	0.18	1.52	0.57–4.10	0.41	1.21	0.36–4.08	0.76
Chronic pancreatitis	1.28	0.59–2.80	0.54	0.60	0.17–2.20	0.45	0.82	0.18–3.69	0.80
IBD	1.45	0.84–2.54	0.18	0.87	0.26–2.84	0.81	0.96	0.47–1.96	0.90
UC	2.03	1.03–4.05	0.04	1.25	0.29–5.40	0.77	1.02	0.41–2.54	0.97
CD	1.15	0.57–2.32	0.69	1.02	0.22–4.64	0.98	0.84	0.32–2.17	0.72

IVW: inverse variance weighted. GERD: gastroesophageal reflux disease. IBS: irritable bowel syndrome. IBD: inflammatory bowel disease. UC: ulcerative colitis. CD: Crohn’s disease.

### Sensitivity analysis of MR

Firstly, in the heterogeneity test, the p-value of Cochran’s Q test was <0.05, indicating heterogeneity among SNPs (Table 3). Therefore, in this MR analysis, the random-effects IVW method was employed as the primary analysis approach. The MR Egger regression intercept indicated no horizontal pleiotropy concerning the instrumental variables for SHS. Additionally, the leave-one-out analysis demonstrated that the potential causal relationship between SHS in the workplace and NMDSD in the European population was not driven by any single SNP (Supplementary file Figure 3). Furthermore, the funnel plot provided a visual representation of heterogeneity (Supplementary file Figure 4). A symmetrical funnel shape typically suggests the absence of publication bias, whereas any asymmetry may indicate heterogeneity or bias. In this study, the funnel plot showed that the SNPs were symmetrical (Supplementary file Figure 4).

**Table 3:** The results of pleiotropy and heterogeneity tests

*Outcome*	*Pleiotropy* *p*	*Heterogeneity* *p*
GERD	0.49	0.08
IBS	0.33	0.54
Cholelithiasis	0.53	0.06
Acute pancreatitis	0.99	0.62
Chronic pancreatitis	0.16	0.87
IBD	0.33	0.01
UC	0.46	0.01
CD	0.86	0.09

ERD: gastroesophageal reflux disease. IBS: irritable bowel syndrome. IBD: inflammatory bowel disease. UC: ulcerative colitis. CD: Crohn’s disease.

## DISCUSSION

In this study, two-sample MR analysis indicated a relationship between SHS in the workplace and an increased risk of UC. However, no causal relationship was found between SHS and GERD, IBS, cholelithiasis, acute pancreatitis, chronic pancreatitis, and CD.

Previous MR studies have primarily focused on the health impacts of smoking on smokers^23^. However, globally, 40% of children, 33% of male non-smokers, and 35% of female non-smokers are exposed to SHS, with passive smoking causing hundreds of thousands of deaths annually^4^. Therefore, the health issues of passive smokers require more attention, which is the aim and motivation of our study. In recent years, numerous observational studies have indicated that tobacco exposure is a risk factor for various diseases, including GRED^24^, IBS^24^, cholelithiasis^25^, and pancreatitis^26,27^. However, some studies have reached different conclusions. A cross-sectional study found a significantly lower prevalence of IBS among smokers^6^. Some researchers did not find an association between tobacco exposure and cholelithiasis^28^. Traditional observational studies have methodological limitations, such as being influenced by confounding factors and reverse causation, which can be circumvented by MR studies.

This MR study confirms the causal relationship between SHS in the workplace and UC. Tobacco exposure has been shown to increase the production of many pro-inflammatory cytokines and decrease the levels of anti-inflammatory cytokines^29^, alter microcirculation, and significantly reduce blood flow to the gastrointestinal mucosa^30^, which may favor the development of inflammatory diseases. Additionally, tobacco exposure has significant effects on epigenetic modifications and transcriptional regulation^31^, which may lead to immune system diseases. Although UC and CD share overlapping mechanisms of pathogenesis, many studies indicate that they differ in genetics^32^, pathogenesis^33^, cellular immunity^34^, and response to probiotic therapy^35^, which may explain the lack of a causal relationship between SHS and CD.

In this study, the IVW method was used as the primary MR analysis approach to assess the causal relationship between SHS in the workplace and UC. Although the IVW method is widely utilized in many MR studies and typically provides robust causal effect estimates^16,19^, in our analysis, it revealed a significant but marginal effect, particularly when the lower bound of the effect approached the null effect value of 1. This marginal effect suggests that the estimated causal relationship may be weak, possibly due to factors such as sample size, the selection of genetic instruments, or other potential sources of bias. Therefore, cautious interpretation is warranted, particularly concerning the robustness of the causal inference and its clinical relevance. Future studies should aim to validate these preliminary findings by utilizing larger MR studies or more advanced statistical methodologies.

### Strengths and limitations

Our study has several advantages. First, we used MR to evaluate the association between SHS and NMDSD, which is less susceptible to confounding factors and reverse causality compared to observational studies. Second, our exposure IVs were derived from large-scale GWAS, providing strong and reliable genome-wide association SNP correlations, thus avoiding biases caused by weak instruments. Additionally, we conducted sensitivity analyses to further confirm the reliability of this study.

However, our study has some limitations. First, we used p<5×10^-5^ as the threshold for genome-wide significance to select variants associated with exposure, which reduces the specificity of SNPs. Second, due to the ethnic limitations of this study, the results cannot be generalized to other ethnicities. Third, we did not stratify the causal relationship between SHS and potential diseases by gender and subtype, although some studies suggest that this may affect the causal relationship. Moreover, a major limitation of our study is the potential for residual confounding from unmeasured factors that may influence both SHS exposure and disease development, which could introduce bias into the results. At the same time, unobserved pleiotropy is another potential issue that may affect the robustness of causal inference. Furthermore, another significant limitation is that SHS exposure was defined using self-reported data, which may introduce information bias or measurement errors. Lastly, it is important to note that the association between SHS in the workplace and NMDSD was assessed using a dichotomous exposure model, without considering a dose-response relationship. This limitation should be considered when interpreting our findings. Future studies that include dose-response data may offer more comprehensive insights into the relationship between SHS in the workplace and NMDSD. In addition, a major limitation of this study is the failure to account for active smoking or exposure to SHS from other sources, such as the home environment, which could also contribute to increased exposure levels. This factor should be addressed in future research to provide a more comprehensive understanding of the relationship between SHS exposure and health outcomes.

## CONCLUSIONS

Our MR study identifies an association between regular exposure to SHS in the workplace and an increased risk of UC. These findings underscore the importance of ongoing efforts to implement and reinforce smoke-free regulations, especially in workplace environments. Consistent evidence indicates that smoke-free laws can reduce SHS exposure in workplaces^36^, thereby reducing the burden of disease. However, the current analysis was unable to account for exposure to SHS in other settings or for active smoking status, which may limit the interpretation of these findings. Further research is needed to confirm these findings.

## Supplementary Material



## Data Availability

The data from the UK Biobank can be obtained from http://www.nealelab.is/uk-biobank. The data from the FinnGen study can be obtained from https://www.finngen.fi/en. Other data are available in original cited work.
