# Supplementary material for: Examining the relationship between secondhand smoke and non-malignant digestive system diseases: Mendelian randomization evidence
Source: Tob Induc Dis. 2025 Feb 14;23:10.18332/tid/200338. doi: 10.18332/tid/200338 (PMC11826309; doi:10.18332/tid/200338)
Supplement: Supplementary file 1 [file TID-23-16-s1.pdf]

SUPPLEMENTARY TABLES

Table S1. The SNPs Significantly Associated with SHS in the Workplace
Table S2. Detail Information of the Genetic Variants in the MR Analysis of SHS and GRED
Table S3. Detail Information of the Genetic Variants in the MR Analysis of SHS and IBS
Table S4. Detail Information of the Genetic Variants in the MR Analysis of SHS and Cholelithiasis
Table S5. Detail Information of the Genetic Variants in the MR Analysis of SHS and Acute pancreatitis
Table S6. Detail Information of the Genetic Variants in the MR Analysis of SHS and Chronic pancreatitis
Table S7. Detail Information of the Genetic Variants in the MR Analysis of SHS and IBD
Table S8. Detail Information of the Genetic Variants in the MR Analysis of SHS and UC
Table S9. Detail Information of the Genetic Variants in the MR Analysis of SHS and CD

Table S1. The SNPs Significantly Associated with SHS in the Workplace

SNP	chr.expo	pos.exposure	effect_a	other_al	eaf.exposur	beta.exposur	se.exposure	pval.exposur	samplesi	id.expos	exposure	mr_keep.	pval_ori	data_source.exposure
s55491249	1	226735908	C	G	0.103226	-0.0123639	0.00294957	2.77E-05	89714	b-d-2261	1l:ukb-d-2	TRUE	reported	igd
s7267912	1	66625369	A	G	0.0925203	0.0123806	0.00303411	4.50E-05	89714	b-d-2261	1l:ukb-d-2	TRUE	reported	igd
s1240918	1	114055488	C	T	0.248688	-0.00854806	0.00207888	3.93E-05	89714	b-d-2261	1l:ukb-d-2	TRUE	reported	igd
s1204186	1	60484531	T	C	0.0802328	0.0139379	0.00324288	1.73E-05	89714	b-d-2261	1l:ukb-d-2	TRUE	reported	igd
s37087095	1	245981170	A	G	0.0507364	0.0164857	0.00402385	4.19E-05	89714	b-d-2261	1l:ukb-d-2	TRUE	reported	igd
s55408012	2	42833020	A	G	0.0187471	-0.02879	0.00668861	1.68E-05	89714	b-d-2261	1l:ukb-d-2	TRUE	reported	igd
s1210571	2	60736985	G	T	0.368941	0.00874329	0.00184062	2.04E-06	89714	b-d-2261	1l:ukb-d-2	TRUE	reported	igd
rs4433952	2	238114802	T	A	0.431419	0.00779841	0.00181535	1.74E-05	89714	b-d-2261	1l:ukb-d-2	TRUE	reported	igd
s19246314	2	213744121	T	A	0.0195525	0.0266253	0.00643949	3.56E-05	89714	b-d-2261	1l:ukb-d-2	TRUE	reported	igd
s7440515	2	208711524	A	G	0.0257119	-0.0228533	0.00563455	5.00E-05	89714	b-d-2261	1l:ukb-d-2	TRUE	reported	igd
rs1714946	2	235265207	G	A	0.0894473	0.0138888	0.00313598	9.49E-06	89714	b-d-2261	1l:ukb-d-2	TRUE	reported	igd
s7463978	2	144885367	T	A	0.089228	0.012535	0.00307039	4.46E-05	89714	b-d-2261	1l:ukb-d-2	TRUE	reported	igd
rs876462	2	37515880	G	T	0.374342	-0.00799968	0.00181056	9.96E-06	89714	b-d-2261	1l:ukb-d-2	TRUE	reported	igd
s1249329	3	16679114	G	T	0.0537133	0.016262	0.00388067	2.79E-05	89714	b-d-2261	1l:ukb-d-2	TRUE	reported	igd
rs4688298	3	62460883	T	C	0.0741433	0.0139914	0.00340649	4.01E-05	89714	b-d-2261	1l:ukb-d-2	TRUE	reported	igd
s15010006	3	96018959	C	T	0.020231	0.0288195	0.00671373	1.77E-05	89714	b-d-2261	1l:ukb-d-2	TRUE	reported	igd
s11229281	3	48994119	A	G	0.943205	-0.0186685	0.00402318	3.48E-06	89714	b-d-2261	1l:ukb-d-2	TRUE	reported	igd
s11624962	3	190482826	T	C	0.0180067	-0.0288803	0.00697431	3.46E-05	89714	b-d-2261	1l:ukb-d-2	TRUE	reported	igd
rs3897921	3	101703056	T	G	0.430998	-0.00753564	0.00177005	2.07E-05	89714	b-d-2261	1l:ukb-d-2	TRUE	reported	igd
s14447977	3	122728248	A	G	0.0047649	0.0583193	0.0129703	6.92E-06	89714	b-d-2261	1l:ukb-d-2	TRUE	reported	igd
s3538577	3	196071832	T	A	0.634647	0.00794077	0.00182508	1.36E-05	89714	b-d-2261	1l:ukb-d-2	TRUE	reported	igd
rs4975012	4	39423512	A	G	0.22088	0.00902603	0.00212907	2.24E-05	89714	b-d-2261	1l:ukb-d-2	TRUE	reported	igd
s1000908	4	180336832	A	G	0.535987	-0.00764226	0.00176045	1.42E-05	89714	b-d-2261	1l:ukb-d-2	TRUE	reported	igd
s14977274	4	12576228	T	A	0.0204095	0.0282039	0.0066206	2.05E-05	89714	b-d-2261	1l:ukb-d-2	TRUE	reported	igd
s18987981	4	69058096	T	G	0.0165783	0.0292588	0.00705273	3.35E-05	89714	b-d-2261	1l:ukb-d-2	TRUE	reported	igd
s20054815	5	130378567	A	C	0.0157194	0.030137	0.00735607	4.19E-05	89714	b-d-2261	1l:ukb-d-2	TRUE	reported	igd
rs6881290	5	11147494	C	T	0.583869	0.00745342	0.00177421	2.66E-05	89714	b-d-2261	1l:ukb-d-2	TRUE	reported	igd
rs9368351	6	21808192	T	A	0.0147391	0.0301738	0.00733453	3.89E-05	89714	b-d-2261	1l:ukb-d-2	TRUE	reported	igd
s7780552	6	144834312	T	G	0.0576392	0.0193191	0.00379766	3.64E-07	89714	b-d-2261	1l:ukb-d-2	TRUE	reported	igd
rs6914902	6	139021524	C	A	0.149032	0.0102618	0.00248621	3.67E-05	89714	b-d-2261	1l:ukb-d-2	TRUE	reported	igd
rs1262928	6	16806082	A	T	0.332231	-0.00811472	0.00188608	1.69E-05	89714	b-d-2261	1l:ukb-d-2	TRUE	reported	igd
s14965131	6	160595479	G	C	0.0117592	0.0381334	0.00835709	5.05E-06	89714	b-d-2261	1l:ukb-d-2	TRUE	reported	igd
rs3093540	6	31539010	A	G	0.0463158	-0.0184646	0.004175	9.76E-06	89714	b-d-2261	1l:ukb-d-2	TRUE	reported	igd
s3592179	6	98693636	A	G	0.409366	0.00732231	0.00179381	4.47E-05	89714	b-d-2261	1l:ukb-d-2	TRUE	reported	igd
s11785805	6	149289638	A	G	0.0362675	-0.0212002	0.00484085	1.19E-05	89714	b-d-2261	1l:ukb-d-2	TRUE	reported	igd
s5760534	6	151835770	C	G	0.0277103	-0.0217696	0.0053386	4.55E-05	89714	b-d-2261	1l:ukb-d-2	TRUE	reported	igd
s1094513	6	69354524	A	T	0.313689	-0.00785951	0.00188914	3.18E-05	89714	b-d-2261	1l:ukb-d-2	TRUE	reported	igd
s11413453	6	41851676	T	C	0.00944719	0.0403285	0.00973382	3.43E-05	89714	b-d-2261	1l:ukb-d-2	TRUE	reported	igd
s7978084	7	104247958	C	T	0.0226911	0.0247413	0.0059315	3.03E-05	89714	b-d-2261	1l:ukb-d-2	TRUE	reported	igd
s11216968	7	70641982	A	G	0.0169427	0.030187	0.00676279	8.07E-06	89714	b-d-2261	1l:ukb-d-2	TRUE	reported	igd
s14325528	7	147431844	C	T	0.0481414	0.0169392	0.00412782	4.07E-05	89714	b-d-2261	1l:ukb-d-2	TRUE	reported	igd
s7730979	7	73663178	A	C	0.0155521	0.0292122	0.00711909	4.08E-05	89714	b-d-2261	1l:ukb-d-2	TRUE	reported	igd
s6247730	7	114935274	T	A	0.0448565	0.0201649	0.00425824	2.19E-06	89714	b-d-2261	1l:ukb-d-2	TRUE	reported	igd
s14756794	7	47544762	A	G	0.039827	0.0194716	0.00470387	3.48E-05	89714	b-d-2261	1l:ukb-d-2	TRUE	reported	igd
s57741147	8	109585516	G	T	0.0181501	0.0317945	0.00707126	6.92E-06	89714	b-d-2261	1l:ukb-d-2	TRUE	reported	igd
rs3106532	8	131259140	A	C	0.106488	0.0121055	0.00284134	2.04E-05	89714	b-d-2261	1l:ukb-d-2	TRUE	reported	igd
rs3817706	8	2054195	G	C	0.576263	-0.00722197	0.00177759	4.85E-05	89714	b-d-2261	1l:ukb-d-2	TRUE	reported	igd
s7949864	8	134855286	G	A	0.0229563	-0.0277196	0.00583247	2.01E-06	89714	b-d-2261	1l:ukb-d-2	TRUE	reported	igd
s5581366	8	62318022	T	C	0.0148805	0.0310402	0.00725634	1.89E-05	89714	b-d-2261	1l:ukb-d-2	TRUE	reported	igd
s7621785	8	94600393	G	A	0.00962503	0.0440065	0.00897803	9.52E-07	89714	b-d-2261	1l:ukb-d-2	TRUE	reported	igd
s11814611	8	22116219	G	A	0.0938635	-0.0131471	0.00311966	2.51E-05	89714	b-d-2261	1l:ukb-d-2	TRUE	reported	igd
s6249616	8	9288470	T	C	0.0217623	0.0283802	0.00632286	7.18E-06	89714	b-d-2261	1l:ukb-d-2	TRUE	reported	igd
rs3739699	9	114798940	A	G	0.496407	0.00727057	0.00176661	3.87E-05	89714	b-d-2261	1l:ukb-d-2	TRUE	reported	igd
s7272605	9	38072104	G	C	0.125096	-0.0115428	0.0026655	1.49E-05	89714	b-d-2261	1l:ukb-d-2	TRUE	reported	igd
rs884258	9	107057177	C	T	0.67693	-0.00785979	0.00189426	3.34E-05	89714	b-d-2261	1l:ukb-d-2	TRUE	reported	igd
rs2031468	10	131923448	C	T	0.623911	0.00757944	0.00181432	2.95E-05	89714	b-d-2261	1l:ukb-d-2	TRUE	reported	igd
s1276530	10	7631014	T	C	0.232411	0.00867476	0.00210372	3.73E-05	89714	b-d-2261	1l:ukb-d-2	TRUE	reported	igd
rs7082468	10	50486727	A	G	0.678908	-0.00849508	0.00187987	6.22E-06	89714	b-d-2261	1l:ukb-d-2	TRUE	reported	igd
rs1993415	10	63400853	G	C	0.253389	0.00829699	0.00201769	3.92E-05	89714	b-d-2261	1l:ukb-d-2	TRUE	reported	igd
s7687941	10	87990995	T	C	0.0269579	-0.0226667	0.00540836	2.78E-05	89714	b-d-2261	1l:ukb-d-2	TRUE	reported	igd
rs513564	10	99780908	T	C	0.249954	0.00846321	0.00207117	4.39E-05	89714	b-d-2261	1l:ukb-d-2	TRUE	reported	igd
rs2765837	10	21034317	A	G	0.120364	0.0127686	0.00272239	2.73E-06	89714	b-d-2261	1l:ukb-d-2	TRUE	reported	igd
rs2847178	11	105877624	T	C	0.634157	0.00882033	0.00183024	1.44E-06	89714	b-d-2261	1l:ukb-d-2	TRUE	reported	igd
s7289614	11	5845481	A	G	0.108101	0.0117965	0.00290823	4.99E-05	89714	b-d-2261	1l:ukb-d-2	TRUE	reported	igd
s1021937	11	86339724	T	C	0.190195	-0.00945261	0.0022387	2.42E-05	89714	b-d-2261	1l:ukb-d-2	TRUE	reported	igd
s3513135	11	29139477	G	A	0.21688	-0.010796	0.00220881	1.02E-06	89714	b-d-2261	1l:ukb-d-2	TRUE	reported	igd
rs2069408	12	56364321	G	A	0.340928	-0.00839198	0.00184948	5.70E-06	89714	b-d-2261	1l:ukb-d-2	TRUE	reported	igd
rs1458176	12	41777144	T	C	0.549556	0.0080669	0.00175604	4.36E-06	89714	b-d-2261	1l:ukb-d-2	TRUE	reported	igd
s5616940	12	117155472	T	C	0.0735564	-0.0141072	0.00347247	4.86E-05	89714	b-d-2261	1l:ukb-d-2	TRUE	reported	igd
s7321416	12	121476099	C	A	0.247631	-0.00875473	0.00203417	1.68E-05	89714	b-d-2261	1l:ukb-d-2	TRUE	reported	igd
s1760263	12	42480604	A	G	0.0158631	0.0335219	0.00714406	2.71E-06	89714	b-d-2261	1l:ukb-d-2	TRUE	reported	igd
s1286692	13	110066103	A	G	0.023507	0.0252359	0.00578864	1.30E-05	89714	b-d-2261	1l:ukb-d-2	TRUE	reported	igd
s14256388	13	23278334	A	G	0.0147267	-0.0343881	0.00745485	3.98E-06	89714	b-d-2261	1l:ukb-d-2	TRUE	reported	igd
rs9530332	13	75070201	G	T	0.654539	0.00769591	0.00184866	3.14E-05	89714	b-d-2261	1l:ukb-d-2	TRUE	reported	igd
rs7336192	13	53911048	C	T	0.442393	-0.00726867	0.00177597	4.27E-05	89714	b-d-2261	1l:ukb-d-2	TRUE	reported	igd
rs2296498	13	95096111	G	A	0.114016	-0.0113163	0.00276525	4.27E-05	89714	b-d-2261	1l:ukb-d-2	TRUE	reported	igd
s15068904	13	23446586	A	G	0.0145037	0.0322386	0.00765178	2.52E-05	89714	b-d-2261	1l:ukb-d-2	TRUE	reported	igd
s7951953	14	34720312	C	T	0.0289475	-0.0217882	0.00520882	2.88E-05	89714	b-d-2261	1l:ukb-d-2	TRUE	reported	igd
rs8006552	14	67294069	C	G	0.15964	0.0124305	0.00244429	3.67E-07	89714	b-d-2261	1l:ukb-d-2	TRUE	reported	igd
rs2683122	15	36024804	C	A	0.465836	-0.00719049	0.00177068	4.89E-05	89714	b-d-2261	1l:ukb-d-2	TRUE	reported	igd
s13885819	15	79099986	A	G	0.0073634	0.0430825	0.0104699	3.88E-05	89714	b-d-2261	1l:ukb-d-2	TRUE	reported	igd
rs3825941	15	74317362	A	G	0.257769	0.00829075	0.00201638	3.93E-05	89714	b-d-2261	1l:ukb-d-2	TRUE	reported	igd
s6733610	15	60352204	C	G	0.214855	-0.00984579	0.00216755	5.57E-06	89714	b-d-2261	1l:ukb-d-2	TRUE	reported	igd
s11741092	15	47979695	G	A	0.00888031	-0.0415391	0.00945298	1.11E-05	89714	b-d-2261	1l:ukb-d-2	TRUE	reported	igd
s7276033	15	84192374	C	T	0.014088	0.0314433	0.00757947	3.35E-05	89714	b-d-2261	1l:ukb-d-2	TRUE	reported	igd
rs7204542	16	718514	G	C	0.691831	0.00922209	0.00196696	2.76E-06	89714	b-d-2261	1l:ukb-d-2	TRUE	reported	igd
s55928042	16	69750910	A	G	0.025008	-0.0260141	0.00565598	4.24E-06	89714	b-d-2261	1l:ukb-d-2	TRUE	reported	igd
s6205538	16	30187673	G	T	0.0872412	-0.0139716	0.00321154	1.36E-05	89714	b-d-2261	1l:ukb-d-2	TRUE	reported	igd
rs7201235	16	19532063	A	G	0.0863941	-0.0144375	0.00345182	2.88E-05	89714	b-d-2261	1l:ukb-d-2	TRUE	reported	igd
rs4131945	16	54862861	T	G	0.513709	-0.00740657	0.00177911	3.14E-05	89714	b-d-2261	1l:ukb-d-2	TRUE	reported	igd
rs772112	17	33621127	A	T	0.868737	0.0113452	0.00264721	1.82E-05	89714	b-d-2261	1l:ukb-d-2	TRUE	reported	igd
rs689896	18	74078911	G	C	0.637688	0.0076189	0.00182037	2.85E-05	89714	b-d-2261	1l:ukb-d-2	TRUE	reported	igd
s1042554	19	836043	C	T	0.707831	-0.00843132	0.00204125	3.62E-05	89714	b-d-2261	1l:ukb-d-2	TRUE	reported	igd
s14064772	19	20290042	G	A	0.00760469	0.0437941	0.0105942	3.57E-05	89714	b-d-2261	1l:ukb-d-2	TRUE	reported	igd
s19223036	19	50188478	A	G	0.00158976	0.0994673	0.0234151	2.16E-05	89714	b-d-2261	1l:ukb-d-2	TRUE	reported	igd
rs6112545	20	19727485	T	C	0.0884728	-0.015143	0.00307712	8.62E-07	89714	b-d-2261	1l:ukb-d-2	TRUE	reported	igd
s6220800	20	62043227	G	A	0.0223098	0.0254917	0.00591581	1.64E-05	89714	b-d-2261	1l:ukb-d-2	TRUE	reported	igd
rs6130609	20	43033095	C	G	0.190034	-0.0103184	0.00223292	3.82E-06	89714	b-d-2261	1l:ukb-d-2	TRUE	reported	igd
rs4814452	20	16178198	C	T	0.945961	-0.0167929	0.00387384	1.46E-05	89714	b-d-2261	1l:ukb-d-2	TRUE	reported	igd
rs914186	21	42619657	A	G	0.660059	0.00772703	0.00186074	3.29E-05	89714	b-d-2261	1l:ukb-d-2	TRUE	reported	igd
rs207008	21	18735936	G	T	0.941882	-0.0195589	0.00375031	1.84E-07	89714	b-d-2261	1l:ukb-d-2	TRUE	reported	igd
s7336010	21	41403037	C	T	0.250511	-0.00848229	0.00203355	3.03E-05	89714	b-d-2261	1l:ukb-d-2	TRUE	reported	igd
s11809819	21	33944718	T	C	0.0201729	0.0298263	0.00655698	5.40E-06	89714	b-d-2261	1l:ukb-d-2	TRUE	reported	igd

·s1248406	22	51099862	T	C	0.205871	0.00904834	0.00216889	3.02E-05	89714	b-d-22611l:ukb-d-2	TRUE	reported	igd
s11751402	22	49534383	T	G	0.189881	0.00909136	0.00222923	4.54E-05	89714	b-d-22611l:ukb-d-2	TRUE	reported	igd
s14890409	22	25249949	C	G	0.0151067	0.0326451	0.00759825	1.74E-05	89714	b-d-22611l:ukb-d-2	TRUE	reported	igd

Table S2. Detail Information of the Genetic Variants in the MR Analysis of SHS and GRED

SNP	effect a	other al	effect a	other al	beta.exp	beta.out	eaf.expo	eaf.outc	remove	palindro	ambiguou	id.outco	chr	pos	se.outco	samplesi	pval.out	outcome	original	outcome.	mr keep.	data sou	pos.exposu	se.expos	pval.exp	chr.expo	samplesi	id.expos	exposure	mr keep.	pval ori	data sou	action	mr keep
rs100090	A	G	A	G	-0.0076	-0.0141	0.53599	0.5929	FALSE	FALSE	FALSE	ebi-a-GC	4	180336832	0.0083	467253	0.08894	Gastroes	Gastroes	Gastroes	TRUE	igd	180336832	0.00176	1.4E-05	4	89714	ukb-d-22	|| id:u	TRUE	reported	igd	2	TRUE
rs102193	T	C	T	C	-0.0095	0.0087	0.1902	0.21758	FALSE	FALSE	FALSE	ebi-a-GC	11	86339724	0.0109	467253	0.4248	Gastroes	Gastroes	Gastroes	TRUE	igd	86339724	0.00224	2.4E-05	11	89714	ukb-d-22	|| id:u	TRUE	reported	igd	2	TRUE
rs104255	C	T	C	T	-0.0084	-0.0131	0.70783	0.72588	FALSE	FALSE	FALSE	ebi-a-GC	19	836043	0.0098	467253	0.1842	Gastroes	Gastroes	Gastroes	TRUE	igd	836043	0.00204	3.6E-05	19	89714	ukb-d-22	|| id:u	TRUE	reported	igd	2	TRUE
rs109451	A	T	A	T	-0.0079	-0.013	0.31369	0.30773	FALSE	TRUE	FALSE	ebi-a-GC	6	69354524	0.0091	467253	0.1526	Gastroes	Gastroes	Gastroes	TRUE	igd	69354524	0.00189	3.2E-05	6	89714	ukb-d-22	|| id:u	TRUE	reported	igd	2	TRUE
rs112169	A	G	A	G	0.03019	0.0642	0.01694	0.01539	FALSE	FALSE	FALSE	ebi-a-GC	7	70641982	0.0339	467253	0.05796	Gastroes	Gastroes	Gastroes	TRUE	igd	70641982	0.00676	8.1E-06	7	89714	ukb-d-22	|| id:u	TRUE	reported	igd	2	TRUE
rs112292	A	G	A	G	-0.0187	-0.0219	0.94321	0.94205	FALSE	FALSE	FALSE	ebi-a-GC	3	48994119	0.0223	467253	0.3255	Gastroes	Gastroes	Gastroes	TRUE	igd	48994119	0.00402	3.5E-06	3	89714	ukb-d-22	|| id:u	TRUE	reported	igd	2	TRUE
rs114134	T	C	T	C	0.04033	-0.09	0.00945	0.00752	FALSE	FALSE	FALSE	ebi-a-GC	6	41851676	0.051	467253	0.07797	Gastroes	Gastroes	Gastroes	TRUE	igd	41851676	0.00973	3.4E-05	6	89714	ukb-d-22	|| id:u	TRUE	reported	igd	2	TRUE
rs116249	T	C	T	C	-0.0289	-0.0036	0.01801	0.01664	FALSE	FALSE	FALSE	ebi-a-GC	3	190482826	0.0342	467253	0.9166	Gastroes	Gastroes	Gastroes	TRUE	igd	190482826	0.00697	3.5E-05	3	89714	ukb-d-22	|| id:u	TRUE	reported	igd	2	TRUE
rs117410	G	A	G	A	-0.0415	-0.0293	0.00888	0.00702	FALSE	FALSE	FALSE	ebi-a-GC	15	47979695	0.0445	467253	0.5098	Gastroes	Gastroes	Gastroes	TRUE	igd	47979695	0.00945	1.1E-05	15	89714	ukb-d-22	|| id:u	TRUE	reported	igd	2	TRUE
rs117514	T	G	T	G	0.00909	-0.0068	0.18988	0.1898	FALSE	FALSE	FALSE	ebi-a-GC	22	49534383	0.0122	467253	0.5757	Gastroes	Gastroes	Gastroes	TRUE	igd	49534383	0.00223	4.5E-05	22	89714	ukb-d-22	|| id:u	TRUE	reported	igd	2	TRUE
rs117858	A	G	A	G	-0.0212	-0.0068	0.03627	0.03785	FALSE	FALSE	FALSE	ebi-a-GC	6	149289638	0.0226	467253	0.764	Gastroes	Gastroes	Gastroes	TRUE	igd	149289638	0.00484	1.2E-05	6	89714	ukb-d-22	|| id:u	TRUE	reported	igd	2	TRUE
rs118098	T	C	T	C	0.02983	0.063	0.02017	0.02074	FALSE	FALSE	FALSE	ebi-a-GC	21	33944718	0.0306	467253	0.03974	Gastroes	Gastroes	Gastroes	TRUE	igd	33944718	0.00656	5.4E-06	21	89714	ukb-d-22	|| id:u	TRUE	reported	igd	2	TRUE
rs118146	G	A	G	A	-0.0131	0.012	0.09386	0.09443	FALSE	FALSE	FALSE	ebi-a-GC	8	22116219	0.0148	467253	0.4188	Gastroes	Gastroes	Gastroes	TRUE	igd	22116219	0.00312	2.5E-05	8	89714	ukb-d-22	|| id:u	TRUE	reported	igd	2	TRUE
rs120418	T	C	T	C	0.01394	-0.0086	0.08023	0.11729	FALSE	FALSE	FALSE	ebi-a-GC	1	60484531	0.0148	467253	0.5621	Gastroes	Gastroes	Gastroes	TRUE	igd	60484531	0.00324	1.7E-05	1	89714	ukb-d-22	|| id:u	TRUE	reported	igd	2	TRUE
rs121057	G	T	G	T	0.00874	0.0021	0.36894	0.27114	FALSE	FALSE	FALSE	ebi-a-GC	2	60736985	0.0088	467253	0.8079	Gastroes	Gastroes	Gastroes	TRUE	igd	60736985	0.00184	2E-06	2	89714	ukb-d-22	|| id:u	TRUE	reported	igd	2	TRUE
rs124091	C	T	C	T	-0.0085	-0.0078	0.24869	0.19589	FALSE	FALSE	FALSE	ebi-a-GC	1	114055488	0.0099	467253	0.4291	Gastroes	Gastroes	Gastroes	TRUE	igd	114055488	0.00208	3.9E-05	1	89714	ukb-d-22	|| id:u	TRUE	reported	igd	2	TRUE
rs124840	T	C	T	C	0.00905	-0.0073	0.20587	0.29988	FALSE	FALSE	FALSE	ebi-a-GC	22	51099862	0.0098	467253	0.457	Gastroes	Gastroes	Gastroes	TRUE	igd	51099862	0.00217	3E-05	22	89714	ukb-d-22	|| id:u	TRUE	reported	igd	2	TRUE
rs124932	G	T	G	T	0.01626	0.0046	0.05371	0.05143	FALSE	FALSE	FALSE	ebi-a-GC	3	16679114	0.0194	467253	0.8131	Gastroes	Gastroes	Gastroes	TRUE	igd	16679114	0.00388	2.8E-05	3	89714	ukb-d-22	|| id:u	TRUE	reported	igd	2	TRUE
rs126292	A	T	A	T	-0.0081	0.0064	0.33223	0.23704	FALSE	TRUE	FALSE	ebi-a-GC	6	16806082	0.0092	467253	0.4871	Gastroes	Gastroes	Gastroes	TRUE	igd	16806082	0.00189	1.7E-05	6	89714	ukb-d-22	|| id:u	TRUE	reported	igd	2	TRUE
rs127653	T	C	T	C	0.00867	-0.0102	0.23241	0.27388	FALSE	FALSE	FALSE	ebi-a-GC	10	7631014	0.0098	467253	0.2954	Gastroes	Gastroes	Gastroes	TRUE	igd	7631014	0.0021	3.7E-05	10	89714	ukb-d-22	|| id:u	TRUE	reported	igd	2	TRUE
rs128669	A	G	A	G	0.02524	0.01	0.02351	0.03291	FALSE	FALSE	FALSE	ebi-a-GC	13	110066103	0.0262	467253	0.7023	Gastroes	Gastroes	Gastroes	TRUE	igd	110066103	0.00579	1.3E-05	13	89714	ukb-d-22	|| id:u	TRUE	reported	igd	2	TRUE
rs138858	A	G	A	G	0.04308	0.0144	0.00736	0.01383	FALSE	FALSE	FALSE	ebi-a-GC	15	79099986	0.0366	467253	0.6939	Gastroes	Gastroes	Gastroes	TRUE	igd	79099986	0.01047	3.9E-05	15	89714	ukb-d-22	|| id:u	TRUE	reported	igd	2	TRUE
rs140647	G	A	G	A	0.04379	-0.0448	0.0076	0.00985	FALSE	FALSE	FALSE	ebi-a-GC	19	20290042	0.0447	467253	0.3165	Gastroes	Gastroes	Gastroes	TRUE	igd	20290042	0.01059	3.6E-05	19	89714	ukb-d-22	|| id:u	TRUE	reported	igd	2	TRUE
rs142563	A	G	A	G	-0.0344	-0.0547	0.01473	0.01323	FALSE	FALSE	FALSE	ebi-a-GC	13	23278334	0.0339	467253	0.1065	Gastroes	Gastroes	Gastroes	TRUE	igd	23278334	0.00745	4E-06	13	89714	ukb-d-22	|| id:u	TRUE	reported	igd	2	TRUE
rs143255	C	T	C	T	0.01694	-0.0145	0.04814	0.04785	FALSE	FALSE	FALSE	ebi-a-GC	7	147431844	0.0198	467253	0.4646	Gastroes	Gastroes	Gastroes	TRUE	igd	147431844	0.00413	4.1E-05	7	89714	ukb-d-22	|| id:u	TRUE	reported	igd	2	TRUE
rs144479	A	G	A	G	0.05832	-0.0413	0.00476	0.00524	FALSE	FALSE	FALSE	ebi-a-GC	3	122728248	0.0597	467253	0.4893	Gastroes	Gastroes	Gastroes	TRUE	igd	122728248	0.01297	6.9E-06	3	89714	ukb-d-22	|| id:u	TRUE	reported	igd	2	TRUE
rs145817	T	C	T	C	0.00807	0.0053	0.54956	0.63581	FALSE	FALSE	FALSE	ebi-a-GC	12	41777144	0.0084	467253	0.5225	Gastroes	Gastroes	Gastroes	TRUE	igd	41777144	0.00176	4.4E-06	12	89714	ukb-d-22	|| id:u	TRUE	reported	igd	2	TRUE
rs147567	A	G	A	G	0.01947	-0.0486	0.03983	0.03146	FALSE	FALSE	FALSE	ebi-a-GC	7	47544762	0.0247	467253	0.04941	Gastroes	Gastroes	Gastroes	TRUE	igd	47544762	0.0047	3.5E-05	7	89714	ukb-d-22	|| id:u	TRUE	reported	igd	2	TRUE
rs148904	C	G	C	G	0.03265	-0.0176	0.01511	0.01248	FALSE	TRUE	FALSE	ebi-a-GC	22	25249949	0.0391	467253	0.6534	Gastroes	Gastroes	Gastroes	TRUE	igd	25249949	0.0076	1.7E-05	22	89714	ukb-d-22	|| id:u	TRUE	reported	igd	2	TRUE
rs149651	G	C	G	C	0.03813	-0.0487	0.01176	0.01223	FALSE	TRUE	FALSE	ebi-a-GC	6	160595479	0.0394	467253	0.2165	Gastroes	Gastroes	Gastroes	TRUE	igd	160595479	0.00836	5E-06	6	89714	ukb-d-22	|| id:u	TRUE	reported	igd	2	TRUE
rs149772	T	A	T	A	0.0282	-0.0257	0.02041	0.01699	FALSE	TRUE	FALSE	ebi-a-GC	4	12576228	0.034	467253	0.4508	Gastroes	Gastroes	Gastroes	TRUE	igd	12576228	0.00662	2E-05	4	89714	ukb-d-22	|| id:u	TRUE	reported	igd	2	TRUE
rs150100	C	T	C	T	0.02882	-0.0127	0.02023	0.01808	FALSE	FALSE	FALSE	ebi-a-GC	3	96018959	0.0333	467253	0.7031	Gastroes	Gastroes	Gastroes	TRUE	igd	96018959	0.00671	1.8E-05	3	89714	ukb-d-22	|| id:u	TRUE	reported	igd	2	TRUE
rs171494	G	A	G	A	0.01389	-0.0217	0.08945	0.09925	FALSE	FALSE	FALSE	ebi-a-GC	2	235265207	0.0143	467253	0.1299	Gastroes	Gastroes	Gastroes	TRUE	igd	235265207	0.00314	9.5E-06	2	89714	ukb-d-22	|| id:u	TRUE	reported	igd	2	TRUE
rs176026	A	G	A	G	0.03352	0.0209	0.01586	0.01624	FALSE	FALSE	FALSE	ebi-a-GC	12	42688114	0.034	467253	0.5399	Gastroes	Gastroes	Gastroes	TRUE	igd	42480604	0.00714	2.7E-06	12	89714	ukb-d-22	|| id:u	TRUE	reported	igd	2	TRUE
rs189879	T	G	T	G	0.02926	-0.0072	0.01658	0.01618	FALSE	FALSE	FALSE	ebi-a-GC	4	69058096	0.0339	467253	0.8314	Gastroes	Gastroes	Gastroes	TRUE	igd	69058096	0.00705	3.3E-05	4	89714	ukb-d-22	|| id:u	TRUE	reported	igd	2	TRUE
rs192230	A	G	A	G	0.09947	-0.0039	0.00159	0.01487	FALSE	FALSE	FALSE	ebi-a-GC	19	50188478	0.0385	467253	0.9193	Gastroes	Gastroes	Gastroes	TRUE	igd	50188478	0.02342	2.2E-05	19	89714	ukb-d-22	|| id:u	TRUE	reported	igd	2	TRUE
rs192463	T	A	T	A	0.02663	-0.0138	0.01955	0.01653	FALSE	TRUE	FALSE	ebi-a-GC	2	213744121	0.0331	467253	0.6767	Gastroes	Gastroes	Gastroes	TRUE	igd	213744121	0.00644	3.6E-05	2	89714	ukb-d-22	|| id:u	TRUE	reported	igd	2	TRUE
rs199341	G	C	G	C	0.0083	-0.0176	0.25339	0.2009	FALSE	TRUE	FALSE	ebi-a-GC	10	63400853	0.0097	467253	0.06773	Gastroes	Gastroes	Gastroes	TRUE	igd	63400853	0.00202	3.9E-05	10	89714	ukb-d-22	|| id:u	TRUE	reported	igd	2	TRUE
rs200548	A	C	A	C	0.03014	-0.0401	0.01572	0.01563	FALSE	FALSE	FALSE	ebi-a-GC	5	130378567	0.0402	467253	0.3191	Gastroes	Gastroes	Gastroes	TRUE	igd	130378567	0.00736	4.2E-05	5	89714	ukb-d-22	|| id:u	TRUE	reported	igd	2	TRUE
rs203146	C	T	C	T	0.00758	0.0011	0.62391	0.68844	FALSE	FALSE	FALSE	ebi-a-GC	10	131923448	0.0088	467253	0.9046	Gastroes	Gastroes	Gastroes	TRUE	igd	131923448	0.00181	2.9E-05	10	89714	ukb-d-22	|| id:u	TRUE	reported	igd	2	TRUE
rs206940	G	A	G	A	-0.0084	-0.0301	0.34093	0.3051	FALSE	FALSE	FALSE	ebi-a-GC	12	56364321	0.0088	467253	0.00061	Gastroes	Gastroes	Gastroes	TRUE	igd	56364321	0.00185	5.7E-06	12	89714	ukb-d-22	|| id:u	TRUE	reported	igd	2	TRUE
rs207008	G	T	G	T	-0.0196	0.0081	0.94188	0.94412	FALSE	FALSE	FALSE	ebi-a-GC	21	18735936	0.0183	467253	0.6574	Gastroes	Gastroes	Gastroes	TRUE	igd	18735936	0.00375	1.8E-07	21	89714	ukb-d-22	|| id:u	TRUE	reported	igd	2	TRUE
rs229649	G	A	G	A	-0.0113	0.0033	0.11402	0.12679	FALSE	FALSE	FALSE	ebi-a-GC	13	95096111	0.0128	467253	0.7967	Gastroes	Gastroes	Gastroes	TRUE	igd	95096111	0.00277	4.3E-05	13	89714	ukb-d-22	|| id:u	TRUE	reported	igd	2	TRUE
rs268312	C	A	C	A	-0.0072	0.0187	0.46584	0.47871	FALSE	FALSE	FALSE	ebi-a-GC	15	36024804	0.0083	467253	0.0248	Gastroes	Gastroes	Gastroes	TRUE	igd	36024804	0.00177	4.9E-05	15	89714	ukb-d-22	|| id:u	TRUE	reported	igd	2	TRUE
rs276583	A	G	A	G	0.01277	-0.0113	0.12036	0.10927	FALSE	FALSE	FALSE	ebi-a-GC	10	21034317	0.0135	467253	0.4008	Gastroes	Gastroes	Gastroes	TRUE	igd	21034317	0.00272	2.7E-06	10	89714	ukb-d-22	|| id:u	TRUE	reported	igd	2	TRUE
rs284717	T	C	T	C	0.00882	0.001	0.63416	0.6653	FALSE	FALSE	FALSE	ebi-a-GC	11	105877624	0.0086	467253	0.9049	Gastroes	Gastroes	Gastroes	TRUE	igd	105877624	0.00183	1.4E-06	11	89714	ukb-d-22	|| id:u	TRUE	reported	igd	2	TRUE
rs309354	A	G	A	G	-0.0185	0.0577	0.04632	0.05363	FALSE	FALSE	FALSE	ebi-a-GC	6	31539010	0.0194	467253	0.00295	Gastroes	Gastroes	Gastroes	TRUE	igd	31539010	0.00418	9.8E-06	6	89714	ukb-d-22	|| id:u	TRUE	reported	igd	2	TRUE
rs310653	A	C	A	C	0.01211	-0.0044	0.10649	0.12115	FALSE	FALSE	FALSE	ebi-a-GC	8	131259140	0.013	467253	0.7329	Gastroes	Gastroes	Gastroes	TRUE	igd	131259140	0.00284	2E-05	8	89714	ukb-d-22	|| id:u	TRUE	reported	igd	2	TRUE
rs351313	G	A	G	A	-0.0108	0.0174	0.21688	0.23092	FALSE	FALSE	FALSE	ebi-a-GC	11	29139477	0.0118	467253	0.1399	Gastroes	Gastroes	Gastroes	TRUE	igd	29139477	0.00221	1E-06	11	89714	ukb-d-22	|| id:u	TRUE	reported	igd	2	TRUE
rs353857	T	A	T	A	0.00794	0.0144	0.63465	0.70032	FALSE	TRUE	FALSE	ebi-a-GC	3	196071832	0.0087	467253	0.09648	Gastroes	Gastroes	Gastroes	TRUE	igd	196071832	0.00183	1.4E-05	3	89714	ukb-d-22	|| id:u	TRUE	reported	igd	2	TRUE
rs359217	A	G	A	G	0.00732	0.0234	0.40937	0.41726	FALSE	FALSE	FALSE	ebi-a-GC	6	98693636	0.0096	467253	0.01454	Gastroes	Gastroes	Gastroes	TRUE	igd	98693636	0.00179	4.5E-05	6	89714	ukb-d-22	|| id:u	TRUE	reported	igd	2	TRUE
rs370870	A	G	A	G	0.01649	0.0104	0.05074	0.12524	FALSE	FALSE	FALSE	ebi-a-GC	1	245981170	0.0162	467253	0.524	Gastroes	Gastroes	Gastroes	TRUE	igd	245981170	0.00402	4.2E-05	1	89714	ukb-d-22	|| id:u	TRUE	reported	igd	2	TRUE
rs373969	A	G	A	G	0.00727	0.0006	0.49641	0.55235	FALSE	FALSE	FALSE	ebi-a-GC	9	114798940	0.0083	467253	0.9459	Gastroes	Gastroes	Gastroes	TRUE	igd	114798940	0.00177	3.9E-05	9	89714	ukb-d-22	|| id:u	TRUE	reported	igd	2	TRUE
rs382594	A	G	A	G	0.00829	-0.0002	0.25777	0.27889	FALSE	FALSE	FALSE	ebi-a-GC	15	74317362	0.0094	467253	0.9813	Gastroes	Gastroes	Gastroes	TRUE	igd	74317362	0.00202	3.9E-05	15	89714	ukb-d-22	|| id:u	TRUE	reported	igd	2	TRUE
rs389792	T	G	T	G	-0.0075	-0.0096	0.431	0.3471	FALSE	FALSE	FALSE	ebi-a-GC	3	101703056	0.0085	467253	0.2618	Gastroes	Gastroes	Gastroes	TRUE	igd	101703056	0.00177	2.1E-05	3	89714	ukb-d-22	|| id:u	TRUE	reported	igd	2	TRUE
rs413194	T	G	T	G	-0.0074	-0.0041	0.51371	0.54085	FALSE	FALSE	FALSE	ebi-a-GC	16	54862861	0.0084	467253	0.6246	Gastroes	Gastroes	Gastroes	TRUE	igd	54862861	0.00178	3.1E-05	16	89714	ukb-d-22	|| id:u	TRUE	reported	igd	2	TRUE
rs443395	T	A	T	A	0.0078	-0.0143	0.43142	0.43259	FALSE	TRUE	TRUE	ebi-a-GC	2	238114802	0.0099	467253	0.1489	Gastroes	Gastroes	Gastroes	TRUE	igd	238114802	0.00182	1.7E-05	2	89714	ukb-d-22	|| id:u	TRUE	reported	igd	2	FALSE
rs468829	T	C	T	C	0.01399	-0.006	0.07414	0.06734	FALSE	FALSE	FALSE	ebi-a-GC	3	62460883	0.0169	467253	0.7201	Gastroes	Gastroes	Gastroes	TRUE	igd	62460883	0.00341	4E-05	3	89714	ukb-d-22	|| id:u	TRUE	reported	igd	2	TRUE
rs481445	C	T	C	T	-0.0168	0.0176	0.94596	0.92092	FALSE	FALSE	FALSE	ebi-a-GC	20	16178198	0.0169	467253	0.2977	Gastroes	Gastroes	Gastroes	TRUE	igd	16178198	0.00387	1.5E-05	20	89714	ukb-d-22	|| id:u	TRUE	reported	igd	2	TRUE
rs497501	A	G	A	G	0.00903	-0.0021	0.22088	0.19349	FALSE	FALSE	FALSE	ebi-a-GC	4	39423512	0.01	467253	0.8303	Gastroes	Gastroes	Gastroes	TRUE	igd	39423512	0.00213	2.2E-05	4	89714	ukb-d-22	|| id:u	TRUE	reported	igd	2	TRUE
rs513564	T	C	T	C	0.00846	-0.0063	0.24995	0.22205	FALSE	FALSE	FALSE	ebi-a-GC	10	99780908	0.01	467253	0.5263	Gastroes	Gastroes	Gastroes	TRUE	igd	99780908	0.00207	4.4E-05	10	89714	ukb-d-22	|| id:u	TRUE	reported	igd	2	TRUE
rs554080	A	G	A	G	-0.0288	-0.064	0.01875	0.01866	FALSE	FALSE	FALSE	ebi-a-GC	2	42833020	0.0367	467253	0.08127	Gastroes	Gastroes	Gastroes	TRUE	igd	42833020	0.00669	1.7E-05	2	89714	ukb-d-22	|| id:u	TRUE	reported	igd	2	TRUE
rs554912	C	G	C	G	-0.0124	-0.0052	0.10323	0.10415	FALSE	TRUE	FALSE	ebi-a-GC	1	226735908	0.0158	467253	0.7419	Gastroes	Gastroes	Gastroes	TRUE	igd	226735908	0.00295	2.8E-05	1	89714	ukb-d-22	|| id:u	TRUE	reported	igd	2	TRUE
rs558136	T	C	T	C	0.03104	0.0134	0.01488	0.01444	FALSE	FALSE	FALSE	ebi-a-GC	8	62318022	0.0352	467253	0.7035	Gastroes	Gastroes	Gastroes	TRUE	igd	62318022	0.00726	1.9E-05	8	89714	ukb-d-22	|| id:u	TRUE	reported	igd	2	TRUE
rs559280	A	G	A	G	-0.026	-0.0379	0.02501	0.02276	FALSE	FALSE	FALSE	ebi-a-GC	16	69750910	0.028	467253	0.1769	Gastroes	Gastroes	Gastroes	TRUE	igd	69750910	0.00566	4.2E-06	16	89714	ukb-d-22	|| id:u	TRUE	reported	igd	2	TRUE
rs561694	T	C	T	C	-0.0141	0.005	0.07356	0.06904	FALSE	FALSE	FALSE	ebi-a-GC	12	117155472	0.017	467253	0.7703	Gastroes	Gastroes	Gastroes	TRUE	igd	117155472	0.00347	4.9E-05	12	89714	ukb-d-22	|| id:u	TRUE	reported	igd	2	TRUE
rs576053	C	G	C	G	-0.0218	0.0117	0.02771	0.11716	FALSE	TRUE	FALSE	ebi-a-GC	6	151835770	0.022	467253	0.5944	Gastroes	Gastroes	Gastroes	TRUE	igd	151835770	0.00534	4.6E-05	6	89714	ukb-d-22	|| id:u	TRUE	reported	igd	2	TRUE
rs577411	G	T	G	T	0.03179	-0.1025	0.01815	0.01773	FALSE	FALSE	FALSE	ebi-a-GC	8	109585516	0.0393	467253	0.00901	Gastroes	Gastroes	Gastroes	TRUE	igd	109585516	0.00707	6.9E-06	8	89714	ukb-d-22	|| id:u	TRUE	reported	igd	2	TRUE
rs611254	T	C	T	C	-0.0151	0.0133	0.08847	0.08067	FALSE	FALSE	FALSE	ebi-a-GC	20	19727485	0.0149	467253	0.3713	Gastroes	Gastroes	Gastroes	TRUE	igd	19727485	0.00308	8.6E-07	20	89714	ukb-d-22	|| id:u	TRUE	reported	igd	2	TRUE
rs613060	C	G	C	G	-0.0103	-0.0058	0.19003	0.3044	FALSE	TRUE	FALSE	ebi-a-GC	20	43033095	0.0102	467253	0.5678	Gastroes	Gastroes	Gastroes	TRUE	igd	43033095	0.00223	3.8E-06	20	89714	ukb-d-22	|| id:u	TRUE	reported	igd	2	TRUE
rs620553	G	T	G	T	-0.014	-0.0019	0.08724	0.08159	FALSE	FALSE	FALSE	ebi-a-GC	16	30187673	0.0157	467253	0.903	Gastroes	Gastroes	Gastroes	TRUE	igd	30187673	0.00321	1.4E-05	16	89714	ukb-d-22	|| id:u	TRUE	reported	igd	2	TRUE
rs622080	G	A	G	A	0.02549	0.034	0.02231	0.02102	FALSE	FALSE	FALSE	ebi-a-GC	20	62043227	0.029	467253	0.2424	Gastroes	Gastroes	Gastroes	TRUE	igd	62043227	0.00592	1.6E-05	20	89714	ukb-d-22	|| id:u	TRUE	reported	igd	2	TRUE
rs624773	T	A	T	A	0.02016	0.0073	0.04486	0.04455	FALSE	TRUE	FALSE	ebi-a-GC	7	114935274	0.0204	467253	0.7219	Gastroes	Gastroes	Gastroes	TRUE	igd	114935274	0.00426	2.2E-06	7	89714	ukb-d-22	|| id:u	TRUE	reported	igd	2	TRUE
rs624961	T	C	T	C	0.02838	0.0107	0.02176	0.01785	FALSE	FALSE	FALSE	ebi-a-GC	8	9288470	0.0327	467253	0.7436	Gastroes	Gastroes	Gastroes	TRUE	igd	9288470	0.00632	7.2E-06	8	89714	ukb-d-22	|| id:u	TRUE	reported	igd	2	TRUE
rs673361	C	G	C	G	-0.0098	0.0043	0.21486	0.21379	FALSE	TRUE	FALSE	ebi-a-GC	15	60352204	0.0119	467253	0.7193	Gastroes	Gastroes	Gastroes	TRUE	igd	60352204	0.00217	5.6E-06	15	89714	ukb-d-22	|| id:u	TRUE	reported	igd	2	TRUE
rs688129	C	T	C	T	0.00745	-0.002	0.58387	0.53595	FALSE	FALSE	FALSE	ebi-a-GC	5	11147494	0.0083	467253	0.814	Gastroes	Gastroes	Gastroes	TRUE	igd	11147494	0.00177	2.7E-05	5	89714	ukb-d-22	|| id:u	TRUE	reported	igd	2	TRUE
rs689896	G	C	G	C	0.00762	0.0018	0.63769	0.69889	FALSE	TRUE	FALSE	ebi-a-GC	18	74078911	0.0087	467253	0.8401	Gastroes	Gastroes	Gastroes	TRUE	igd	74078911	0.00182	2.8E-05	18	89714	ukb-d-22	|| id:u	TRUE	reported	igd	2	TRUE
rs691490	C	A	C	A	0.01026	-0.0051	0.14903	0.20025	FALSE	FALSE	FALSE	ebi-a-GC	6	139021524	0.0115	467253	0.6543	Gastroes	Gastroes	Gastroes	TRUE	igd	139021524	0.00249	3.7E-05	6	89714	ukb-d-22	|| id:u	TRUE	reported	igd	2	TRUE
rs708246	A	G	A	G	-0.0085	0.0028	0.67891	0.60582	FALSE	FALSE	FALSE	ebi-a-GC	10	50486727	0.0088	467253	0.7501	Gastroes	Gastroes	Gastroes	TRUE	igd	50486727	0.00188	6.2E-06	10	89714	ukb-d-22	|| id:u	TRUE	reported	igd	2	TRUE
rs720123	A	G	A	G	-0.0144	0.0109	0.08639	0.12464	FALSE	FALSE	FALSE	ebi-a-GC	16	19532063	0.0181	467253	0.5493	Gastroes	Gastroes	Gastroes	TRUE	igd	19532063	0.00345	2.9E-05	16	89714	ukb-d-22	|| id:u	TRUE	reported	igd	2	TRUE
rs720454	G	C	G	C	0.00922	0.0197	0.69183	0.5664	FALSE	TRUE	TRUE	ebi-a-GC	16	718514	0.009	467253	0.02908	Gastroes	Gastroes	Gastroes	TRUE	igd	718514	0.00197	2.8E-06	16	89714	ukb-d-22	|| id:u	TRUE	reported	igd	2	FALSE
rs726791	A	G	A	G	0.01238	0.0268	0.09252	0.11842	FALSE	FALSE	FALSE	ebi-a-GC	1	66625369	0.0133	467253	0.04398	Gastroes	Gastroes	Gastroes	TRUE	igd	66625369	0.00303	4.5E-05	1	89714	ukb-d-22	|| id:u	TRUE	reported	igd	2	TRUE
rs727260	G	C	G	C	-0.0115	-0.0011	0.1251	0.09477	FALSE	TRUE	FALSE	ebi-a-GC	9	38072104	0.0128	467253	0.9333	Gastroes	Gastroes	Gastroes	TRUE	igd	38072104	0.00267	1.5E-05	9	89714	ukb-d-22	|| id:u	TRUE	reported	igd	2	TRUE
rs727603	C	T	C	T	0.03144	0.0582	0.01409	0.01734	FALSE	FALSE	FALSE	ebi-a-GC	15	84192374	0.0328	467253	0.0762	Gastroes	Gastroes	Gastroes	TRUE	igd	84192374	0.00758	3.3E-05	15	89714	ukb-d-22	|| id:u	TRUE	reported	igd	2	TRUE
rs728961	A	G	A	G	0.0118	-0.012	0.1081	0.09666	FALSE	FALSE	FALSE	ebi-a-GC	11	5845481	0.0145	467253	0.4081	Gastroes	Gastroes	Gastroes	TRUE	igd	5845481	0.00291	5E-05	11	89714	ukb-d-22	|| id:u	TRUE	reported	igd	2	TRUE
rs732141	C	A	C	A	-0.0088	0.0034	0.24763	0.23438	FALSE	FALSE	FALSE	ebi-a-GC	12	121476099	0.0097	467253	0.73	Gastroes	Gastroes	Gastroes	TRUE	igd	121476099	0.00203	1.7E-05	12	89714	ukb-d-22	|| id:u	TRUE	reported	igd	2	TRUE
rs733601	C	T	C	T	-0.0085	0.0042	0.25051	0.199	FALSE	FALSE	FALSE	ebi-a-GC	21	41403037	0.0098	467253	0.6701	Gastroes	Gastroes	Gastroes	TRUE	igd	41403037	0.00203	3E-05	21	89714	ukb-d-22	|| id:u	TRUE	reported	igd	2	TRUE
rs733619	C	T	C	T	-0.0073	-0.0173	0.44239	0.44267	FALSE	FALSE	FALSE	ebi-a-GC	13	53911048	0.0097	467253	0.07258	Gastroes	Gastroes	Gastroes	TRUE	igd	53911048	0.00178	4.3E-05	13	89714	ukb-d-22	|| id:u	TRUE	reported	igd	2	TRUE
rs744051	A	G	A	G	-0.0229	-0.0353	0.02571	0.02782	FALSE	FALSE	FALSE	ebi-a-GC	2	208711524	0.026	467253	0.1749	Gastroes	Gastroes	Gastroes	TRUE	igd	208711524	0.00563	5E-05	2	89714	ukb-d-22	|| id:u	TRUE	reported	igd	2	TRUE
rs746397	T	A	T	A	0.01254	0.0167	0.08923	0.09746	FALSE	TRUE	FALSE	ebi-a-GC	2	144885367	0.0143	467253	0.2412	Gastroes	Gastroes	Gastroes	TRUE	igd	144885367	0.00307	4.5E-05	2	89714	ukb-d-22	|| id:u	TRUE	reported	igd	2	TRUE
rs762178	G	A	G	A	0.04401	0.0432	0.00963	0.01025	FALSE	FALSE	FALSE	ebi-a-GC	8	94600393	0.0418	467253	0.3012	Gastroes	Gastroes	Gastroes	TRUE	igd	94600393	0.00898	9.5E-07	8	89714	ukb-d-22	|| id:u	TRUE	reported	igd	2	TRUE
rs768794	T	C	T	C	-0.0227	0.0175	0.02696	0.02215	FALSE	FALSE	FALSE	ebi-a-GC	10	87990995	0.0281	467253	0.5334	Gastroes	Gastroes	Gastroes	TRUE	igd	87990995	0.00541	2.8E-05	10	89714	ukb-d-22	|| id:u	TRUE	reported	igd	2	TRUE
rs772112	A	T	A	T	0.01135	-0.0198	0.86874	0.85224	FALSE	TRUE	FALSE	ebi-a-GC	17	33621127	0.0121	467253	0.1037	Gastroes	Gastroes	Gastroes	TRUE	igd	33621127	0.00265	1.8E-05	17	89714	ukb-d-22	|| id:u	TRUE	reported	igd	2	TRUE
rs773097	A	C	A	C	0.02921	0.0074	0.01555	0.02706	FALSE	FALSE	FALSE	ebi-a-GC	7	73663178	0.0285	467253	0.7945	Gastroes	Gastroes	Gastroes	TRUE	igd	73663178	0.00712	4.1E-05	7	89714	ukb-d-22	|| id:u	TRUE	reported	igd	2	TRUE
rs778055	T	G	T	G	0.01932	0.0018	0.05764	0.05902	FALSE	FALSE	FALSE	ebi-a-GC	6	144834312	0.018	467253	0.9201	Gastroes	Gastroes	Gastroes	TRUE	igd	144834312	0.0038	3.6E-07	6	89714	ukb-d-22	|| id:u	TRUE	reported	igd	2	TRUE
rs794986	G	A	G	A	-0.0277	0.0031	0.02296	0.01797	FALSE	FALSE	FALSE	ebi-a-GC	8	134855286	0.031	467253	0.9203	Gastroes	Gastroes	Gastroes	TRUE	igd	134855286	0.00583	2E-06	8	89714	ukb-d-22	|| id:u	TRUE	reported	igd	2	TRUE
rs795195	C	T	C	T	-0.0218	0.0063	0.02895	0.02473	FALSE	FALSE	FALSE	ebi-a-GC	14	34720312	0.0266	467253	0.8118	Gastroes	Gastroes	Gastroes	TRUE	igd	34720312	0.00521	2.9E-05	14	89714	ukb-d-22	|| id:u	TRUE	reported	igd	2	TRUE
rs797808	C	T	C	T	0.02474	0.0166	0.02269	0.01404	FALSE	FALSE	FALSE	ebi-a-GC	7	104247958	0.0303	467253	0.5836	Gastroes	Gastroes	Gastroes	TRUE	igd	104247958	0.00593	3E-05	7	89714	ukb-d-22	|| id:u	TRUE	reported	igd	2	TRUE
rs800655	C	G	C	G	0.01243	-0.0144	0.15964	0.23698	FALSE	TRUE	FALSE	ebi-a-GC	14	67294069	0.0111	467253	0.1963	Gastroes	Gastroes	Gastroes	TRUE	igd	67294069	0.00244	3.7E-07	14	89714	ukb-d-22	|| id:u	TRUE	reported	igd	2	TRUE
rs876462	G	T	G	T	-0.008	-0.0015	0.37434	0.42189	FALSE	FALSE	FALSE	ebi-a-GC	2	37519768	0.0084	467253	0.8605	Gastroes	Gastroes	Gastroes	TRUE	igd	37515880	0.00181	1E-05	2	89714	ukb-d-22	|| id:u	TRUE	reported	igd	2	TRUE
rs884258	C	T	C	T	-0.0079	-0.0014	0.67693	0.5456	FALSE	FALSE	FALSE	ebi-a-GC	9	107057177	0.0089	467253	0.8714	Gastroes	Gastroes	Gastroes	TRUE	igd	107057177	0.00189	3.3E-05	9	89714	ukb-d-22	|| id:u	TRUE	reported	igd	2	TRUE
rs914186	A	G	A	G	0.00773	0.0027	0.66006	0.70759	FALSE	FALSE	FALSE	ebi-a-GC	21	42619657	0.0088	467253	0.7621	Gastroes	Gastroes	Gastroes	TRUE	igd	42619657	0.00186	3.3E-05	21	89714	ukb-d-22	|| id:u	TRUE	reported	igd	2	TRUE
rs936835	T	A	T	A	0.03017	-0.0116	0.01474	0.14798	FALSE	TRUE	FALSE	ebi-a-GC	6	21808192	0.0262	467253	0.6573	Gastroes	Gastroes	Gastroes	TRUE	igd	21808192	0.00733	3.9E-05	6	89714	ukb-d-22	|| id:u	TRUE	reported	igd	2	TRUE
rs953033	G	T	G	T	0.0077	0.0017	0.65454	0.64472	FALSE	FALSE	FALSE	ebi-a-GC	13	75070201	0.0087	467253	0.8415	Gastroes	Gastroes	Gastroes	TRUE	igd	75070201	0.00185	3.1E-05	13	89714	ukb-d-22	|| id:u	TRUE	reported	igd	2	TRUE

Table S3. Detail Information of the Genetic Variants in the MR Analysis of SHS and IBS

SNP	effect a	other al	effect a	other al	beta.exp	beta.out	eaf.expo	eaf.outc	remove	palindro	ambiguou	id.outco	chr	pos	se.outco	samplesi	pval.out	outcome	original	outcome.	mr keep.	data sou	pos.exposu	se.expos	pval.exp	chr.expo	samplesi	id.expos	exposure	mr keep.	pval ori	data sou	action	mr keep
rs100090	A	G	A	G	-0.0076	0.0263	0.53599	0.5825	FALSE	FALSE	FALSE	finn-b-K	4	180336832	0.0219		0.2282	Irritabl	Irritabl	Irritabl	TRUE	igd	180336832	0.00176	1.4E-05	4	89714	ukb-d-22	|| id:u	TRUE	reported	igd	2	TRUE
rs102193	T	C	T	C	-0.0095	-0.0503	0.1902	0.1185	FALSE	FALSE	FALSE	finn-b-K	11	86339724	0.0332		0.13	Irritabl	Irritabl	Irritabl	TRUE	igd	86339724	0.00224	2.4E-05	11	89714	ukb-d-22	|| id:u	TRUE	reported	igd	2	TRUE
rs104255	C	T	C	T	-0.0084	0.0259	0.70783	0.7707	FALSE	FALSE	FALSE	finn-b-K	19	836043	0.0259		0.3162	Irritabl	Irritabl	Irritabl	TRUE	igd	836043	0.00204	3.6E-05	19	89714	ukb-d-22	|| id:u	TRUE	reported	igd	2	TRUE
rs109451	A	T	A	T	-0.0079	-0.0277	0.31369	0.2931	FALSE	TRUE	FALSE	finn-b-K	6	69354524	0.0238		0.2433	Irritabl	Irritabl	Irritabl	TRUE	igd	69354524	0.00189	3.2E-05	6	89714	ukb-d-22	|| id:u	TRUE	reported	igd	2	TRUE
rs112169	A	G	A	G	0.03019	0.0273	0.01694	0.01274	FALSE	FALSE	FALSE	finn-b-K	7	70641982	0.0972		0.779	Irritabl	Irritabl	Irritabl	TRUE	igd	70641982	0.00676	8.1E-06	7	89714	ukb-d-22	|| id:u	TRUE	reported	igd	2	TRUE
rs114134	T	C	T	C	0.04033	0.1782	0.00945	0.00255	FALSE	FALSE	FALSE	finn-b-K	6	41851676	0.2138		0.4047	Irritabl	Irritabl	Irritabl	TRUE	igd	41851676	0.00973	3.4E-05	6	89714	ukb-d-22	|| id:u	TRUE	reported	igd	2	TRUE
rs116249	T	C	T	C	-0.0289	0.0638	0.01801	0.01378	FALSE	FALSE	FALSE	finn-b-K	3	190482826	0.0916		0.4859	Irritabl	Irritabl	Irritabl	TRUE	igd	190482826	0.00697	3.5E-05	3	89714	ukb-d-22	|| id:u	TRUE	reported	igd	2	TRUE
rs117410	G	A	G	A	-0.0415	-0.2398	0.00888	0.00913	FALSE	FALSE	FALSE	finn-b-K	15	47979695	0.1124		0.0329	Irritabl	Irritabl	Irritabl	TRUE	igd	47979695	0.00945	1.1E-05	15	89714	ukb-d-22	|| id:u	TRUE	reported	igd	2	TRUE
rs117514	T	G	T	G	0.00909	-0.0275	0.18988	0.1775	FALSE	FALSE	FALSE	finn-b-K	22	49536215	0.0283		0.3308	Irritabl	Irritabl	Irritabl	TRUE	igd	49534383	0.00223	4.5E-05	22	89714	ukb-d-22	|| id:u	TRUE	reported	igd	2	TRUE
rs117858	A	G	A	G	-0.0212	0.0368	0.03627	0.04162	FALSE	FALSE	FALSE	finn-b-K	6	149289638	0.0545		0.4991	Irritabl	Irritabl	Irritabl	TRUE	igd	149289638	0.00484	1.2E-05	6	89714	ukb-d-22	|| id:u	TRUE	reported	igd	2	TRUE
rs118098	T	C	T	C	0.02983	0.0024	0.02017	0.0194	FALSE	FALSE	FALSE	finn-b-K	21	33944718	0.08		0.9763	Irritabl	Irritabl	Irritabl	TRUE	igd	33944718	0.00656	5.4E-06	21	89714	ukb-d-22	|| id:u	TRUE	reported	igd	2	TRUE
rs118146	G	A	G	A	-0.0131	0.0332	0.09386	0.09776	FALSE	FALSE	FALSE	finn-b-K	8	22116219	0.0368		0.3676	Irritabl	Irritabl	Irritabl	TRUE	igd	22116219	0.00312	2.5E-05	8	89714	ukb-d-22	|| id:u	TRUE	reported	igd	2	TRUE
rs120418	T	C	T	C	0.01394	0.0063	0.08023	0.0885	FALSE	FALSE	FALSE	finn-b-K	1	60484531	0.0381		0.8688	Irritabl	Irritabl	Irritabl	TRUE	igd	60484531	0.00324	1.7E-05	1	89714	ukb-d-22	|| id:u	TRUE	reported	igd	2	TRUE
rs121057	G	T	G	T	0.00874	0.002	0.36894	0.3297	FALSE	FALSE	FALSE	finn-b-K	2	60736985	0.0229		0.9308	Irritabl	Irritabl	Irritabl	TRUE	igd	60736985	0.00184	2E-06	2	89714	ukb-d-22	|| id:u	TRUE	reported	igd	2	TRUE
rs124091	C	T	C	T	-0.0085	0.0114	0.24869	0.2358	FALSE	FALSE	FALSE	finn-b-K	1	114055488	0.0254		0.6528	Irritabl	Irritabl	Irritabl	TRUE	igd	114055488	0.00208	3.9E-05	1	89714	ukb-d-22	|| id:u	TRUE	reported	igd	2	TRUE
rs124840	T	C	T	C	0.00905	-0.0099	0.20587	0.3167	FALSE	FALSE	FALSE	finn-b-K	22	51099862	0.0233		0.6704	Irritabl	Irritabl	Irritabl	TRUE	igd	51099862	0.00217	3E-05	22	89714	ukb-d-22	|| id:u	TRUE	reported	igd	2	TRUE
rs124932	G	T	G	T	0.01626	-0.1519	0.05371	0.02116	FALSE	FALSE	FALSE	finn-b-K	3	16679114	0.0736		0.03918	Irritabl	Irritabl	Irritabl	TRUE	igd	16679114	0.00388	2.8E-05	3	89714	ukb-d-22	|| id:u	TRUE	reported	igd	2	TRUE
rs126292	A	T	A	T	-0.0081	0.0158	0.33223	0.2444	FALSE	TRUE	FALSE	finn-b-K	6	16806082	0.0255		0.5361	Irritabl	Irritabl	Irritabl	TRUE	igd	16806082	0.00189	1.7E-05	6	89714	ukb-d-22	|| id:u	TRUE	reported	igd	2	TRUE
rs127653	T	C	T	C	0.00867	-0.0405	0.23241	0.2583	FALSE	FALSE	FALSE	finn-b-K	10	7631014	0.0249		0.1042	Irritabl	Irritabl	Irritabl	TRUE	igd	7631014	0.0021	3.7E-05	10	89714	ukb-d-22	|| id:u	TRUE	reported	igd	2	TRUE
rs128669	A	G	A	G	0.02524	-0.0757	0.02351	0.02955	FALSE	FALSE	FALSE	finn-b-K	13	110066103	0.0636		0.2337	Irritabl	Irritabl	Irritabl	TRUE	igd	110066103	0.00579	1.3E-05	13	89714	ukb-d-22	|| id:u	TRUE	reported	igd	2	TRUE
rs138858	A	G	A	G	0.04308	0.1187	0.00736	0.03539	FALSE	FALSE	FALSE	finn-b-K	15	79099986	0.0593		0.04539	Irritabl	Irritabl	Irritabl	TRUE	igd	79099986	0.01047	3.9E-05	15	89714	ukb-d-22	|| id:u	TRUE	reported	igd	2	TRUE
rs140647	G	A	G	A	0.04379	-0.0457	0.0076	0.01564	FALSE	FALSE	FALSE	finn-b-K	19	20290042	0.0881		0.6044	Irritabl	Irritabl	Irritabl	TRUE	igd	20290042	0.01059	3.6E-05	19	89714	ukb-d-22	|| id:u	TRUE	reported	igd	2	TRUE
rs142563	A	G	A	G	-0.0344	-0.0925	0.01473	0.02006	FALSE	FALSE	FALSE	finn-b-K	13	23278334	0.0788		0.2404	Irritabl	Irritabl	Irritabl	TRUE	igd	23278334	0.00745	4E-06	13	89714	ukb-d-22	|| id:u	TRUE	reported	igd	2	TRUE
rs143255	C	T	C	T	0.01694	-0.0504	0.04814	0.04673	FALSE	FALSE	FALSE	finn-b-K	7	147431844	0.0513		0.3262	Irritabl	Irritabl	Irritabl	TRUE	igd	147431844	0.00413	4.1E-05	7	89714	ukb-d-22	|| id:u	TRUE	reported	igd	2	TRUE
rs144479	A	G	A	G	0.05832	-0.1143	0.00476	0.0075	FALSE	FALSE	FALSE	finn-b-K	3	122728248	0.1259		0.3639	Irritabl	Irritabl	Irritabl	TRUE	igd	122728248	0.01297	6.9E-06	3	89714	ukb-d-22	|| id:u	TRUE	reported	igd	2	TRUE
rs145817	T	C	T	C	0.00807	0.0053	0.54956	0.569	FALSE	FALSE	FALSE	finn-b-K	12	41777144	0.0218		0.8083	Irritabl	Irritabl	Irritabl	TRUE	igd	41777144	0.00176	4.4E-06	12	89714	ukb-d-22	|| id:u	TRUE	reported	igd	2	TRUE
rs147567	A	G	A	G	0.01947	-0.0122	0.03983	0.00765	FALSE	FALSE	FALSE	finn-b-K	7	47544762	0.1263		0.9228	Irritabl	Irritabl	Irritabl	TRUE	igd	47544762	0.0047	3.5E-05	7	89714	ukb-d-22	|| id:u	TRUE	reported	igd	2	TRUE
rs148904	C	G	C	G	0.03265	0.0144	0.01511	0.00505	FALSE	TRUE	FALSE	finn-b-K	22	25249949	0.1521		0.9245	Irritabl	Irritabl	Irritabl	TRUE	igd	25249949	0.0076	1.7E-05	22	89714	ukb-d-22	|| id:u	TRUE	reported	igd	2	TRUE
rs149651	G	C	G	C	0.03813	-0.0379	0.01176	0.01384	FALSE	TRUE	FALSE	finn-b-K	6	160595479	0.0939		0.6862	Irritabl	Irritabl	Irritabl	TRUE	igd	160595479	0.00836	5E-06	6	89714	ukb-d-22	|| id:u	TRUE	reported	igd	2	TRUE
rs149772	T	A	T	A	0.0282	0.1251	0.02041	0.00869	FALSE	TRUE	FALSE	finn-b-K	4	12576228	0.1201		0.2975	Irritabl	Irritabl	Irritabl	TRUE	igd	12576228	0.00662	2E-05	4	89714	ukb-d-22	|| id:u	TRUE	reported	igd	2	TRUE
rs150100	C	T	C	T	0.02882	0.0327	0.02023	0.0126	FALSE	FALSE	FALSE	finn-b-K	3	96018959	0.0975		0.7373	Irritabl	Irritabl	Irritabl	TRUE	igd	96018959	0.00671	1.8E-05	3	89714	ukb-d-22	|| id:u	TRUE	reported	igd	2	TRUE
rs150689	A	G	A	G	0.03224	-0.2725	0.0145	0.00601	FALSE	FALSE	FALSE	finn-b-K	13	23446586	0.1424		0.05572	Irritabl	Irritabl	Irritabl	TRUE	igd	23446586	0.00765	2.5E-05	13	89714	ukb-d-22	|| id:u	TRUE	reported	igd	2	TRUE
rs171494	G	A	G	A	0.01389	-0.0105	0.08945	0.1262	FALSE	FALSE	FALSE	finn-b-K	2	235265207	0.0327		0.7485	Irritabl	Irritabl	Irritabl	TRUE	igd	235265207	0.00314	9.5E-06	2	89714	ukb-d-22	|| id:u	TRUE	reported	igd	2	TRUE
rs176026	A	G	A	G	0.03352	0.0206	0.01586	0.00817	FALSE	FALSE	FALSE	finn-b-K	12	42480604	0.1211		0.8651	Irritabl	Irritabl	Irritabl	TRUE	igd	42480604	0.00714	2.7E-06	12	89714	ukb-d-22	|| id:u	TRUE	reported	igd	2	TRUE
rs189879	T	G	T	G	0.02926	0.0175	0.01658	0.01577	FALSE	FALSE	FALSE	finn-b-K	4	69058096	0.087		0.8409	Irritabl	Irritabl	Irritabl	TRUE	igd	69058096	0.00705	3.3E-05	4	89714	ukb-d-22	|| id:u	TRUE	reported	igd	2	TRUE
rs192230	A	G	A	G	0.09947	-0.0416	0.00159	0.05087	FALSE	FALSE	FALSE	finn-b-K	19	50188478	0.0498		0.4039	Irritabl	Irritabl	Irritabl	TRUE	igd	50188478	0.02342	2.2E-05	19	89714	ukb-d-22	|| id:u	TRUE	reported	igd	2	TRUE
rs192463	T	A	T	A	0.02663	-0.0014	0.01955	0.00895	FALSE	TRUE	FALSE	finn-b-K	2	213744121	0.1171		0.9907	Irritabl	Irritabl	Irritabl	TRUE	igd	213744121	0.00644	3.6E-05	2	89714	ukb-d-22	|| id:u	TRUE	reported	igd	2	TRUE
rs199341	G	C	G	C	0.0083	0.0082	0.25339	0.2222	FALSE	TRUE	FALSE	finn-b-K	10	63400853	0.026		0.7538	Irritabl	Irritabl	Irritabl	TRUE	igd	63400853	0.00202	3.9E-05	10	89714	ukb-d-22	|| id:u	TRUE	reported	igd	2	TRUE
rs203146	C	T	C	T	0.00758	-0.0099	0.62391	0.7293	FALSE	FALSE	FALSE	finn-b-K	10	131923448	0.0243		0.6827	Irritabl	Irritabl	Irritabl	TRUE	igd	131923448	0.00181	2.9E-05	10	89714	ukb-d-22	|| id:u	TRUE	reported	igd	2	TRUE
rs206940	G	A	G	A	-0.0084	-0.015	0.34093	0.3135	FALSE	FALSE	FALSE	finn-b-K	12	56364321	0.0234		0.5208	Irritabl	Irritabl	Irritabl	TRUE	igd	56364321	0.00185	5.7E-06	12	89714	ukb-d-22	|| id:u	TRUE	reported	igd	2	TRUE
rs207008	G	T	G	T	-0.0196	0.0284	0.94188	0.9492	FALSE	FALSE	FALSE	finn-b-K	21	18735936	0.0493		0.5652	Irritabl	Irritabl	Irritabl	TRUE	igd	18735936	0.00375	1.8E-07	21	89714	ukb-d-22	|| id:u	TRUE	reported	igd	2	TRUE
rs229649	G	A	G	A	-0.0113	-0.0469	0.11402	0.1312	FALSE	FALSE	FALSE	finn-b-K	13	95096111	0.032		0.1424	Irritabl	Irritabl	Irritabl	TRUE	igd	95096111	0.00277	4.3E-05	13	89714	ukb-d-22	|| id:u	TRUE	reported	igd	2	TRUE
rs268312	C	A	C	A	-0.0072	-0.0167	0.46584	0.5135	FALSE	FALSE	FALSE	finn-b-K	15	36024804	0.0217		0.4425	Irritabl	Irritabl	Irritabl	TRUE	igd	36024804	0.00177	4.9E-05	15	89714	ukb-d-22	|| id:u	TRUE	reported	igd	2	TRUE
rs276583	A	G	A	G	0.01277	-0.0256	0.12036	0.08107	FALSE	FALSE	FALSE	finn-b-K	10	21034317	0.0397		0.5189	Irritabl	Irritabl	Irritabl	TRUE	igd	21034317	0.00272	2.7E-06	10	89714	ukb-d-22	|| id:u	TRUE	reported	igd	2	TRUE
rs284717	T	C	T	C	0.00882	0.019	0.63416	0.6129	FALSE	FALSE	FALSE	finn-b-K	11	105877624	0.0221		0.3914	Irritabl	Irritabl	Irritabl	TRUE	igd	105877624	0.00183	1.4E-06	11	89714	ukb-d-22	|| id:u	TRUE	reported	igd	2	TRUE
rs309354	A	G	A	G	-0.0185	-0.0127	0.04632	0.138	FALSE	FALSE	FALSE	finn-b-K	6	31539010	0.0463		0.7835	Irritabl	Irritabl	Irritabl	TRUE	igd	31539010	0.00418	9.8E-06	6	89714	ukb-d-22	|| id:u	TRUE	reported	igd	2	TRUE
rs310653	A	C	A	C	0.01211	-0.0285	0.10649	0.1599	FALSE	FALSE	FALSE	finn-b-K	8	131259140	0.0295		0.3351	Irritabl	Irritabl	Irritabl	TRUE	igd	131259140	0.00284	2E-05	8	89714	ukb-d-22	|| id:u	TRUE	reported	igd	2	TRUE
rs353857	T	A	T	A	0.00794	0.0004	0.63465	0.5758	FALSE	TRUE	TRUE	finn-b-K	3	196071832	0.0218		0.9855	Irritabl	Irritabl	Irritabl	TRUE	igd	196071832	0.00183	1.4E-05	3	89714	ukb-d-22	|| id:u	TRUE	reported	igd	2	FALSE
rs370870	A	G	A	G	0.01649	-0.0144	0.05074	0.1188	FALSE	FALSE	FALSE	finn-b-K	1	245981170	0.0334		0.666	Irritabl	Irritabl	Irritabl	TRUE	igd	245981170	0.00402	4.2E-05	1	89714	ukb-d-22	|| id:u	TRUE	reported	igd	2	TRUE
rs373969	A	G	A	G	0.00727	-0.0329	0.49641	0.5092	FALSE	FALSE	FALSE	finn-b-K	9	114798940	0.0217		0.1284	Irritabl	Irritabl	Irritabl	TRUE	igd	114798940	0.00177	3.9E-05	9	89714	ukb-d-22	|| id:u	TRUE	reported	igd	2	TRUE
rs382594	A	G	A	G	0.00829	-0.0165	0.25777	0.257	FALSE	FALSE	FALSE	finn-b-K	15	74317362	0.0247		0.5043	Irritabl	Irritabl	Irritabl	TRUE	igd	74317362	0.00202	3.9E-05	15	89714	ukb-d-22	|| id:u	TRUE	reported	igd	2	TRUE
rs389792	T	G	T	G	-0.0075	0.0192	0.431	0.2892	FALSE	FALSE	FALSE	finn-b-K	3	101703056	0.024		0.4236	Irritabl	Irritabl	Irritabl	TRUE	igd	101703056	0.00177	2.1E-05	3	89714	ukb-d-22	|| id:u	TRUE	reported	igd	2	TRUE
rs413194	T	G	T	G	-0.0074	-0.0059	0.51371	0.5512	FALSE	FALSE	FALSE	finn-b-K	16	54862861	0.0218		0.7861	Irritabl	Irritabl	Irritabl	TRUE	igd	54862861	0.00178	3.1E-05	16	89714	ukb-d-22	|| id:u	TRUE	reported	igd	2	TRUE
rs443395	T	A	T	A	0.0078	0.0223	0.43142	0.3894	FALSE	TRUE	TRUE	finn-b-K	2	238115406	0.0222		0.3137	Irritabl	Irritabl	Irritabl	TRUE	igd	238114802	0.00182	1.7E-05	2	89714	ukb-d-22	|| id:u	TRUE	reported	igd	2	FALSE
rs468829	T	C	T	C	0.01399	0.0335	0.07414	0.04703	FALSE	FALSE	FALSE	finn-b-K	3	62460883	0.0511		0.5113	Irritabl	Irritabl	Irritabl	TRUE	igd	62460883	0.00341	4E-05	3	89714	ukb-d-22	|| id:u	TRUE	reported	igd	2	TRUE
rs481445	C	T	C	T	-0.0168	0.0234	0.94596	0.9131	FALSE	FALSE	FALSE	finn-b-K	20	16178198	0.0384		0.5426	Irritabl	Irritabl	Irritabl	TRUE	igd	16178198	0.00387	1.5E-05	20	89714	ukb-d-22	|| id:u	TRUE	reported	igd	2	TRUE
rs497501	A	G	A	G	0.00903	0.0028	0.22088	0.2499	FALSE	FALSE	FALSE	finn-b-K	4	39423512	0.0249		0.9114	Irritabl	Irritabl	Irritabl	TRUE	igd	39423512	0.00213	2.2E-05	4	89714	ukb-d-22	|| id:u	TRUE	reported	igd	2	TRUE
rs513564	T	C	T	C	0.00846	-0.0215	0.24995	0.1782	FALSE	FALSE	FALSE	finn-b-K	10	99780908	0.0284		0.4494	Irritabl	Irritabl	Irritabl	TRUE	igd	99780908	0.00207	4.4E-05	10	89714	ukb-d-22	|| id:u	TRUE	reported	igd	2	TRUE
rs554912	C	G	C	G	-0.0124	0.0436	0.10323	0.1185	FALSE	TRUE	FALSE	finn-b-K	1	226737915	0.0334		0.1923	Irritabl	Irritabl	Irritabl	TRUE	igd	226735908	0.00295	2.8E-05	1	89714	ukb-d-22	|| id:u	TRUE	reported	igd	2	TRUE
rs558136	T	C	T	C	0.03104	0.0671	0.01488	0.01214	FALSE	FALSE	FALSE	finn-b-K	8	62318022	0.0986		0.4965	Irritabl	Irritabl	Irritabl	TRUE	igd	62318022	0.00726	1.9E-05	8	89714	ukb-d-22	|| id:u	TRUE	reported	igd	2	TRUE
rs559280	A	G	A	G	-0.026	-0.0352	0.02501	0.0158	FALSE	FALSE	FALSE	finn-b-K	16	69750910	0.0857		0.6811	Irritabl	Irritabl	Irritabl	TRUE	igd	69750910	0.00566	4.2E-06	16	89714	ukb-d-22	|| id:u	TRUE	reported	igd	2	TRUE
rs561694	T	C	T	C	-0.0141	0.1009	0.07356	0.05461	FALSE	FALSE	FALSE	finn-b-K	12	117155472	0.0485		0.0375	Irritabl	Irritabl	Irritabl	TRUE	igd	117155472	0.00347	4.9E-05	12	89714	ukb-d-22	|| id:u	TRUE	reported	igd	2	TRUE
rs576053	C	G	C	G	-0.0218	0.0023	0.02771	0.04108	FALSE	TRUE	FALSE	finn-b-K	6	151835770	0.0547		0.9667	Irritabl	Irritabl	Irritabl	TRUE	igd	151835770	0.00534	4.6E-05	6	89714	ukb-d-22	|| id:u	TRUE	reported	igd	2	TRUE
rs611254	T	C	T	C	-0.0151	0.0832	0.08847	0.07044	FALSE	FALSE	FALSE	finn-b-K	20	19727485	0.0422		0.04867	Irritabl	Irritabl	Irritabl	TRUE	igd	19727485	0.00308	8.6E-07	20	89714	ukb-d-22	|| id:u	TRUE	reported	igd	2	TRUE
rs613060	C	G	C	G	-0.0103	0.0426	0.19003	0.2574	FALSE	TRUE	FALSE	finn-b-K	20	43033095	0.0248		0.08577	Irritabl	Irritabl	Irritabl	TRUE	igd	43033095	0.00223	3.8E-06	20	89714	ukb-d-22	|| id:u	TRUE	reported	igd	2	TRUE
rs620553	G	T	G	T	-0.014	0.0277	0.08724	0.06697	FALSE	FALSE	FALSE	finn-b-K	16	30187673	0.0436		0.5251	Irritabl	Irritabl	Irritabl	TRUE	igd	30187673	0.00321	1.4E-05	16	89714	ukb-d-22	|| id:u	TRUE	reported	igd	2	TRUE
rs622080	G	A	G	A	0.02549	-0.0602	0.02231	0.01688	FALSE	FALSE	FALSE	finn-b-K	20	62043227	0.0835		0.4711	Irritabl	Irritabl	Irritabl	TRUE	igd	62043227	0.00592	1.6E-05	20	89714	ukb-d-22	|| id:u	TRUE	reported	igd	2	TRUE
rs624773	T	A	T	A	0.02016	-0.046	0.04486	0.04379	FALSE	TRUE	FALSE	finn-b-K	7	114935274	0.053		0.3859	Irritabl	Irritabl	Irritabl	TRUE	igd	114935274	0.00426	2.2E-06	7	89714	ukb-d-22	|| id:u	TRUE	reported	igd	2	TRUE
rs624961	T	C	T	C	0.02838	0.0563	0.02176	0.00761	FALSE	FALSE	FALSE	finn-b-K	8	9288470	0.1253		0.6534	Irritabl	Irritabl	Irritabl	TRUE	igd	9288470	0.00632	7.2E-06	8	89714	ukb-d-22	|| id:u	TRUE	reported	igd	2	TRUE
rs688129	C	T	C	T	0.00745	-0.0169	0.58387	0.565	FALSE	FALSE	FALSE	finn-b-K	5	11147494	0.0217		0.4353	Irritabl	Irritabl	Irritabl	TRUE	igd	11147494	0.00177	2.7E-05	5	89714	ukb-d-22	|| id:u	TRUE	reported	igd	2	TRUE
rs689896	G	C	G	C	0.00762	-0.0218	0.63769	0.6843	FALSE	TRUE	FALSE	finn-b-K	18	74078911	0.0234		0.3511	Irritabl	Irritabl	Irritabl	TRUE	igd	74078911	0.00182	2.8E-05	18	89714	ukb-d-22	|| id:u	TRUE	reported	igd	2	TRUE
rs691490	C	A	C	A	0.01026	0.0204	0.14903	0.1592	FALSE	FALSE	FALSE	finn-b-K	6	139021524	0.0297		0.4922	Irritabl	Irritabl	Irritabl	TRUE	igd	139021524	0.00249	3.7E-05	6	89714	ukb-d-22	|| id:u	TRUE	reported	igd	2	TRUE
rs708246	A	G	A	G	-0.0085	0.0056	0.67891	0.5834	FALSE	FALSE	FALSE	finn-b-K	10	50486727	0.0219		0.7971	Irritabl	Irritabl	Irritabl	TRUE	igd	50486727	0.00188	6.2E-06	10	89714	ukb-d-22	|| id:u	TRUE	reported	igd	2	TRUE
rs720454	G	C	G	C	0.00922	0.0417	0.69183	0.5862	FALSE	TRUE	FALSE	finn-b-K	16	718514	0.0221		0.05857	Irritabl	Irritabl	Irritabl	TRUE	igd	718514	0.00197	2.8E-06	16	89714	ukb-d-22	|| id:u	TRUE	reported	igd	2	TRUE
rs726791	A	G	A	G	0.01238	0.0186	0.09252	0.189	FALSE	FALSE	FALSE	finn-b-K	1	66625369	0.0277		0.5024	Irritabl	Irritabl	Irritabl	TRUE	igd	66625369	0.00303	4.5E-05	1	89714	ukb-d-22	|| id:u	TRUE	reported	igd	2	TRUE
rs727260	G	C	G	C	-0.0115	0.0641	0.1251	0.1171	FALSE	TRUE	FALSE	finn-b-K	9	38072104	0.0335		0.05568	Irritabl	Irritabl	Irritabl	TRUE	igd	38072104	0.00267	1.5E-05	9	89714	ukb-d-22	|| id:u	TRUE	reported	igd	2	TRUE
rs727603	C	T	C	T	0.03144	-0.0966	0.01409	0.02595	FALSE	FALSE	FALSE	finn-b-K	15	84192374	0.068		0.1554	Irritabl	Irritabl	Irritabl	TRUE	igd	84192374	0.00758	3.3E-05	15	89714	ukb-d-22	|| id:u	TRUE	reported	igd	2	TRUE
rs728961	A	G	A	G	0.0118	-0.0549	0.1081	0.06908	FALSE	FALSE	FALSE	finn-b-K	11	5845481	0.0426		0.1974	Irritabl	Irritabl	Irritabl	TRUE	igd	5845481	0.00291	5E-05	11	89714	ukb-d-22	|| id:u	TRUE	reported	igd	2	TRUE
rs732141	C	A	C	A	-0.0088	0.0303	0.24763	0.2013	FALSE	FALSE	FALSE	finn-b-K	12	121476099	0.0268		0.2582	Irritabl	Irritabl	Irritabl	TRUE	igd	121476099	0.00203	1.7E-05	12	89714	ukb-d-22	|| id:u	TRUE	reported	igd	2	TRUE
rs733601	C	T	C	T	-0.0085	0.0134	0.25051	0.214	FALSE	FALSE	FALSE	finn-b-K	21	41403037	0.0264		0.6132	Irritabl	Irritabl	Irritabl	TRUE	igd	41403037	0.00203	3E-05	21	89714	ukb-d-22	|| id:u	TRUE	reported	igd	2	TRUE
rs733619	C	T	C	T	-0.0073	-0.0229	0.44239	0.4563	FALSE	FALSE	FALSE	finn-b-K	13	53914953	0.0216		0.2892	Irritabl	Irritabl	Irritabl	TRUE	igd	53911048	0.00178	4.3E-05	13	89714	ukb-d-22	|| id:u	TRUE	reported	igd	2	TRUE
rs744051	A	G	A	G	-0.0229	-0.1404	0.02571	0.03507	FALSE	FALSE	FALSE	finn-b-K	2	208711524	0.0591		0.01758	Irritabl	Irritabl	Irritabl	TRUE	igd	208711524	0.00563	5E-05	2	89714	ukb-d-22	|| id:u	TRUE	reported	igd	2	TRUE
rs746397	T	A	T	A	0.01254	-0.0425	0.08923	0.1184	FALSE	TRUE	FALSE	finn-b-K	2	144885367	0.0337		0.2067	Irritabl	Irritabl	Irritabl	TRUE	igd	144885367	0.00307	4.5E-05	2	89714	ukb-d-22	|| id:u	TRUE	reported	igd	2	TRUE
rs762178	G	A	G	A	0.04401	-0.0386	0.00963	0.01247	FALSE	FALSE	FALSE	finn-b-K	8	94600393	0.0969		0.6907	Irritabl	Irritabl	Irritabl	TRUE	igd	94600393	0.00898	9.5E-07	8	89714	ukb-d-22	|| id:u	TRUE	reported	igd	2	TRUE
rs768794	T	C	T	C	-0.0227	-0.0899	0.02696	0.0092	FALSE	FALSE	FALSE	finn-b-K	10	87990995	0.1135		0.4284	Irritabl	Irritabl	Irritabl	TRUE	igd	87990995	0.00541	2.8E-05	10	89714	ukb-d-22	|| id:u	TRUE	reported	igd	2	TRUE
rs772112	A	T	A	T	0.01135	0.0293	0.86874	0.8085	FALSE	TRUE	FALSE	finn-b-K	17	33621127	0.0277		0.2909	Irritabl	Irritabl	Irritabl	TRUE	igd	33621127	0.00265	1.8E-05	17	89714	ukb-d-22	|| id:u	TRUE	reported	igd	2	TRUE
rs773097	A	C	A	C	0.02921	-0.0606	0.01555	0.03937	FALSE	FALSE	FALSE	finn-b-K	7	73663178	0.0553		0.2737	Irritabl	Irritabl	Irritabl	TRUE	igd	73663178	0.00712	4.1E-05	7	89714	ukb-d-22	|| id:u	TRUE	reported	igd	2	TRUE
rs778055	T	G	T	G	0.01932	0.07	0.05764	0.06135	FALSE	FALSE	FALSE	finn-b-K	6	144834312	0.0452		0.1211	Irritabl	Irritabl	Irritabl	TRUE	igd	144834312	0.0038	3.6E-07	6	89714	ukb-d-22	|| id:u	TRUE	reported	igd	2	TRUE
rs794986	G	A	G	A	-0.0277	0.1579	0.02296	0.00414	FALSE	FALSE	FALSE	finn-b-K	8	134855286	0.1684		0.3484	Irritabl	Irritabl	Irritabl	TRUE	igd	134855286	0.00583	2E-06	8	89714	ukb-d-22	|| id:u	TRUE	reported	igd	2	TRUE
rs795195	C	T	C	T	-0.0218	0.0748	0.02895	0.01247	FALSE	FALSE	FALSE	finn-b-K	14	34720312	0.0986		0.448	Irritabl	Irritabl	Irritabl	TRUE	igd	34720312	0.00521	2.9E-05	14	89714	ukb-d-22	|| id:u	TRUE	reported	igd	2	TRUE
rs797808	C	T	C	T	0.02474	0.0699	0.02269	0.00833	FALSE	FALSE	FALSE	finn-b-K	7	104247958	0.1174		0.5519	Irritabl	Irritabl	Irritabl	TRUE	igd	104247958	0.00593	3E-05	7	89714	ukb-d-22	|| id:u	TRUE	reported	igd	2	TRUE
rs800655	C	G	C	G	0.01243	-0.0187	0.15964	0.1794	FALSE	TRUE	FALSE	finn-b-K	14	67294069	0.0281		0.506	Irritabl	Irritabl	Irritabl	TRUE	igd	67294069	0.00244	3.7E-07	14	89714	ukb-d-22	|| id:u	TRUE	reported	igd	2	TRUE
rs876462	G	T	G	T	-0.008	0.0161	0.37434	0.4567	FALSE	FALSE	FALSE	finn-b-K	2	37515880	0.0216		0.4561	Irritabl	Irritabl	Irritabl	TRUE	igd	37515880	0.00181	1E-05	2	89714	ukb-d-22	|| id:u	TRUE	reported	igd	2	TRUE
rs884258	C	T	C	T	-0.0079	-0.011	0.67693	0.6533	FALSE	FALSE	FALSE	finn-b-K	9	107057177	0.0226		0.628	Irritabl	Irritabl	Irritabl	TRUE	igd	107057177	0.00189	3.3E-05	9	89714	ukb-d-22	|| id:u	TRUE	reported	igd	2	TRUE
rs914186	A	G	A	G	0.00773	-0.0208	0.66006	0.6037	FALSE	FALSE	FALSE	finn-b-K	21	42619657	0.0221		0.3464	Irritabl	Irritabl	Irritabl	TRUE	igd	42619657	0.00186	3.3E-05	21	89714	ukb-d-22	|| id:u	TRUE	reported	igd	2	TRUE
rs936835	T	A	T	A	0.03017	-0.0196	0.01474	0.0371	FALSE	TRUE	FALSE	finn-b-K	6	21808192	0.0575		0.7327	Irritabl	Irritabl	Irritabl	TRUE	igd	21808192	0.00733	3.9E-05	6	89714	ukb-d-22	|| id:u	TRUE	reported	igd	2	TRUE
rs953033	G	T	G	T	0.0077	-0.0027	0.65454	0.6284	FALSE	FALSE	FALSE	finn-b-K	13	75070201	0.0223		0.9053	Irritabl	Irritabl	Irritabl	TRUE	igd	75070201	0.00185	3.1E-05	13	89714	ukb-d-22	|| id:u	TRUE	reported	igd	2	TRUE

Table S4. Detail Information of the Genetic Variants in the MR Analysis of SHS and Cholelithiasis

SNP	effect a	other al	effect a	other al	beta.exp	beta.out	eaf.expo	eaf.outc	remove	palindro	ambiguou	id.outco	chr	pos	se.outco	samplesi	pval.out	outcome	original	outcome.	mr keep.	data sou	pos.exposu	se.expos	pval.exp	chr.expo	samplesi	id.expos	exposure	mr keep.	pval ori	data sou	action	mr keep
rs100090	A	G	A	G	-0.0076	-0.017	0.53599	0.5822	FALSE	FALSE	FALSE	finn-b-K	4	180336832	0.0122		0.1612	Cholelit	Cholelit	Cholelit	TRUE	igd	180336832	0.00176	1.4E-05	4	89714	ukb-d-22	|| id:u	TRUE	reported	igd	2	TRUE
rs102193	T	C	T	C	-0.0095	0.014	0.1902	0.1185	FALSE	FALSE	FALSE	finn-b-K	11	86339724	0.0185		0.4473	Cholelit	Cholelit	Cholelit	TRUE	igd	86339724	0.00224	2.4E-05	11	89714	ukb-d-22	|| id:u	TRUE	reported	igd	2	TRUE
rs104255	C	T	C	T	-0.0084	-0.0203	0.70783	0.7711	FALSE	FALSE	FALSE	finn-b-K	19	836043	0.0145		0.1595	Cholelit	Cholelit	Cholelit	TRUE	igd	836043	0.00204	3.6E-05	19	89714	ukb-d-22	|| id:u	TRUE	reported	igd	2	TRUE
rs109451	A	T	A	T	-0.0079	-0.0059	0.31369	0.2934	FALSE	TRUE	FALSE	finn-b-K	6	69354524	0.0132		0.6564	Cholelit	Cholelit	Cholelit	TRUE	igd	69354524	0.00189	3.2E-05	6	89714	ukb-d-22	|| id:u	TRUE	reported	igd	2	TRUE
rs112169	A	G	A	G	0.03019	0.0324	0.01694	0.01276	FALSE	FALSE	FALSE	finn-b-K	7	70641982	0.0544		0.5515	Cholelit	Cholelit	Cholelit	TRUE	igd	70641982	0.00676	8.1E-06	7	89714	ukb-d-22	|| id:u	TRUE	reported	igd	2	TRUE
rs114134	T	C	T	C	0.04033	0.0237	0.00945	0.00257	FALSE	FALSE	FALSE	finn-b-K	6	41851676	0.1156		0.8379	Cholelit	Cholelit	Cholelit	TRUE	igd	41851676	0.00973	3.4E-05	6	89714	ukb-d-22	|| id:u	TRUE	reported	igd	2	TRUE
rs116249	T	C	T	C	-0.0289	-0.0724	0.01801	0.01378	FALSE	FALSE	FALSE	finn-b-K	3	190482826	0.0515		0.1597	Cholelit	Cholelit	Cholelit	TRUE	igd	190482826	0.00697	3.5E-05	3	89714	ukb-d-22	|| id:u	TRUE	reported	igd	2	TRUE
rs117410	G	A	G	A	-0.0415	-0.0325	0.00888	0.00923	FALSE	FALSE	FALSE	finn-b-K	15	47979695	0.0626		0.6035	Cholelit	Cholelit	Cholelit	TRUE	igd	47979695	0.00945	1.1E-05	15	89714	ukb-d-22	|| id:u	TRUE	reported	igd	2	TRUE
rs117514	T	G	T	G	0.00909	0.0089	0.18988	0.1774	FALSE	FALSE	FALSE	finn-b-K	22	49536215	0.0158		0.5722	Cholelit	Cholelit	Cholelit	TRUE	igd	49534383	0.00223	4.5E-05	22	89714	ukb-d-22	|| id:u	TRUE	reported	igd	2	TRUE
rs117858	A	G	A	G	-0.0212	-0.0011	0.03627	0.04169	FALSE	FALSE	FALSE	finn-b-K	6	149289638	0.0302		0.9706	Cholelit	Cholelit	Cholelit	TRUE	igd	149289638	0.00484	1.2E-05	6	89714	ukb-d-22	|| id:u	TRUE	reported	igd	2	TRUE
rs118098	T	C	T	C	0.02983	0.0447	0.02017	0.01939	FALSE	FALSE	FALSE	finn-b-K	21	33944718	0.0443		0.3128	Cholelit	Cholelit	Cholelit	TRUE	igd	33944718	0.00656	5.4E-06	21	89714	ukb-d-22	|| id:u	TRUE	reported	igd	2	TRUE
rs118146	G	A	G	A	-0.0131	0.0058	0.09386	0.09765	FALSE	FALSE	FALSE	finn-b-K	8	22116219	0.0205		0.7761	Cholelit	Cholelit	Cholelit	TRUE	igd	22116219	0.00312	2.5E-05	8	89714	ukb-d-22	|| id:u	TRUE	reported	igd	2	TRUE
rs120418	T	C	T	C	0.01394	0.0066	0.08023	0.08837	FALSE	FALSE	FALSE	finn-b-K	1	60484531	0.0212		0.7555	Cholelit	Cholelit	Cholelit	TRUE	igd	60484531	0.00324	1.7E-05	1	89714	ukb-d-22	|| id:u	TRUE	reported	igd	2	TRUE
rs121057	G	T	G	T	0.00874	-0.0007	0.36894	0.3293	FALSE	FALSE	FALSE	finn-b-K	2	60736985	0.0128		0.9544	Cholelit	Cholelit	Cholelit	TRUE	igd	60736985	0.00184	2E-06	2	89714	ukb-d-22	|| id:u	TRUE	reported	igd	2	TRUE
rs124091	C	T	C	T	-0.0085	0.0047	0.24869	0.236	FALSE	FALSE	FALSE	finn-b-K	1	114055488	0.0141		0.7407	Cholelit	Cholelit	Cholelit	TRUE	igd	114055488	0.00208	3.9E-05	1	89714	ukb-d-22	|| id:u	TRUE	reported	igd	2	TRUE
rs124840	T	C	T	C	0.00905	0.0106	0.20587	0.3165	FALSE	FALSE	FALSE	finn-b-K	22	51099862	0.0129		0.4114	Cholelit	Cholelit	Cholelit	TRUE	igd	51099862	0.00217	3E-05	22	89714	ukb-d-22	|| id:u	TRUE	reported	igd	2	TRUE
rs124932	G	T	G	T	0.01626	0.0417	0.05371	0.02126	FALSE	FALSE	FALSE	finn-b-K	3	16679114	0.041		0.3092	Cholelit	Cholelit	Cholelit	TRUE	igd	16679114	0.00388	2.8E-05	3	89714	ukb-d-22	|| id:u	TRUE	reported	igd	2	TRUE
rs126292	A	T	A	T	-0.0081	-0.0077	0.33223	0.2442	FALSE	TRUE	FALSE	finn-b-K	6	16806082	0.0142		0.5884	Cholelit	Cholelit	Cholelit	TRUE	igd	16806082	0.00189	1.7E-05	6	89714	ukb-d-22	|| id:u	TRUE	reported	igd	2	TRUE
rs127653	T	C	T	C	0.00867	-0.0021	0.23241	0.2586	FALSE	FALSE	FALSE	finn-b-K	10	7631014	0.0139		0.8816	Cholelit	Cholelit	Cholelit	TRUE	igd	7631014	0.0021	3.7E-05	10	89714	ukb-d-22	|| id:u	TRUE	reported	igd	2	TRUE
rs128669	A	G	A	G	0.02524	-0.0528	0.02351	0.02961	FALSE	FALSE	FALSE	finn-b-K	13	110066103	0.0354		0.1356	Cholelit	Cholelit	Cholelit	TRUE	igd	110066103	0.00579	1.3E-05	13	89714	ukb-d-22	|| id:u	TRUE	reported	igd	2	TRUE
rs138858	A	G	A	G	0.04308	0.0192	0.00736	0.03531	FALSE	FALSE	FALSE	finn-b-K	15	79099986	0.0329		0.5586	Cholelit	Cholelit	Cholelit	TRUE	igd	79099986	0.01047	3.9E-05	15	89714	ukb-d-22	|| id:u	TRUE	reported	igd	2	TRUE
rs140647	G	A	G	A	0.04379	0.0949	0.0076	0.0156	FALSE	FALSE	FALSE	finn-b-K	19	20290042	0.0491		0.05317	Cholelit	Cholelit	Cholelit	TRUE	igd	20290042	0.01059	3.6E-05	19	89714	ukb-d-22	|| id:u	TRUE	reported	igd	2	TRUE
rs142563	A	G	A	G	-0.0344	-0.0333	0.01473	0.01996	FALSE	FALSE	FALSE	finn-b-K	13	23278334	0.044		0.4493	Cholelit	Cholelit	Cholelit	TRUE	igd	23278334	0.00745	4E-06	13	89714	ukb-d-22	|| id:u	TRUE	reported	igd	2	TRUE
rs143255	C	T	C	T	0.01694	-0.0046	0.04814	0.04648	FALSE	FALSE	FALSE	finn-b-K	7	147431844	0.0287		0.8722	Cholelit	Cholelit	Cholelit	TRUE	igd	147431844	0.00413	4.1E-05	7	89714	ukb-d-22	|| id:u	TRUE	reported	igd	2	TRUE
rs144479	A	G	A	G	0.05832	-0.0095	0.00476	0.00749	FALSE	FALSE	FALSE	finn-b-K	3	122728248	0.0704		0.8925	Cholelit	Cholelit	Cholelit	TRUE	igd	122728248	0.01297	6.9E-06	3	89714	ukb-d-22	|| id:u	TRUE	reported	igd	2	TRUE
rs145817	T	C	T	C	0.00807	-0.0193	0.54956	0.5687	FALSE	FALSE	FALSE	finn-b-K	12	41777144	0.0121		0.1112	Cholelit	Cholelit	Cholelit	TRUE	igd	41777144	0.00176	4.4E-06	12	89714	ukb-d-22	|| id:u	TRUE	reported	igd	2	TRUE
rs147567	A	G	A	G	0.01947	-0.076	0.03983	0.00762	FALSE	FALSE	FALSE	finn-b-K	7	47544762	0.0704		0.2799	Cholelit	Cholelit	Cholelit	TRUE	igd	47544762	0.0047	3.5E-05	7	89714	ukb-d-22	|| id:u	TRUE	reported	igd	2	TRUE
rs148904	C	G	C	G	0.03265	0.0615	0.01511	0.00502	FALSE	TRUE	FALSE	finn-b-K	22	25249949	0.0849		0.4684	Cholelit	Cholelit	Cholelit	TRUE	igd	25249949	0.0076	1.7E-05	22	89714	ukb-d-22	|| id:u	TRUE	reported	igd	2	TRUE
rs149651	G	C	G	C	0.03813	0.0511	0.01176	0.01378	FALSE	TRUE	FALSE	finn-b-K	6	160595479	0.0524		0.3296	Cholelit	Cholelit	Cholelit	TRUE	igd	160595479	0.00836	5E-06	6	89714	ukb-d-22	|| id:u	TRUE	reported	igd	2	TRUE
rs149772	T	A	T	A	0.0282	0.0732	0.02041	0.00868	FALSE	TRUE	FALSE	finn-b-K	4	12576228	0.0669		0.2739	Cholelit	Cholelit	Cholelit	TRUE	igd	12576228	0.00662	2E-05	4	89714	ukb-d-22	|| id:u	TRUE	reported	igd	2	TRUE
rs150100	C	T	C	T	0.02882	0.0027	0.02023	0.01262	FALSE	FALSE	FALSE	finn-b-K	3	96018959	0.0541		0.9607	Cholelit	Cholelit	Cholelit	TRUE	igd	96018959	0.00671	1.8E-05	3	89714	ukb-d-22	|| id:u	TRUE	reported	igd	2	TRUE
rs150689	A	G	A	G	0.03224	-0.0424	0.0145	0.00591	FALSE	FALSE	FALSE	finn-b-K	13	23446586	0.0799		0.5962	Cholelit	Cholelit	Cholelit	TRUE	igd	23446586	0.00765	2.5E-05	13	89714	ukb-d-22	|| id:u	TRUE	reported	igd	2	TRUE
rs171494	G	A	G	A	0.01389	0.0051	0.08945	0.1262	FALSE	FALSE	FALSE	finn-b-K	2	235265207	0.0182		0.7782	Cholelit	Cholelit	Cholelit	TRUE	igd	235265207	0.00314	9.5E-06	2	89714	ukb-d-22	|| id:u	TRUE	reported	igd	2	TRUE
rs176026	A	G	A	G	0.03352	-0.1038	0.01586	0.0081	FALSE	FALSE	FALSE	finn-b-K	12	42480604	0.0672		0.1223	Cholelit	Cholelit	Cholelit	TRUE	igd	42480604	0.00714	2.7E-06	12	89714	ukb-d-22	|| id:u	TRUE	reported	igd	2	TRUE
rs189879	T	G	T	G	0.02926	-0.0574	0.01658	0.0157	FALSE	FALSE	FALSE	finn-b-K	4	69058096	0.0487		0.2387	Cholelit	Cholelit	Cholelit	TRUE	igd	69058096	0.00705	3.3E-05	4	89714	ukb-d-22	|| id:u	TRUE	reported	igd	2	TRUE
rs192230	A	G	A	G	0.09947	-0.0058	0.00159	0.05068	FALSE	FALSE	FALSE	finn-b-K	19	50188478	0.0277		0.8338	Cholelit	Cholelit	Cholelit	TRUE	igd	50188478	0.02342	2.2E-05	19	89714	ukb-d-22	|| id:u	TRUE	reported	igd	2	TRUE
rs192463	T	A	T	A	0.02663	0.0582	0.01955	0.00896	FALSE	TRUE	FALSE	finn-b-K	2	213744121	0.0648		0.3691	Cholelit	Cholelit	Cholelit	TRUE	igd	213744121	0.00644	3.6E-05	2	89714	ukb-d-22	|| id:u	TRUE	reported	igd	2	TRUE
rs199341	G	C	G	C	0.0083	-0.0248	0.25339	0.2227	FALSE	TRUE	FALSE	finn-b-K	10	63400853	0.0145		0.08685	Cholelit	Cholelit	Cholelit	TRUE	igd	63400853	0.00202	3.9E-05	10	89714	ukb-d-22	|| id:u	TRUE	reported	igd	2	TRUE
rs203146	C	T	C	T	0.00758	0.0293	0.62391	0.729	FALSE	FALSE	FALSE	finn-b-K	10	131923448	0.0135		0.0305	Cholelit	Cholelit	Cholelit	TRUE	igd	131923448	0.00181	2.9E-05	10	89714	ukb-d-22	|| id:u	TRUE	reported	igd	2	TRUE
rs206940	G	A	G	A	-0.0084	-0.0078	0.34093	0.3132	FALSE	FALSE	FALSE	finn-b-K	12	56364321	0.013		0.5481	Cholelit	Cholelit	Cholelit	TRUE	igd	56364321	0.00185	5.7E-06	12	89714	ukb-d-22	|| id:u	TRUE	reported	igd	2	TRUE
rs207008	G	T	G	T	-0.0196	-0.0681	0.94188	0.9492	FALSE	FALSE	FALSE	finn-b-K	21	18735936	0.0274		0.01293	Cholelit	Cholelit	Cholelit	TRUE	igd	18735936	0.00375	1.8E-07	21	89714	ukb-d-22	|| id:u	TRUE	reported	igd	2	TRUE
rs229649	G	A	G	A	-0.0113	-0.0107	0.11402	0.1312	FALSE	FALSE	FALSE	finn-b-K	13	95096111	0.0178		0.5482	Cholelit	Cholelit	Cholelit	TRUE	igd	95096111	0.00277	4.3E-05	13	89714	ukb-d-22	|| id:u	TRUE	reported	igd	2	TRUE
rs268312	C	A	C	A	-0.0072	0.0242	0.46584	0.5131	FALSE	FALSE	FALSE	finn-b-K	15	36024804	0.0121		0.04595	Cholelit	Cholelit	Cholelit	TRUE	igd	36024804	0.00177	4.9E-05	15	89714	ukb-d-22	|| id:u	TRUE	reported	igd	2	TRUE
rs276583	A	G	A	G	0.01277	-0.005	0.12036	0.08103	FALSE	FALSE	FALSE	finn-b-K	10	21034317	0.0222		0.8212	Cholelit	Cholelit	Cholelit	TRUE	igd	21034317	0.00272	2.7E-06	10	89714	ukb-d-22	|| id:u	TRUE	reported	igd	2	TRUE
rs284717	T	C	T	C	0.00882	0	0.63416	0.6131	FALSE	FALSE	FALSE	finn-b-K	11	105877624	0.0123		0.9973	Cholelit	Cholelit	Cholelit	TRUE	igd	105877624	0.00183	1.4E-06	11	89714	ukb-d-22	|| id:u	TRUE	reported	igd	2	TRUE
rs309354	A	G	A	G	-0.0185	-0.0008	0.04632	0.1381	FALSE	FALSE	FALSE	finn-b-K	6	31539010	0.0258		0.9747	Cholelit	Cholelit	Cholelit	TRUE	igd	31539010	0.00418	9.8E-06	6	89714	ukb-d-22	|| id:u	TRUE	reported	igd	2	TRUE
rs310653	A	C	A	C	0.01211	0.0162	0.10649	0.1599	FALSE	FALSE	FALSE	finn-b-K	8	131259140	0.0164		0.3247	Cholelit	Cholelit	Cholelit	TRUE	igd	131259140	0.00284	2E-05	8	89714	ukb-d-22	|| id:u	TRUE	reported	igd	2	TRUE
rs353857	T	A	T	A	0.00794	-0.0039	0.63465	0.5768	FALSE	TRUE	TRUE	finn-b-K	3	196071832	0.0122		0.7464	Cholelit	Cholelit	Cholelit	TRUE	igd	196071832	0.00183	1.4E-05	3	89714	ukb-d-22	|| id:u	TRUE	reported	igd	2	FALSE
rs370870	A	G	A	G	0.01649	-0.0163	0.05074	0.1186	FALSE	FALSE	FALSE	finn-b-K	1	245981170	0.0186		0.3819	Cholelit	Cholelit	Cholelit	TRUE	igd	245981170	0.00402	4.2E-05	1	89714	ukb-d-22	|| id:u	TRUE	reported	igd	2	TRUE
rs373969	A	G	A	G	0.00727	0.0074	0.49641	0.5091	FALSE	FALSE	FALSE	finn-b-K	9	114798940	0.0121		0.5399	Cholelit	Cholelit	Cholelit	TRUE	igd	114798940	0.00177	3.9E-05	9	89714	ukb-d-22	|| id:u	TRUE	reported	igd	2	TRUE
rs382594	A	G	A	G	0.00829	-0.0189	0.25777	0.257	FALSE	FALSE	FALSE	finn-b-K	15	74317362	0.0138		0.1698	Cholelit	Cholelit	Cholelit	TRUE	igd	74317362	0.00202	3.9E-05	15	89714	ukb-d-22	|| id:u	TRUE	reported	igd	2	TRUE
rs389792	T	G	T	G	-0.0075	-0.0037	0.431	0.2891	FALSE	FALSE	FALSE	finn-b-K	3	101703056	0.0133		0.782	Cholelit	Cholelit	Cholelit	TRUE	igd	101703056	0.00177	2.1E-05	3	89714	ukb-d-22	|| id:u	TRUE	reported	igd	2	TRUE
rs413194	T	G	T	G	-0.0074	0.012	0.51371	0.5512	FALSE	FALSE	FALSE	finn-b-K	16	54862861	0.0122		0.3234	Cholelit	Cholelit	Cholelit	TRUE	igd	54862861	0.00178	3.1E-05	16	89714	ukb-d-22	|| id:u	TRUE	reported	igd	2	TRUE
rs443395	T	A	T	A	0.0078	-0.0019	0.43142	0.3897	FALSE	TRUE	TRUE	finn-b-K	2	238115406	0.0123		0.8782	Cholelit	Cholelit	Cholelit	TRUE	igd	238114802	0.00182	1.7E-05	2	89714	ukb-d-22	|| id:u	TRUE	reported	igd	2	FALSE
rs468829	T	C	T	C	0.01399	-0.0498	0.07414	0.04704	FALSE	FALSE	FALSE	finn-b-K	3	62460883	0.0285		0.08026	Cholelit	Cholelit	Cholelit	TRUE	igd	62460883	0.00341	4E-05	3	89714	ukb-d-22	|| id:u	TRUE	reported	igd	2	TRUE
rs481445	C	T	C	T	-0.0168	0.0273	0.94596	0.9132	FALSE	FALSE	FALSE	finn-b-K	20	16178198	0.0214		0.2032	Cholelit	Cholelit	Cholelit	TRUE	igd	16178198	0.00387	1.5E-05	20	89714	ukb-d-22	|| id:u	TRUE	reported	igd	2	TRUE
rs497501	A	G	A	G	0.00903	-0.0379	0.22088	0.25	FALSE	FALSE	FALSE	finn-b-K	4	39423512	0.0139		0.00653	Cholelit	Cholelit	Cholelit	TRUE	igd	39423512	0.00213	2.2E-05	4	89714	ukb-d-22	|| id:u	TRUE	reported	igd	2	TRUE
rs513564	T	C	T	C	0.00846	-0.0272	0.24995	0.1784	FALSE	FALSE	FALSE	finn-b-K	10	99780908	0.0158		0.08617	Cholelit	Cholelit	Cholelit	TRUE	igd	99780908	0.00207	4.4E-05	10	89714	ukb-d-22	|| id:u	TRUE	reported	igd	2	TRUE
rs554912	C	G	C	G	-0.0124	-0.0111	0.10323	0.1183	FALSE	TRUE	FALSE	finn-b-K	1	226737915	0.0187		0.5518	Cholelit	Cholelit	Cholelit	TRUE	igd	226735908	0.00295	2.8E-05	1	89714	ukb-d-22	|| id:u	TRUE	reported	igd	2	TRUE
rs558136	T	C	T	C	0.03104	0.0635	0.01488	0.01208	FALSE	FALSE	FALSE	finn-b-K	8	62318022	0.0545		0.2441	Cholelit	Cholelit	Cholelit	TRUE	igd	62318022	0.00726	1.9E-05	8	89714	ukb-d-22	|| id:u	TRUE	reported	igd	2	TRUE
rs559280	A	G	A	G	-0.026	-0.0594	0.02501	0.01589	FALSE	FALSE	FALSE	finn-b-K	16	69750910	0.0476		0.2119	Cholelit	Cholelit	Cholelit	TRUE	igd	69750910	0.00566	4.2E-06	16	89714	ukb-d-22	|| id:u	TRUE	reported	igd	2	TRUE
rs561694	T	C	T	C	-0.0141	-0.0096	0.07356	0.05479	FALSE	FALSE	FALSE	finn-b-K	12	117155472	0.0269		0.722	Cholelit	Cholelit	Cholelit	TRUE	igd	117155472	0.00347	4.9E-05	12	89714	ukb-d-22	|| id:u	TRUE	reported	igd	2	TRUE
rs576053	C	G	C	G	-0.0218	-0.0225	0.02771	0.04105	FALSE	TRUE	FALSE	finn-b-K	6	151835770	0.0304		0.4583	Cholelit	Cholelit	Cholelit	TRUE	igd	151835770	0.00534	4.6E-05	6	89714	ukb-d-22	|| id:u	TRUE	reported	igd	2	TRUE
rs611254	T	C	T	C	-0.0151	0.0192	0.08847	0.07044	FALSE	FALSE	FALSE	finn-b-K	20	19727485	0.0234		0.4136	Cholelit	Cholelit	Cholelit	TRUE	igd	19727485	0.00308	8.6E-07	20	89714	ukb-d-22	|| id:u	TRUE	reported	igd	2	TRUE
rs613060	C	G	C	G	-0.0103	0.0639	0.19003	0.2575	FALSE	TRUE	FALSE	finn-b-K	20	43033095	0.0138		3.5E-06	Cholelit	Cholelit	Cholelit	TRUE	igd	43033095	0.00223	3.8E-06	20	89714	ukb-d-22	|| id:u	TRUE	reported	igd	2	TRUE
rs620553	G	T	G	T	-0.014	0.0034	0.08724	0.06734	FALSE	FALSE	FALSE	finn-b-K	16	30187673	0.0241		0.8873	Cholelit	Cholelit	Cholelit	TRUE	igd	30187673	0.00321	1.4E-05	16	89714	ukb-d-22	|| id:u	TRUE	reported	igd	2	TRUE
rs622080	G	A	G	A	0.02549	0.0438	0.02231	0.01692	FALSE	FALSE	FALSE	finn-b-K	20	62043227	0.0466		0.3476	Cholelit	Cholelit	Cholelit	TRUE	igd	62043227	0.00592	1.6E-05	20	89714	ukb-d-22	|| id:u	TRUE	reported	igd	2	TRUE
rs624773	T	A	T	A	0.02016	0.0122	0.04486	0.04387	FALSE	TRUE	FALSE	finn-b-K	7	114935274	0.0294		0.6795	Cholelit	Cholelit	Cholelit	TRUE	igd	114935274	0.00426	2.2E-06	7	89714	ukb-d-22	|| id:u	TRUE	reported	igd	2	TRUE
rs624961	T	C	T	C	0.02838	0.0622	0.02176	0.00767	FALSE	FALSE	FALSE	finn-b-K	8	9288470	0.0702		0.3758	Cholelit	Cholelit	Cholelit	TRUE	igd	9288470	0.00632	7.2E-06	8	89714	ukb-d-22	|| id:u	TRUE	reported	igd	2	TRUE
rs688129	C	T	C	T	0.00745	0.001	0.58387	0.5651	FALSE	FALSE	FALSE	finn-b-K	5	11147494	0.0121		0.9363	Cholelit	Cholelit	Cholelit	TRUE	igd	11147494	0.00177	2.7E-05	5	89714	ukb-d-22	|| id:u	TRUE	reported	igd	2	TRUE
rs689896	G	C	G	C	0.00762	0.0153	0.63769	0.6838	FALSE	TRUE	FALSE	finn-b-K	18	74078911	0.013		0.2408	Cholelit	Cholelit	Cholelit	TRUE	igd	74078911	0.00182	2.8E-05	18	89714	ukb-d-22	|| id:u	TRUE	reported	igd	2	TRUE
rs691490	C	A	C	A	0.01026	-0.0068	0.14903	0.1588	FALSE	FALSE	FALSE	finn-b-K	6	139021524	0.0166		0.6819	Cholelit	Cholelit	Cholelit	TRUE	igd	139021524	0.00249	3.7E-05	6	89714	ukb-d-22	|| id:u	TRUE	reported	igd	2	TRUE
rs708246	A	G	A	G	-0.0085	0.0021	0.67891	0.5832	FALSE	FALSE	FALSE	finn-b-K	10	50486727	0.0122		0.866	Cholelit	Cholelit	Cholelit	TRUE	igd	50486727	0.00188	6.2E-06	10	89714	ukb-d-22	|| id:u	TRUE	reported	igd	2	TRUE
rs720454	G	C	G	C	0.00922	0.005	0.69183	0.5859	FALSE	TRUE	FALSE	finn-b-K	16	718514	0.0123		0.6843	Cholelit	Cholelit	Cholelit	TRUE	igd	718514	0.00197	2.8E-06	16	89714	ukb-d-22	|| id:u	TRUE	reported	igd	2	TRUE
rs726791	A	G	A	G	0.01238	0.0111	0.09252	0.1886	FALSE	FALSE	FALSE	finn-b-K	1	66625369	0.0154		0.4718	Cholelit	Cholelit	Cholelit	TRUE	igd	66625369	0.00303	4.5E-05	1	89714	ukb-d-22	|| id:u	TRUE	reported	igd	2	TRUE
rs727260	G	C	G	C	-0.0115	0.0317	0.1251	0.1173	FALSE	TRUE	FALSE	finn-b-K	9	38072104	0.0186		0.08862	Cholelit	Cholelit	Cholelit	TRUE	igd	38072104	0.00267	1.5E-05	9	89714	ukb-d-22	|| id:u	TRUE	reported	igd	2	TRUE
rs727603	C	T	C	T	0.03144	-0.0278	0.01409	0.02606	FALSE	FALSE	FALSE	finn-b-K	15	84192374	0.0377		0.4614	Cholelit	Cholelit	Cholelit	TRUE	igd	84192374	0.00758	3.3E-05	15	89714	ukb-d-22	|| id:u	TRUE	reported	igd	2	TRUE
rs728961	A	G	A	G	0.0118	-0.0191	0.1081	0.06901	FALSE	FALSE	FALSE	finn-b-K	11	5845481	0.0238		0.4217	Cholelit	Cholelit	Cholelit	TRUE	igd	5845481	0.00291	5E-05	11	89714	ukb-d-22	|| id:u	TRUE	reported	igd	2	TRUE
rs732141	C	A	C	A	-0.0088	-0.0073	0.24763	0.2013	FALSE	FALSE	FALSE	finn-b-K	12	121476099	0.015		0.6271	Cholelit	Cholelit	Cholelit	TRUE	igd	121476099	0.00203	1.7E-05	12	89714	ukb-d-22	|| id:u	TRUE	reported	igd	2	TRUE
rs733601	C	T	C	T	-0.0085	-0.0028	0.25051	0.2141	FALSE	FALSE	FALSE	finn-b-K	21	41403037	0.0147		0.8511	Cholelit	Cholelit	Cholelit	TRUE	igd	41403037	0.00203	3E-05	21	89714	ukb-d-22	|| id:u	TRUE	reported	igd	2	TRUE
rs733619	C	T	C	T	-0.0073	-0.0129	0.44239	0.4565	FALSE	FALSE	FALSE	finn-b-K	13	53914953	0.012		0.281	Cholelit	Cholelit	Cholelit	TRUE	igd	53911048	0.00178	4.3E-05	13	89714	ukb-d-22	|| id:u	TRUE	reported	igd	2	TRUE
rs744051	A	G	A	G	-0.0229	-0.0682	0.02571	0.03494	FALSE	FALSE	FALSE	finn-b-K	2	208711524	0.0328		0.03777	Cholelit	Cholelit	Cholelit	TRUE	igd	208711524	0.00563	5E-05	2	89714	ukb-d-22	|| id:u	TRUE	reported	igd	2	TRUE
rs746397	T	A	T	A	0.01254	0.0032	0.08923	0.118	FALSE	TRUE	FALSE	finn-b-K	2	144885367	0.0187		0.8653	Cholelit	Cholelit	Cholelit	TRUE	igd	144885367	0.00307	4.5E-05	2	89714	ukb-d-22	|| id:u	TRUE	reported	igd	2	TRUE
rs762178	G	A	G	A	0.04401	-0.0124	0.00963	0.01251	FALSE	FALSE	FALSE	finn-b-K	8	94600393	0.0541		0.8187	Cholelit	Cholelit	Cholelit	TRUE	igd	94600393	0.00898	9.5E-07	8	89714	ukb-d-22	|| id:u	TRUE	reported	igd	2	TRUE
rs768794	T	C	T	C	-0.0227	0.0142	0.02696	0.00924	FALSE	FALSE	FALSE	finn-b-K	10	87990995	0.0632		0.8226	Cholelit	Cholelit	Cholelit	TRUE	igd	87990995	0.00541	2.8E-05	10	89714	ukb-d-22	|| id:u	TRUE	reported	igd	2	TRUE
rs772112	A	T	A	T	0.01135	-0.0009	0.86874	0.8082	FALSE	TRUE	FALSE	finn-b-K	17	33621127	0.0154		0.9527	Cholelit	Cholelit	Cholelit	TRUE	igd	33621127	0.00265	1.8E-05	17	89714	ukb-d-22	|| id:u	TRUE	reported	igd	2	TRUE
rs773097	A	C	A	C	0.02921	-0.0045	0.01555	0.03932	FALSE	FALSE	FALSE	finn-b-K	7	73663178	0.031		0.885	Cholelit	Cholelit	Cholelit	TRUE	igd	73663178	0.00712	4.1E-05	7	89714	ukb-d-22	|| id:u	TRUE	reported	igd	2	TRUE
rs778055	T	G	T	G	0.01932	-0.0293	0.05764	0.06134	FALSE	FALSE	FALSE	finn-b-K	6	144834312	0.0252		0.2439	Cholelit	Cholelit	Cholelit	TRUE	igd	144834312	0.0038	3.6E-07	6	89714	ukb-d-22	|| id:u	TRUE	reported	igd	2	TRUE
rs794986	G	A	G	A	-0.0277	-0.0724	0.02296	0.00414	FALSE	FALSE	FALSE	finn-b-K	8	134855286	0.0934		0.4381	Cholelit	Cholelit	Cholelit	TRUE	igd	134855286	0.00583	2E-06	8	89714	ukb-d-22	|| id:u	TRUE	reported	igd	2	TRUE
rs795195	C	T	C	T	-0.0218	0.0658	0.02895	0.01254	FALSE	FALSE	FALSE	finn-b-K	14	34720312	0.0545		0.2275	Cholelit	Cholelit	Cholelit	TRUE	igd	34720312	0.00521	2.9E-05	14	89714	ukb-d-22	|| id:u	TRUE	reported	igd	2	TRUE
rs797808	C	T	C	T	0.02474	0.0697	0.02269	0.00834	FALSE	FALSE	FALSE	finn-b-K	7	104247958	0.0653		0.286	Cholelit	Cholelit	Cholelit	TRUE	igd	104247958	0.00593	3E-05	7	89714	ukb-d-22	|| id:u	TRUE	reported	igd	2	TRUE
rs800655	C	G	C	G	0.01243	0.0142	0.15964	0.1792	FALSE	TRUE	FALSE	finn-b-K	14	67294069	0.0157		0.3658	Cholelit	Cholelit	Cholelit	TRUE	igd	67294069	0.00244	3.7E-07	14	89714	ukb-d-22	|| id:u	TRUE	reported	igd	2	TRUE
rs876462	G	T	G	T	-0.008	-0.0018	0.37434	0.4561	FALSE	FALSE	FALSE	finn-b-K	2	37515880	0.0121		0.883	Cholelit	Cholelit	Cholelit	TRUE	igd	37515880	0.00181	1E-05	2	89714	ukb-d-22	|| id:u	TRUE	reported	igd	2	TRUE
rs884258	C	T	C	T	-0.0079	-0.0131	0.67693	0.6534	FALSE	FALSE	FALSE	finn-b-K	9	107057177	0.0126		0.2988	Cholelit	Cholelit	Cholelit	TRUE	igd	107057177	0.00189	3.3E-05	9	89714	ukb-d-22	|| id:u	TRUE	reported	igd	2	TRUE
rs914186	A	G	A	G	0.00773	0.0079	0.66006	0.6036	FALSE	FALSE	FALSE	finn-b-K	21	42619657	0.0123		0.5183	Cholelit	Cholelit	Cholelit	TRUE	igd	42619657	0.00186	3.3E-05	21	89714	ukb-d-22	|| id:u	TRUE	reported	igd	2	TRUE
rs936835	T	A	T	A	0.03017	-0.0346	0.01474	0.03713	FALSE	TRUE	FALSE	finn-b-K	6	21808192	0.0319		0.2773	Cholelit	Cholelit	Cholelit	TRUE	igd	21808192	0.00733	3.9E-05	6	89714	ukb-d-22	|| id:u	TRUE	reported	igd	2	TRUE
rs953033	G	T	G	T	0.0077	-0.0013	0.65454	0.6283	FALSE	FALSE	FALSE	finn-b-K	13	75070201	0.0124		0.9165	Cholelit	Cholelit	Cholelit	TRUE	igd	75070201	0.00185	3.1E-05	13	89714	ukb-d-22	|| id:u	TRUE	reported	igd	2	TRUE

Table S5. Detail Information of the Genetic Variants in the MR Analysis of SHS and Acute pancreatitis

SNP	effect_a	other_al	effect_a	other_al	beta.ex	beta.out	eaf.expo	eaf.outc	remove	palindro	ambiguou	id.outco	chr	pos	se.outco	samplesi	pval.out	outcome	original	outcome.	mr_keep.	data_sou	pos.expos	se.exposure	pval.exposu	chr.expo	samplesi	id.expos	exposure	mr_keep.	pval_ori	data_sou	action	mr_keep
rs10009	A	G	A	G	-0.0076	-0.0713	0.53599	0.5826	FALSE	FALSE	FALSE	finn-b-K	4	180336832	0.0269		0.00797	Acute pa	Acute pa	Acute pa	TRUE	igd	180336832	0.00176045	1.41938E-05	4	89714	ukb-d-22	|| id:u	TRUE	reported	igd	2	TRUE
rs10219	T	C	T	C	-0.0095	0.0296	0.1902	0.1181	FALSE	FALSE	FALSE	finn-b-K	11	86339724	0.0413		0.4738	Acute pa	Acute pa	Acute pa	TRUE	igd	86339724	0.0022387	2.42003E-05	11	89714	ukb-d-22	|| id:u	TRUE	reported	igd	2	TRUE
rs10425	C	T	C	T	-0.0084	0.0003	0.70783	0.7715	FALSE	FALSE	FALSE	finn-b-K	19	836043	0.032		0.9931	Acute pa	Acute pa	Acute pa	TRUE	igd	836043	0.00204125	3.62335E-05	19	89714	ukb-d-22	|| id:u	TRUE	reported	igd	2	TRUE
rs109451	A	T	A	T	-0.0079	0.0176	0.31369	0.2935	FALSE	TRUE	FALSE	finn-b-K	6	69354524	0.0292		0.5466	Acute pa	Acute pa	Acute pa	TRUE	igd	69354524	0.00188914	3.18031E-05	6	89714	ukb-d-22	|| id:u	TRUE	reported	igd	2	TRUE
rs11216	A	G	A	G	0.03019	-0.0872	0.01694	0.01275	FALSE	FALSE	FALSE	finn-b-K	7	70641982	0.1188		0.4629	Acute pa	Acute pa	Acute pa	TRUE	igd	70641982	0.00676279	8.06566E-06	7	89714	ukb-d-22	|| id:u	TRUE	reported	igd	2	TRUE
rs11413	T	C	T	C	0.04033	-0.7088	0.00945	0.00251	FALSE	FALSE	FALSE	finn-b-K	6	41851676	0.2747		0.00988	Acute pa	Acute pa	Acute pa	TRUE	igd	41851676	0.00973382	3.42902E-05	6	89714	ukb-d-22	|| id:u	TRUE	reported	igd	2	TRUE
rs11624	T	C	T	C	-0.0289	0.0196	0.01801	0.01384	FALSE	FALSE	FALSE	finn-b-K	3	190482826	0.1154		0.8651	Acute pa	Acute pa	Acute pa	TRUE	igd	190482826	0.00697431	3.46186E-05	3	89714	ukb-d-22	|| id:u	TRUE	reported	igd	2	TRUE
rs11741	G	A	G	A	-0.0415	-0.044	0.00888	0.00921	FALSE	FALSE	FALSE	finn-b-K	15	47979695	0.1423		0.7572	Acute pa	Acute pa	Acute pa	TRUE	igd	47979695	0.00945298	1.11265E-05	15	89714	ukb-d-22	|| id:u	TRUE	reported	igd	2	TRUE
rs117514	T	G	T	G	0.00909	0.0032	0.18988	0.1773	FALSE	FALSE	FALSE	finn-b-K	22	49536215	0.0349		0.9266	Acute pa	Acute pa	Acute pa	TRUE	igd	49534383	0.00222923	0.000045413	22	89714	ukb-d-22	|| id:u	TRUE	reported	igd	2	TRUE
rs11785	A	G	A	G	-0.0212	0.0431	0.03627	0.04164	FALSE	FALSE	FALSE	finn-b-K	6	149289638	0.0673		0.5223	Acute pa	Acute pa	Acute pa	TRUE	igd	149289638	0.00484085	1.19127E-05	6	89714	ukb-d-22	|| id:u	TRUE	reported	igd	2	TRUE
rs11809	T	C	T	C	0.02983	-0.0005	0.02017	0.01932	FALSE	FALSE	FALSE	finn-b-K	21	33944718	0.098		0.9959	Acute pa	Acute pa	Acute pa	TRUE	igd	33944718	0.00655698	5.40256E-06	21	89714	ukb-d-22	|| id:u	TRUE	reported	igd	2	TRUE
rs11814	G	A	G	A	-0.0131	0.0008	0.09386	0.09756	FALSE	FALSE	FALSE	finn-b-K	8	22116219	0.0453		0.9861	Acute pa	Acute pa	Acute pa	TRUE	igd	22116219	0.00311966	0.000025083	8	89714	ukb-d-22	|| id:u	TRUE	reported	igd	2	TRUE
rs12041	T	C	T	C	0.01394	-0.1072	0.08023	0.08825	FALSE	FALSE	FALSE	finn-b-K	1	60484531	0.047		0.02251	Acute pa	Acute pa	Acute pa	TRUE	igd	60484531	0.00324288	1.72536E-05	1	89714	ukb-d-22	|| id:u	TRUE	reported	igd	2	TRUE
rs121057	G	T	G	T	0.00874	-0.0266	0.36894	0.3293	FALSE	FALSE	FALSE	finn-b-K	2	60736985	0.0282		0.3447	Acute pa	Acute pa	Acute pa	TRUE	igd	60736985	0.00184062	2.03545E-06	2	89714	ukb-d-22	|| id:u	TRUE	reported	igd	2	TRUE
rs124091	C	T	C	T	-0.0085	-0.0166	0.24869	0.2359	FALSE	FALSE	FALSE	finn-b-K	1	114055488	0.0312		0.5944	Acute pa	Acute pa	Acute pa	TRUE	igd	114055488	0.00207888	3.92826E-05	1	89714	ukb-d-22	|| id:u	TRUE	reported	igd	2	TRUE
rs12484	T	C	T	C	0.00905	0.0255	0.20587	0.3166	FALSE	FALSE	FALSE	finn-b-K	22	51099862	0.0286		0.3726	Acute pa	Acute pa	Acute pa	TRUE	igd	51099862	0.00216889	3.02378E-05	22	89714	ukb-d-22	|| id:u	TRUE	reported	igd	2	TRUE
rs12493	G	T	G	T	0.01626	0.0085	0.05371	0.02112	FALSE	FALSE	FALSE	finn-b-K	3	16679114	0.0927		0.9267	Acute pa	Acute pa	Acute pa	TRUE	igd	16679114	0.00388067	0.000027858	3	89714	ukb-d-22	|| id:u	TRUE	reported	igd	2	TRUE
rs12629	A	T	A	T	-0.0081	-0.0032	0.33223	0.2442	FALSE	TRUE	FALSE	finn-b-K	6	16806082	0.0315		0.92	Acute pa	Acute pa	Acute pa	TRUE	igd	16806082	0.00188608	0.000016911	6	89714	ukb-d-22	|| id:u	TRUE	reported	igd	2	TRUE
rs12765	T	C	T	C	0.00867	-0.0117	0.23241	0.2588	FALSE	FALSE	FALSE	finn-b-K	10	7631014	0.0305		0.7017	Acute pa	Acute pa	Acute pa	TRUE	igd	7631014	0.00210372	3.73431E-05	10	89714	ukb-d-22	|| id:u	TRUE	reported	igd	2	TRUE
rs12866	A	G	A	G	0.02524	-0.0319	0.02351	0.02972	FALSE	FALSE	FALSE	finn-b-K	13	110066103	0.0776		0.6804	Acute pa	Acute pa	Acute pa	TRUE	igd	110066103	0.00578864	1.30473E-05	13	89714	ukb-d-22	|| id:u	TRUE	reported	igd	2	TRUE
rs13885	A	G	A	G	0.04308	-0.0005	0.00736	0.03534	FALSE	FALSE	FALSE	finn-b-K	15	79099986	0.0715		0.9949	Acute pa	Acute pa	Acute pa	TRUE	igd	79099986	0.0104699	3.87722E-05	15	89714	ukb-d-22	|| id:u	TRUE	reported	igd	2	TRUE
rs14064	G	A	G	A	0.04379	0.0161	0.0076	0.01552	FALSE	FALSE	FALSE	finn-b-K	19	20290042	0.1082		0.8817	Acute pa	Acute pa	Acute pa	TRUE	igd	20290042	0.0105942	3.57199E-05	19	89714	ukb-d-22	|| id:u	TRUE	reported	igd	2	TRUE
rs14256	A	G	A	G	-0.0344	-0.1145	0.01473	0.02003	FALSE	FALSE	FALSE	finn-b-K	13	23278334	0.095		0.2284	Acute pa	Acute pa	Acute pa	TRUE	igd	23278334	0.00745485	3.97732E-06	13	89714	ukb-d-22	|| id:u	TRUE	reported	igd	2	TRUE
rs14325	C	T	C	T	0.01694	0.0061	0.04814	0.04658	FALSE	FALSE	FALSE	finn-b-K	7	147431844	0.0628		0.9223	Acute pa	Acute pa	Acute pa	TRUE	igd	147431844	0.00412782	4.07005E-05	7	89714	ukb-d-22	|| id:u	TRUE	reported	igd	2	TRUE
rs14447	A	G	A	G	0.05832	0.1877	0.00476	0.00752	FALSE	FALSE	FALSE	finn-b-K	3	122728248	0.1568		0.2312	Acute pa	Acute pa	Acute pa	TRUE	igd	122728248	0.0129703	6.92118E-06	3	89714	ukb-d-22	|| id:u	TRUE	reported	igd	2	TRUE
rs145817	T	C	T	C	0.00807	-0.0008	0.54956	0.5691	FALSE	FALSE	FALSE	finn-b-K	12	41777144	0.0268		0.9766	Acute pa	Acute pa	Acute pa	TRUE	igd	41777144	0.00175604	4.35843E-06	12	89714	ukb-d-22	|| id:u	TRUE	reported	igd	2	TRUE
rs147567	A	G	A	G	0.01947	-0.0867	0.03983	0.00763	FALSE	FALSE	FALSE	finn-b-K	7	47544762	0.1574		0.5816	Acute pa	Acute pa	Acute pa	TRUE	igd	47544762	0.00470387	3.48401E-05	7	89714	ukb-d-22	|| id:u	TRUE	reported	igd	2	TRUE
rs14890	C	G	C	G	0.03265	0.4834	0.01511	0.00502	FALSE	TRUE	FALSE	finn-b-K	22	25249949	0.1915		0.0116	Acute pa	Acute pa	Acute pa	TRUE	igd	25249949	0.00759825	0.000017378	22	89714	ukb-d-22	|| id:u	TRUE	reported	igd	2	TRUE
rs149651	G	C	G	C	0.03813	0.0592	0.01176	0.01377	FALSE	TRUE	FALSE	finn-b-K	6	160595479	0.1153		0.6075	Acute pa	Acute pa	Acute pa	TRUE	igd	160595479	0.00835709	5.0494E-06	6	89714	ukb-d-22	|| id:u	TRUE	reported	igd	2	TRUE
rs14977	T	A	T	A	0.0282	0.1864	0.02041	0.00865	FALSE	TRUE	FALSE	finn-b-K	4	12576228	0.1474		0.206	Acute pa	Acute pa	Acute pa	TRUE	igd	12576228	0.0066206	2.04607E-05	4	89714	ukb-d-22	|| id:u	TRUE	reported	igd	2	TRUE
rs15010	C	T	C	T	0.02882	-0.15	0.02023	0.01257	FALSE	FALSE	FALSE	finn-b-K	3	96018959	0.1204		0.213	Acute pa	Acute pa	Acute pa	TRUE	igd	96018959	0.00671373	1.76762E-05	3	89714	ukb-d-22	|| id:u	TRUE	reported	igd	2	TRUE
rs15068	A	G	A	G	0.03224	0.0462	0.0145	0.00593	FALSE	FALSE	FALSE	finn-b-K	13	23446586	0.1765		0.7933	Acute pa	Acute pa	Acute pa	TRUE	igd	23446586	0.00765178	0.0000252	13	89714	ukb-d-22	|| id:u	TRUE	reported	igd	2	TRUE
rs17149	G	A	G	A	0.01389	-0.0089	0.08945	0.1261	FALSE	FALSE	FALSE	finn-b-K	2	235265207	0.0401		0.8243	Acute pa	Acute pa	Acute pa	TRUE	igd	235265207	0.00313598	9.48506E-06	2	89714	ukb-d-22	|| id:u	TRUE	reported	igd	2	TRUE
rs17602	A	G	A	G	0.03352	-0.0223	0.01586	0.00815	FALSE	FALSE	FALSE	finn-b-K	12	42480604	0.1503		0.8818	Acute pa	Acute pa	Acute pa	TRUE	igd	42480604	0.00714406	2.70576E-06	12	89714	ukb-d-22	|| id:u	TRUE	reported	igd	2	TRUE
rs18987	T	G	T	G	0.02926	0.1526	0.01658	0.01579	FALSE	FALSE	FALSE	finn-b-K	4	69058096	0.1076		0.1561	Acute pa	Acute pa	Acute pa	TRUE	igd	69058096	0.00705273	3.34842E-05	4	89714	ukb-d-22	|| id:u	TRUE	reported	igd	2	TRUE
rs19223	A	G	A	G	0.09947	0.0191	0.00159	0.05076	FALSE	FALSE	FALSE	finn-b-K	19	50188478	0.0603		0.7518	Acute pa	Acute pa	Acute pa	TRUE	igd	50188478	0.0234151	2.15909E-05	19	89714	ukb-d-22	|| id:u	TRUE	reported	igd	2	TRUE
rs19246	T	A	T	A	0.02663	0.1422	0.01955	0.00895	FALSE	TRUE	FALSE	finn-b-K	2	213744121	0.1415		0.3148	Acute pa	Acute pa	Acute pa	TRUE	igd	213744121	0.00643949	3.55771E-05	2	89714	ukb-d-22	|| id:u	TRUE	reported	igd	2	TRUE
rs199341	G	C	G	C	0.0083	-0.0442	0.25339	0.223	FALSE	TRUE	FALSE	finn-b-K	10	63400853	0.032		0.1668	Acute pa	Acute pa	Acute pa	TRUE	igd	63400853	0.00201769	3.92401E-05	10	89714	ukb-d-22	|| id:u	TRUE	reported	igd	2	TRUE
rs20314	C	T	C	T	0.00758	0.0126	0.62391	0.7288	FALSE	FALSE	FALSE	finn-b-K	10	131923448	0.0299		0.6739	Acute pa	Acute pa	Acute pa	TRUE	igd	131923448	0.00181432	2.94938E-05	10	89714	ukb-d-22	|| id:u	TRUE	reported	igd	2	TRUE
rs20694	G	A	G	A	-0.0084	-0.0282	0.34093	0.3132	FALSE	FALSE	FALSE	finn-b-K	12	56364321	0.0288		0.3278	Acute pa	Acute pa	Acute pa	TRUE	igd	56364321	0.00184948	5.70033E-06	12	89714	ukb-d-22	|| id:u	TRUE	reported	igd	2	TRUE
rs20700	G	T	G	T	-0.0196	-0.0643	0.94188	0.9494	FALSE	FALSE	FALSE	finn-b-K	21	18735936	0.0609		0.2909	Acute pa	Acute pa	Acute pa	TRUE	igd	18735936	0.00375031	1.83942E-07	21	89714	ukb-d-22	|| id:u	TRUE	reported	igd	2	TRUE
rs22964	G	A	G	A	-0.0113	0.0143	0.11402	0.1314	FALSE	FALSE	FALSE	finn-b-K	13	95096111	0.0391		0.7151	Acute pa	Acute pa	Acute pa	TRUE	igd	95096111	0.00276525	4.27445E-05	13	89714	ukb-d-22	|| id:u	TRUE	reported	igd	2	TRUE
rs26831	C	A	C	A	-0.0072	0.0118	0.46584	0.5128	FALSE	FALSE	FALSE	finn-b-K	15	36024804	0.0267		0.66	Acute pa	Acute pa	Acute pa	TRUE	igd	36024804	0.00177068	4.89317E-05	15	89714	ukb-d-22	|| id:u	TRUE	reported	igd	2	TRUE
rs27658	A	G	A	G	0.01277	0.0156	0.12036	0.08103	FALSE	FALSE	FALSE	finn-b-K	10	21034317	0.0491		0.7504	Acute pa	Acute pa	Acute pa	TRUE	igd	21034317	0.00272239	2.73307E-06	10	89714	ukb-d-22	|| id:u	TRUE	reported	igd	2	TRUE
rs284717	T	C	T	C	0.00882	0.0034	0.63416	0.6131	FALSE	FALSE	FALSE	finn-b-K	11	105877624	0.0273		0.9021	Acute pa	Acute pa	Acute pa	TRUE	igd	105877624	0.00183024	1.44358E-06	11	89714	ukb-d-22	|| id:u	TRUE	reported	igd	2	TRUE
rs30935	A	G	A	G	-0.0185	-0.0564	0.04632	0.1383	FALSE	FALSE	FALSE	finn-b-K	6	31539010	0.057		0.3224	Acute pa	Acute pa	Acute pa	TRUE	igd	31539010	0.004175	9.76113E-06	6	89714	ukb-d-22	|| id:u	TRUE	reported	igd	2	TRUE
rs31065	A	C	A	C	0.01211	-0.0202	0.10649	0.1597	FALSE	FALSE	FALSE	finn-b-K	8	131259140	0.0364		0.5796	Acute pa	Acute pa	Acute pa	TRUE	igd	131259140	0.00284134	2.04202E-05	8	89714	ukb-d-22	|| id:u	TRUE	reported	igd	2	TRUE
rs353857	T	A	T	A	0.00794	0.0042	0.63465	0.5768	FALSE	TRUE	TRUE	finn-b-K	3	196071832	0.0268		0.8767	Acute pa	Acute pa	Acute pa	TRUE	igd	196071832	0.00182508	1.35719E-05	3	89714	ukb-d-22	|| id:u	TRUE	reported	igd	2	FALSE
rs37087	A	G	A	G	0.01649	0.0548	0.05074	0.1189	FALSE	FALSE	FALSE	finn-b-K	1	245981170	0.0408		0.1789	Acute pa	Acute pa	Acute pa	TRUE	igd	245981170	0.00402385	4.18929E-05	1	89714	ukb-d-22	|| id:u	TRUE	reported	igd	2	TRUE
rs37396	A	G	A	G	0.00727	-0.0262	0.49641	0.5089	FALSE	FALSE	FALSE	finn-b-K	9	114798940	0.0267		0.3252	Acute pa	Acute pa	Acute pa	TRUE	igd	114798940	0.00176661	3.86598E-05	9	89714	ukb-d-22	|| id:u	TRUE	reported	igd	2	TRUE
rs38259	A	G	A	G	0.00829	-0.0152	0.25777	0.2573	FALSE	FALSE	FALSE	finn-b-K	15	74317362	0.0304		0.6166	Acute pa	Acute pa	Acute pa	TRUE	igd	74317362	0.00201638	3.93115E-05	15	89714	ukb-d-22	|| id:u	TRUE	reported	igd	2	TRUE
rs38979	T	G	T	G	-0.0075	-0.0106	0.431	0.2888	FALSE	FALSE	FALSE	finn-b-K	3	101703056	0.0295		0.7202	Acute pa	Acute pa	Acute pa	TRUE	igd	101703056	0.00177005	2.07114E-05	3	89714	ukb-d-22	|| id:u	TRUE	reported	igd	2	TRUE
rs41319	T	G	T	G	-0.0074	0.0113	0.51371	0.551	FALSE	FALSE	FALSE	finn-b-K	16	54862861	0.0268		0.6742	Acute pa	Acute pa	Acute pa	TRUE	igd	54862861	0.00177911	3.14297E-05	16	89714	ukb-d-22	|| id:u	TRUE	reported	igd	2	TRUE
rs44339	T	A	T	A	0.0078	-0.0071	0.43142	0.3898	FALSE	TRUE	TRUE	finn-b-K	2	238115406	0.0273		0.7947	Acute pa	Acute pa	Acute pa	TRUE	igd	238114802	0.00181535	1.74241E-05	2	89714	ukb-d-22	|| id:u	TRUE	reported	igd	2	FALSE
rs46882	T	C	T	C	0.01399	0.0173	0.07414	0.04715	FALSE	FALSE	FALSE	finn-b-K	3	62460883	0.0629		0.7839	Acute pa	Acute pa	Acute pa	TRUE	igd	62460883	0.00340649	0.000040071	3	89714	ukb-d-22	|| id:u	TRUE	reported	igd	2	TRUE
rs48144	C	T	C	T	-0.0168	0.0268	0.94596	0.913	FALSE	FALSE	FALSE	finn-b-K	20	16178198	0.0471		0.5699	Acute pa	Acute pa	Acute pa	TRUE	igd	16178198	0.00387384	1.45945E-05	20	89714	ukb-d-22	|| id:u	TRUE	reported	igd	2	TRUE
rs497501	A	G	A	G	0.00903	-0.0278	0.22088	0.2506	FALSE	FALSE	FALSE	finn-b-K	4	39423512	0.0306		0.3631	Acute pa	Acute pa	Acute pa	TRUE	igd	39423512	0.00212907	2.24311E-05	4	89714	ukb-d-22	|| id:u	TRUE	reported	igd	2	TRUE
rs51356	T	C	T	C	0.00846	0.0345	0.24995	0.1786	FALSE	FALSE	FALSE	finn-b-K	10	99780908	0.0351		0.325	Acute pa	Acute pa	Acute pa	TRUE	igd	99780908	0.00207117	4.38874E-05	10	89714	ukb-d-22	|| id:u	TRUE	reported	igd	2	TRUE
rs55491	C	G	C	G	-0.0124	0.015	0.10323	0.1183	FALSE	TRUE	FALSE	finn-b-K	1	226737915	0.0413		0.7177	Acute pa	Acute pa	Acute pa	TRUE	igd	226735908	0.00294957	0.000027707	1	89714	ukb-d-22	|| id:u	TRUE	reported	igd	2	TRUE
rs55813	T	C	T	C	0.03104	-0.1135	0.01488	0.01198	FALSE	FALSE	FALSE	finn-b-K	8	62318022	0.1228		0.3553	Acute pa	Acute pa	Acute pa	TRUE	igd	62318022	0.00725634	1.89051E-05	8	89714	ukb-d-22	|| id:u	TRUE	reported	igd	2	TRUE
rs55928	A	G	A	G	-0.026	-0.1525	0.02501	0.01586	FALSE	FALSE	FALSE	finn-b-K	16	69750910	0.108		0.1579	Acute pa	Acute pa	Acute pa	TRUE	igd	69750910	0.00565598	4.24277E-06	16	89714	ukb-d-22	|| id:u	TRUE	reported	igd	2	TRUE
rs56169	T	C	T	C	-0.0141	-0.0185	0.07356	0.05479	FALSE	FALSE	FALSE	finn-b-K	12	117155472	0.0596		0.756	Acute pa	Acute pa	Acute pa	TRUE	igd	117155472	0.00347247	4.85702E-05	12	89714	ukb-d-22	|| id:u	TRUE	reported	igd	2	TRUE
rs57605	C	G	C	G	-0.0218	0.0114	0.02771	0.04114	FALSE	TRUE	FALSE	finn-b-K	6	151835770	0.0668		0.8644	Acute pa	Acute pa	Acute pa	TRUE	igd	151835770	0.0053386	4.55093E-05	6	89714	ukb-d-22	|| id:u	TRUE	reported	igd	2	TRUE
rs61125	T	C	T	C	-0.0151	-0.0703	0.08847	0.07027	FALSE	FALSE	FALSE	finn-b-K	20	19727485	0.0518		0.1742	Acute pa	Acute pa	Acute pa	TRUE	igd	19727485	0.00307712	8.61906E-07	20	89714	ukb-d-22	|| id:u	TRUE	reported	igd	2	TRUE
rs61306	C	G	C	G	-0.0103	0.0149	0.19003	0.2567	FALSE	TRUE	FALSE	finn-b-K	20	43033095	0.0304		0.625	Acute pa	Acute pa	Acute pa	TRUE	igd	43033095	0.00223292	3.82384E-06	20	89714	ukb-d-22	|| id:u	TRUE	reported	igd	2	TRUE
rs62055	G	T	G	T	-0.014	0.0712	0.08724	0.06729	FALSE	FALSE	FALSE	finn-b-K	16	30187673	0.0536		0.184	Acute pa	Acute pa	Acute pa	TRUE	igd	30187673	0.00321154	1.36019E-05	16	89714	ukb-d-22	|| id:u	TRUE	reported	igd	2	TRUE
rs62208	G	A	G	A	0.02549	0.0479	0.02231	0.01687	FALSE	FALSE	FALSE	finn-b-K	20	62043227	0.1038		0.6444	Acute pa	Acute pa	Acute pa	TRUE	igd	62043227	0.00591581	1.64116E-05	20	89714	ukb-d-22	|| id:u	TRUE	reported	igd	2	TRUE
rs62477	T	A	T	A	0.02016	0.0119	0.04486	0.04387	FALSE	TRUE	FALSE	finn-b-K	7	114935274	0.0645		0.8538	Acute pa	Acute pa	Acute pa	TRUE	igd	114935274	0.00425824	2.18857E-06	7	89714	ukb-d-22	|| id:u	TRUE	reported	igd	2	TRUE
rs624961	T	C	T	C	0.02838	-0.0036	0.02176	0.00763	FALSE	FALSE	FALSE	finn-b-K	8	9288470	0.1584		0.9818	Acute pa	Acute pa	Acute pa	TRUE	igd	9288470	0.00632286	7.18108E-06	8	89714	ukb-d-22	|| id:u	TRUE	reported	igd	2	TRUE
rs68812	C	T	C	T	0.00745	0.0013	0.58387	0.5651	FALSE	FALSE	FALSE	finn-b-K	5	11147494	0.0267		0.9623	Acute pa	Acute pa	Acute pa	TRUE	igd	11147494	0.00177421	0.000026603	5	89714	ukb-d-22	|| id:u	TRUE	reported	igd	2	TRUE
rs68989	G	C	G	C	0.00762	0.0365	0.63769	0.6837	FALSE	TRUE	FALSE	finn-b-K	18	74078911	0.0288		0.2047	Acute pa	Acute pa	Acute pa	TRUE	igd	74078911	0.00182037	2.84997E-05	18	89714	ukb-d-22	|| id:u	TRUE	reported	igd	2	TRUE
rs69149	C	A	C	A	0.01026	0.0407	0.14903	0.1591	FALSE	FALSE	FALSE	finn-b-K	6	139021524	0.0364		0.264	Acute pa	Acute pa	Acute pa	TRUE	igd	139021524	0.00248621	3.67122E-05	6	89714	ukb-d-22	|| id:u	TRUE	reported	igd	2	TRUE
rs70824	A	G	A	G	-0.0085	0.0276	0.67891	0.5831	FALSE	FALSE	FALSE	finn-b-K	10	50486727	0.0269		0.3057	Acute pa	Acute pa	Acute pa	TRUE	igd	50486727	0.00187987	6.22171E-06	10	89714	ukb-d-22	|| id:u	TRUE	reported	igd	2	TRUE
rs72045	G	C	G	C	0.00922	0.0507	0.69183	0.586	FALSE	TRUE	FALSE	finn-b-K	16	718514	0.0271		0.06116	Acute pa	Acute pa	Acute pa	TRUE	igd	718514	0.00196696	2.75626E-06	16	89714	ukb-d-22	|| id:u	TRUE	reported	igd	2	TRUE
rs726791	A	G	A	G	0.01238	0.0464	0.09252	0.1886	FALSE	FALSE	FALSE	finn-b-K	1	66625369	0.0339		0.1722	Acute pa	Acute pa	Acute pa	TRUE	igd	66625369	0.00303411	4.49801E-05	1	89714	ukb-d-22	|| id:u	TRUE	reported	igd	2	TRUE
rs72726	G	C	G	C	-0.0115	-0.0789	0.1251	0.117	FALSE	TRUE	FALSE	finn-b-K	9	38072104	0.0411		0.05486	Acute pa	Acute pa	Acute pa	TRUE	igd	38072104	0.0026655	1.48977E-05	9	89714	ukb-d-22	|| id:u	TRUE	reported	igd	2	TRUE
rs72760	C	T	C	T	0.03144	-0.0449	0.01409	0.02607	FALSE	FALSE	FALSE	finn-b-K	15	84192374	0.084		0.5931	Acute pa	Acute pa	Acute pa	TRUE	igd	84192374	0.00757947	3.34989E-05	15	89714	ukb-d-22	|| id:u	TRUE	reported	igd	2	TRUE
rs728961	A	G	A	G	0.0118	0.0148	0.1081	0.0691	FALSE	FALSE	FALSE	finn-b-K	11	5845481	0.0526		0.7787	Acute pa	Acute pa	Acute pa	TRUE	igd	5845481	0.00290823	4.99057E-05	11	89714	ukb-d-22	|| id:u	TRUE	reported	igd	2	TRUE
rs732141	C	A	C	A	-0.0088	-0.0148	0.24763	0.2011	FALSE	FALSE	FALSE	finn-b-K	12	121476099	0.0332		0.6565	Acute pa	Acute pa	Acute pa	TRUE	igd	121476099	0.00203417	1.68043E-05	12	89714	ukb-d-22	|| id:u	TRUE	reported	igd	2	TRUE
rs733601	C	T	C	T	-0.0085	-0.0213	0.25051	0.214	FALSE	FALSE	FALSE	finn-b-K	21	41403037	0.0325		0.5127	Acute pa	Acute pa	Acute pa	TRUE	igd	41403037	0.00203355	3.03326E-05	21	89714	ukb-d-22	|| id:u	TRUE	reported	igd	2	TRUE
rs73361	C	T	C	T	-0.0073	0.0248	0.44239	0.4568	FALSE	FALSE	FALSE	finn-b-K	13	53914953	0.0265		0.3506	Acute pa	Acute pa	Acute pa	TRUE	igd	53911048	0.00177597	4.26589E-05	13	89714	ukb-d-22	|| id:u	TRUE	reported	igd	2	TRUE
rs744051	A	G	A	G	-0.0229	0	0.02571	0.03511	FALSE	FALSE	FALSE	finn-b-K	2	208711524	0.0721		0.9996	Acute pa	Acute pa	Acute pa	TRUE	igd	208711524	0.00563455	4.99793E-05	2	89714	ukb-d-22	|| id:u	TRUE	reported	igd	2	TRUE
rs74639	T	A	T	A	0.01254	0.0729	0.08923	0.1181	FALSE	TRUE	FALSE	finn-b-K	2	144885367	0.0412		0.07655	Acute pa	Acute pa	Acute pa	TRUE	igd	144885367	0.00307039	4.45841E-05	2	89714	ukb-d-22	|| id:u	TRUE	reported	igd	2	TRUE
rs76217	G	A	G	A	0.04401	-0.0764	0.00963	0.0125	FALSE	FALSE	FALSE	finn-b-K	8	94600393	0.12		0.5242	Acute pa	Acute pa	Acute pa	TRUE	igd	94600393	0.00897803	9.52401E-07	8	89714	ukb-d-22	|| id:u	TRUE	reported	igd	2	TRUE
rs76879	T	C	T	C	-0.0227	0.0784	0.02696	0.00925	FALSE	FALSE	FALSE	finn-b-K	10	87990995	0.1398		0.5748	Acute pa	Acute pa	Acute pa	TRUE	igd	87990995	0.00540836	0.000027792	10	89714	ukb-d-22	|| id:u	TRUE	reported	igd	2	TRUE
rs77211	A	T	A	T	0.01135	0.0584	0.86874	0.8083	FALSE	TRUE	FALSE	finn-b-K	17	33621127	0.034		0.08615	Acute pa	Acute pa	Acute pa	TRUE	igd	33621127	0.00264721	1.82343E-05	17	89714	ukb-d-22	|| id:u	TRUE	reported	igd	2	TRUE
rs77309	A	C	A	C	0.02921	-0.0679	0.01555	0.03935	FALSE	FALSE	FALSE	finn-b-K	7	73663178	0.0677		0.3159	Acute pa	Acute pa	Acute pa	TRUE	igd	73663178	0.00711909	4.07549E-05	7	89714	ukb-d-22	|| id:u	TRUE	reported	igd	2	TRUE
rs77805	T	G	T	G	0.01932	0.0448	0.05764	0.06153	FALSE	FALSE	FALSE	finn-b-K	6	144834312	0.0554		0.4189	Acute pa	Acute pa	Acute pa	TRUE	igd	144834312	0.00379766	3.64309E-07	6	89714	ukb-d-22	|| id:u	TRUE	reported	igd	2	TRUE
rs79498	G	A	G	A	-0.0277	-0.0393	0.02296	0.00416	FALSE	FALSE	FALSE	finn-b-K	8	134855286	0.2069		0.8492	Acute pa	Acute pa	Acute pa	TRUE	igd	134855286	0.00583247	2.01094E-06	8	89714	ukb-d-22	|| id:u	TRUE	reported	igd	2	TRUE
rs79519	C	T	C	T	-0.0218	0.1008	0.02895	0.01246	FALSE	FALSE	FALSE	finn-b-K	14	34720312	0.1234		0.4141	Acute pa	Acute pa	Acute pa	TRUE	igd	34720312	0.00520882	2.88051E-05	14	89714	ukb-d-22	|| id:u	TRUE	reported	igd	2	TRUE
rs79780	C	T	C	T	0.02474	0.1447	0.02269	0.00829	FALSE	FALSE	FALSE	finn-b-K	7	104247958	0.1452		0.319	Acute pa	Acute pa	Acute pa	TRUE	igd	104247958	0.0059315	3.03319E-05	7	89714	ukb-d-22	|| id:u	TRUE	reported	igd	2	TRUE
rs80065	C	G	C	G	0.01243	-0.0356	0.15964	0.1789	FALSE	TRUE	FALSE	finn-b-K	14	67294069	0.0347		0.3043	Acute pa	Acute pa	Acute pa	TRUE	igd	67294069	0.00244429	3.67333E-07	14	89714	ukb-d-22	|| id:u	TRUE	reported	igd	2	TRUE
rs87646	G	T	G	T	-0.008	0.0486	0.37434	0.4564	FALSE	FALSE	FALSE	finn-b-K	2	37515880	0.0266		0.06799	Acute pa	Acute pa	Acute pa	TRUE	igd	37515880	0.00181056	9.95749E-06	2	89714	ukb-d-22	|| id:u	TRUE	reported	igd	2	TRUE
rs88425	C	T	C	T	-0.0079	-0.038	0.67693	0.6534	FALSE	FALSE	FALSE	finn-b-K	9	107057177	0.0279		0.1721	Acute pa	Acute pa	Acute pa	TRUE	igd	107057177	0.00189426	3.33841E-05	9	89714	ukb-d-22	|| id:u	TRUE	reported	igd	2	TRUE
rs91418	A	G	A	G	0.00773	0.0002	0.66006	0.6034	FALSE	FALSE	FALSE	finn-b-K	21	42619657	0.0271		0.9939	Acute pa	Acute pa	Acute pa	TRUE	igd	42619657	0.00186074	0.000032892	21	89714	ukb-d-22	|| id:u	TRUE	reported	igd	2	TRUE
rs93683	T	A	T	A	0.03017	0.0926	0.01474	0.03726	FALSE	TRUE	FALSE	finn-b-K	6	21808192	0.0699		0.1852	Acute pa	Acute pa	Acute pa	TRUE	igd	21808192	0.00733453	3.89314E-05	6	89714	ukb-d-22	|| id:u	TRUE	reported	igd	2	TRUE
rs95303	G	T	G	T	0.0077	-0.0053	0.65454	0.6283	FALSE	FALSE	FALSE	finn-b-K	13	75070201	0.0275		0.8458	Acute pa	Acute pa	Acute pa	TRUE	igd	75070201	0.00184866	3.14434E-05	13	89714	ukb-d-22	|| id:u	TRUE	reported	igd	2	TRUE

Table S6. Detail Information of the Genetic Variants in the MR Analysis of SHS and Chronic pancreatitis

SNP	effect a	other al	effect a	other al	beta.exp	beta.out	eaf.expo	eaf.outc	remove	palindro	ambiguou	id.outco	chr	pos	se.outco	samplesi	pval.out	outcome	original	outcome.	mr_keep.	data_sou	pos.expo	se.expos	pval.exp	chr.expo	samplesi	id.expos	exposure	mr_keep.	pval_ori	data_sou	action	mr_keep
rs100090	A	G	A	G	-0.0076	-0.0671	0.53599	0.5827	FALSE	FALSE	FALSE	finn-b-K	4	18033683	0.0353		0.05722	Chronic	Chronic	Chronic	TRUE	igd	1.8E+08	0.00176	1.4E-05	4	89714	ukb-d-22	|| id:u	TRUE	reported	igd	2	TRUE
rs102193	T	C	T	C	-0.0095	0.017	0.1902	0.1181	FALSE	FALSE	FALSE	finn-b-K	11	86339724	0.0544		0.7547	Chronic	Chronic	Chronic	TRUE	igd	8.6E+07	0.00224	2.4E-05	11	89714	ukb-d-22	|| id:u	TRUE	reported	igd	2	TRUE
rs104255	C	T	C	T	-0.0084	-0.017	0.70783	0.7715	FALSE	FALSE	FALSE	finn-b-K	19	836043	0.042		0.6863	Chronic	Chronic	Chronic	TRUE	igd	836043	0.00204	3.6E-05	19	89714	ukb-d-22	|| id:u	TRUE	reported	igd	2	TRUE
rs109451	A	T	A	T	-0.0079	0.0071	0.31369	0.2935	FALSE	TRUE	FALSE	finn-b-K	6	69354524	0.0384		0.8542	Chronic	Chronic	Chronic	TRUE	igd	6.9E+07	0.00189	3.2E-05	6	89714	ukb-d-22	|| id:u	TRUE	reported	igd	2	TRUE
rs112169	A	G	A	G	0.03019	-0.0909	0.01694	0.01276	FALSE	FALSE	FALSE	finn-b-K	7	70641982	0.155		0.5579	Chronic	Chronic	Chronic	TRUE	igd	7.1E+07	0.00676	8.1E-06	7	89714	ukb-d-22	|| id:u	TRUE	reported	igd	2	TRUE
rs114134	T	C	T	C	0.04033	-0.3803	0.00945	0.00253	FALSE	FALSE	FALSE	finn-b-K	6	41851676	0.3615		0.2929	Chronic	Chronic	Chronic	TRUE	igd	4.2E+07	0.00973	3.4E-05	6	89714	ukb-d-22	|| id:u	TRUE	reported	igd	2	TRUE
rs116249	T	C	T	C	-0.0289	-0.0394	0.01801	0.01383	FALSE	FALSE	FALSE	finn-b-K	3	19048282	0.153		0.7966	Chronic	Chronic	Chronic	TRUE	igd	1.9E+08	0.00697	3.5E-05	3	89714	ukb-d-22	|| id:u	TRUE	reported	igd	2	TRUE
rs117410	G	A	G	A	-0.0415	0.0427	0.00888	0.00922	FALSE	FALSE	FALSE	finn-b-K	15	47979695	0.1897		0.822	Chronic	Chronic	Chronic	TRUE	igd	4.8E+07	0.00945	1.1E-05	15	89714	ukb-d-22	|| id:u	TRUE	reported	igd	2	TRUE
rs117514	T	G	T	G	0.00909	-0.0625	0.18988	0.1772	FALSE	FALSE	FALSE	finn-b-K	22	49536215	0.0458		0.1722	Chronic	Chronic	Chronic	TRUE	igd	5E+07	0.00223	4.5E-05	22	89714	ukb-d-22	|| id:u	TRUE	reported	igd	2	TRUE
rs117858	A	G	A	G	-0.0212	-0.0152	0.03627	0.04161	FALSE	FALSE	FALSE	finn-b-K	6	14928963	0.0886		0.8641	Chronic	Chronic	Chronic	TRUE	igd	1.5E+08	0.00484	1.2E-05	6	89714	ukb-d-22	|| id:u	TRUE	reported	igd	2	TRUE
rs118098	T	C	T	C	0.02983	-0.0337	0.02017	0.01932	FALSE	FALSE	FALSE	finn-b-K	21	33944718	0.1291		0.7941	Chronic	Chronic	Chronic	TRUE	igd	3.4E+07	0.00656	5.4E-06	21	89714	ukb-d-22	|| id:u	TRUE	reported	igd	2	TRUE
rs118146	G	A	G	A	-0.0131	-0.0044	0.09386	0.09756	FALSE	FALSE	FALSE	finn-b-K	8	22116219	0.0595		0.9413	Chronic	Chronic	Chronic	TRUE	igd	2.2E+07	0.00312	2.5E-05	8	89714	ukb-d-22	|| id:u	TRUE	reported	igd	2	TRUE
rs120418	T	C	T	C	0.01394	-0.0393	0.08023	0.08836	FALSE	FALSE	FALSE	finn-b-K	1	60484531	0.0615		0.5225	Chronic	Chronic	Chronic	TRUE	igd	6E+07	0.00324	1.7E-05	1	89714	ukb-d-22	|| id:u	TRUE	reported	igd	2	TRUE
rs121057	G	T	G	T	0.00874	-0.0002	0.36894	0.3294	FALSE	FALSE	FALSE	finn-b-K	2	60736985	0.037		0.9951	Chronic	Chronic	Chronic	TRUE	igd	6.1E+07	0.00184	2E-06	2	89714	ukb-d-22	|| id:u	TRUE	reported	igd	2	TRUE
rs124091	C	T	C	T	-0.0085	-0.0044	0.24869	0.2359	FALSE	FALSE	FALSE	finn-b-K	1	11405548	0.041		0.9149	Chronic	Chronic	Chronic	TRUE	igd	1.1E+08	0.00208	3.9E-05	1	89714	ukb-d-22	|| id:u	TRUE	reported	igd	2	TRUE
rs124840	T	C	T	C	0.00905	0.056	0.20587	0.3167	FALSE	FALSE	FALSE	finn-b-K	22	51099862	0.0374		0.1346	Chronic	Chronic	Chronic	TRUE	igd	5.1E+07	0.00217	3E-05	22	89714	ukb-d-22	|| id:u	TRUE	reported	igd	2	TRUE
rs124932	G	T	G	T	0.01626	0.2088	0.05371	0.02115	FALSE	FALSE	FALSE	finn-b-K	3	16679114	0.123		0.08977	Chronic	Chronic	Chronic	TRUE	igd	1.7E+07	0.00388	2.8E-05	3	89714	ukb-d-22	|| id:u	TRUE	reported	igd	2	TRUE
rs126292	A	T	A	T	-0.0081	-0.0044	0.33223	0.2442	FALSE	TRUE	FALSE	finn-b-K	6	16806082	0.0413		0.9146	Chronic	Chronic	Chronic	TRUE	igd	1.7E+07	0.00189	1.7E-05	6	89714	ukb-d-22	|| id:u	TRUE	reported	igd	2	TRUE
rs127653	T	C	T	C	0.00867	0.0154	0.23241	0.2588	FALSE	FALSE	FALSE	finn-b-K	10	7631014	0.04		0.6995	Chronic	Chronic	Chronic	TRUE	igd	7631014	0.0021	3.7E-05	10	89714	ukb-d-22	|| id:u	TRUE	reported	igd	2	TRUE
rs128669	A	G	A	G	0.02524	0.0166	0.02351	0.02974	FALSE	FALSE	FALSE	finn-b-K	13	110066100	0.1014		0.8699	Chronic	Chronic	Chronic	TRUE	igd	1.1E+08	0.00579	1.3E-05	13	89714	ukb-d-22	|| id:u	TRUE	reported	igd	2	TRUE
rs138858	A	G	A	G	0.04308	-0.0776	0.00736	0.03532	FALSE	FALSE	FALSE	finn-b-K	15	79099986	0.093		0.4042	Chronic	Chronic	Chronic	TRUE	igd	7.9E+07	0.01047	3.9E-05	15	89714	ukb-d-22	|| id:u	TRUE	reported	igd	2	TRUE
rs140647	G	A	G	A	0.04379	0.1015	0.0076	0.01553	FALSE	FALSE	FALSE	finn-b-K	19	20290042	0.1413		0.4727	Chronic	Chronic	Chronic	TRUE	igd	2E+07	0.01059	3.6E-05	19	89714	ukb-d-22	|| id:u	TRUE	reported	igd	2	TRUE
rs142563	A	G	A	G	-0.0344	-0.1297	0.01473	0.02004	FALSE	FALSE	FALSE	finn-b-K	13	23278334	0.124		0.2954	Chronic	Chronic	Chronic	TRUE	igd	2.3E+07	0.00745	4E-06	13	89714	ukb-d-22	|| id:u	TRUE	reported	igd	2	TRUE
rs143255	C	T	C	T	0.01694	0.0201	0.04814	0.04658	FALSE	FALSE	FALSE	finn-b-K	7	14743184	0.0822		0.807	Chronic	Chronic	Chronic	TRUE	igd	1.5E+08	0.00413	4.1E-05	7	89714	ukb-d-22	|| id:u	TRUE	reported	igd	2	TRUE
rs144479	A	G	A	G	0.05832	-0.1478	0.00476	0.0075	FALSE	FALSE	FALSE	finn-b-K	3	12272824	0.2061		0.4732	Chronic	Chronic	Chronic	TRUE	igd	1.2E+08	0.01297	6.9E-06	3	89714	ukb-d-22	|| id:u	TRUE	reported	igd	2	TRUE
rs145817	T	C	T	C	0.00807	-0.0098	0.54956	0.569	FALSE	FALSE	FALSE	finn-b-K	12	41777144	0.0351		0.7791	Chronic	Chronic	Chronic	TRUE	igd	4.2E+07	0.00176	4.4E-06	12	89714	ukb-d-22	|| id:u	TRUE	reported	igd	2	TRUE
rs147567	A	G	A	G	0.01947	-0.2869	0.03983	0.00762	FALSE	FALSE	FALSE	finn-b-K	7	47544762	0.2099		0.1718	Chronic	Chronic	Chronic	TRUE	igd	4.8E+07	0.0047	3.5E-05	7	89714	ukb-d-22	|| id:u	TRUE	reported	igd	2	TRUE
rs148904	C	G	C	G	0.03265	-0.1904	0.01511	0.00497	FALSE	TRUE	FALSE	finn-b-K	22	25249949	0.2561		0.4573	Chronic	Chronic	Chronic	TRUE	igd	2.5E+07	0.0076	1.7E-05	22	89714	ukb-d-22	|| id:u	TRUE	reported	igd	2	TRUE
rs149651	G	C	G	C	0.03813	-0.1646	0.01176	0.01374	FALSE	TRUE	FALSE	finn-b-K	6	16059547	0.1516		0.2779	Chronic	Chronic	Chronic	TRUE	igd	1.6E+08	0.00836	5E-06	6	89714	ukb-d-22	|| id:u	TRUE	reported	igd	2	TRUE
rs149772	T	A	T	A	0.0282	0.0044	0.02041	0.00863	FALSE	TRUE	FALSE	finn-b-K	4	12576228	0.1933		0.9817	Chronic	Chronic	Chronic	TRUE	igd	1.3E+07	0.00662	2E-05	4	89714	ukb-d-22	|| id:u	TRUE	reported	igd	2	TRUE
rs150100	C	T	C	T	0.02882	-0.3292	0.02023	0.01257	FALSE	FALSE	FALSE	finn-b-K	3	96018959	0.1586		0.03786	Chronic	Chronic	Chronic	TRUE	igd	9.6E+07	0.00671	1.8E-05	3	89714	ukb-d-22	|| id:u	TRUE	reported	igd	2	TRUE
rs150689	A	G	A	G	0.03224	-0.1779	0.0145	0.00592	FALSE	FALSE	FALSE	finn-b-K	13	23446586	0.2345		0.4482	Chronic	Chronic	Chronic	TRUE	igd	2.3E+07	0.00765	2.5E-05	13	89714	ukb-d-22	|| id:u	TRUE	reported	igd	2	TRUE
rs171494	G	A	G	A	0.01389	-0.0843	0.08945	0.1261	FALSE	FALSE	FALSE	finn-b-K	2	23526520	0.0526		0.1093	Chronic	Chronic	Chronic	TRUE	igd	2.4E+08	0.00314	9.5E-06	2	89714	ukb-d-22	|| id:u	TRUE	reported	igd	2	TRUE
rs176026	A	G	A	G	0.03352	0.3127	0.01586	0.00817	FALSE	FALSE	FALSE	finn-b-K	12	42480604	0.1972		0.1127	Chronic	Chronic	Chronic	TRUE	igd	4.2E+07	0.00714	2.7E-06	12	89714	ukb-d-22	|| id:u	TRUE	reported	igd	2	TRUE
rs189879	T	G	T	G	0.02926	0.0896	0.01658	0.01577	FALSE	FALSE	FALSE	finn-b-K	4	69058096	0.1411		0.5256	Chronic	Chronic	Chronic	TRUE	igd	6.9E+07	0.00705	3.3E-05	4	89714	ukb-d-22	|| id:u	TRUE	reported	igd	2	TRUE
rs192230	A	G	A	G	0.09947	-0.0285	0.00159	0.05074	FALSE	FALSE	FALSE	finn-b-K	19	50188478	0.0787		0.7169	Chronic	Chronic	Chronic	TRUE	igd	5E+07	0.02342	2.2E-05	19	89714	ukb-d-22	|| id:u	TRUE	reported	igd	2	TRUE
rs192463	T	A	T	A	0.02663	0.0321	0.01955	0.00893	FALSE	TRUE	FALSE	finn-b-K	2	21374412	0.1847		0.8619	Chronic	Chronic	Chronic	TRUE	igd	2.1E+08	0.00644	3.6E-05	2	89714	ukb-d-22	|| id:u	TRUE	reported	igd	2	TRUE
rs199341	G	C	G	C	0.0083	-0.045	0.25339	0.223	FALSE	TRUE	FALSE	finn-b-K	10	63400853	0.0419		0.283	Chronic	Chronic	Chronic	TRUE	igd	6.3E+07	0.00202	3.9E-05	10	89714	ukb-d-22	|| id:u	TRUE	reported	igd	2	TRUE
rs203146	C	T	C	T	0.00758	0.0005	0.62391	0.7287	FALSE	FALSE	FALSE	finn-b-K	10	13192344	0.0393		0.9906	Chronic	Chronic	Chronic	TRUE	igd	1.3E+08	0.00181	2.9E-05	10	89714	ukb-d-22	|| id:u	TRUE	reported	igd	2	TRUE
rs206940	G	A	G	A	-0.0084	-0.0116	0.34093	0.3132	FALSE	FALSE	FALSE	finn-b-K	12	56364321	0.0377		0.7593	Chronic	Chronic	Chronic	TRUE	igd	5.6E+07	0.00185	5.7E-06	12	89714	ukb-d-22	|| id:u	TRUE	reported	igd	2	TRUE
rs207008	G	T	G	T	-0.0196	-0.083	0.94188	0.9494	FALSE	FALSE	FALSE	finn-b-K	21	18735936	0.0797		0.298	Chronic	Chronic	Chronic	TRUE	igd	1.9E+07	0.00375	1.8E-07	21	89714	ukb-d-22	|| id:u	TRUE	reported	igd	2	TRUE
rs229649	G	A	G	A	-0.0113	-0.0687	0.11402	0.1313	FALSE	FALSE	FALSE	finn-b-K	13	95096111	0.0513		0.1809	Chronic	Chronic	Chronic	TRUE	igd	9.5E+07	0.00277	4.3E-05	13	89714	ukb-d-22	|| id:u	TRUE	reported	igd	2	TRUE
rs268312	C	A	C	A	-0.0072	0.0064	0.46584	0.5128	FALSE	FALSE	FALSE	finn-b-K	15	36024804	0.0351		0.8551	Chronic	Chronic	Chronic	TRUE	igd	3.6E+07	0.00177	4.9E-05	15	89714	ukb-d-22	|| id:u	TRUE	reported	igd	2	TRUE
rs276583	A	G	A	G	0.01277	-0.109	0.12036	0.08095	FALSE	FALSE	FALSE	finn-b-K	10	21034317	0.0646		0.09178	Chronic	Chronic	Chronic	TRUE	igd	2.1E+07	0.00272	2.7E-06	10	89714	ukb-d-22	|| id:u	TRUE	reported	igd	2	TRUE
rs284717	T	C	T	C	0.00882	-0.0066	0.63416	0.6131	FALSE	FALSE	FALSE	finn-b-K	11	10587762	0.0358		0.8548	Chronic	Chronic	Chronic	TRUE	igd	1.1E+08	0.00183	1.4E-06	11	89714	ukb-d-22	|| id:u	TRUE	reported	igd	2	TRUE
rs309354	A	G	A	G	-0.0185	-0.0419	0.04632	0.1383	FALSE	FALSE	FALSE	finn-b-K	6	31539010	0.0753		0.5778	Chronic	Chronic	Chronic	TRUE	igd	3.2E+07	0.00418	9.8E-06	6	89714	ukb-d-22	|| id:u	TRUE	reported	igd	2	TRUE
rs310653	A	C	A	C	0.01211	-0.045	0.10649	0.1596	FALSE	FALSE	FALSE	finn-b-K	8	13125914	0.0479		0.3472	Chronic	Chronic	Chronic	TRUE	igd	1.3E+08	0.00284	2E-05	8	89714	ukb-d-22	|| id:u	TRUE	reported	igd	2	TRUE
rs353857	T	A	T	A	0.00794	-0.0297	0.63465	0.5767	FALSE	TRUE	TRUE	finn-b-K	3	19607183	0.0352		0.3981	Chronic	Chronic	Chronic	TRUE	igd	2E+08	0.00183	1.4E-05	3	89714	ukb-d-22	|| id:u	TRUE	reported	igd	2	FALSE
rs370870	A	G	A	G	0.01649	-0.0405	0.05074	0.1188	FALSE	FALSE	FALSE	finn-b-K	1	24598117	0.0534		0.448	Chronic	Chronic	Chronic	TRUE	igd	2.5E+08	0.00402	4.2E-05	1	89714	ukb-d-22	|| id:u	TRUE	reported	igd	2	TRUE
rs373969	A	G	A	G	0.00727	-0.0654	0.49641	0.5088	FALSE	FALSE	FALSE	finn-b-K	9	11479894	0.035		0.06133	Chronic	Chronic	Chronic	TRUE	igd	1.1E+08	0.00177	3.9E-05	9	89714	ukb-d-22	|| id:u	TRUE	reported	igd	2	TRUE
rs382594	A	G	A	G	0.00829	0.0509	0.25777	0.2575	FALSE	FALSE	FALSE	finn-b-K	15	74317362	0.0398		0.2014	Chronic	Chronic	Chronic	TRUE	igd	7.4E+07	0.00202	3.9E-05	15	89714	ukb-d-22	|| id:u	TRUE	reported	igd	2	TRUE
rs389792	T	G	T	G	-0.0075	-0.0263	0.431	0.2888	FALSE	FALSE	FALSE	finn-b-K	3	10170305	0.0388		0.498	Chronic	Chronic	Chronic	TRUE	igd	1E+08	0.00177	2.1E-05	3	89714	ukb-d-22	|| id:u	TRUE	reported	igd	2	TRUE
rs413194	T	G	T	G	-0.0074	0.0153	0.51371	0.551	FALSE	FALSE	FALSE	finn-b-K	16	54862861	0.0352		0.6629	Chronic	Chronic	Chronic	TRUE	igd	5.5E+07	0.00178	3.1E-05	16	89714	ukb-d-22	|| id:u	TRUE	reported	igd	2	TRUE
rs443395	T	A	T	A	0.0078	-0.001	0.43142	0.3898	FALSE	TRUE	TRUE	finn-b-K	2	23811540	0.0357		0.9774	Chronic	Chronic	Chronic	TRUE	igd	2.4E+08	0.00182	1.7E-05	2	89714	ukb-d-22	|| id:u	TRUE	reported	igd	2	FALSE
rs468829	T	C	T	C	0.01399	0.0186	0.07414	0.04714	FALSE	FALSE	FALSE	finn-b-K	3	62460883	0.0826		0.8216	Chronic	Chronic	Chronic	TRUE	igd	6.2E+07	0.00341	4E-05	3	89714	ukb-d-22	|| id:u	TRUE	reported	igd	2	TRUE
rs481445	C	T	C	T	-0.0168	-0.0962	0.94596	0.9129	FALSE	FALSE	FALSE	finn-b-K	20	16178198	0.0617		0.1189	Chronic	Chronic	Chronic	TRUE	igd	1.6E+07	0.00387	1.5E-05	20	89714	ukb-d-22	|| id:u	TRUE	reported	igd	2	TRUE
rs497501	A	G	A	G	0.00903	-0.0103	0.22088	0.2506	FALSE	FALSE	FALSE	finn-b-K	4	39423512	0.04		0.797	Chronic	Chronic	Chronic	TRUE	igd	3.9E+07	0.00213	2.2E-05	4	89714	ukb-d-22	|| id:u	TRUE	reported	igd	2	TRUE
rs513564	T	C	T	C	0.00846	0.0421	0.24995	0.1786	FALSE	FALSE	FALSE	finn-b-K	10	99780908	0.0461		0.3612	Chronic	Chronic	Chronic	TRUE	igd	1E+08	0.00207	4.4E-05	10	89714	ukb-d-22	|| id:u	TRUE	reported	igd	2	TRUE
rs554912	C	G	C	G	-0.0124	-0.0591	0.10323	0.1183	FALSE	TRUE	FALSE	finn-b-K	1	22673791	0.0544		0.2769	Chronic	Chronic	Chronic	TRUE	igd	2.3E+08	0.00295	2.8E-05	1	89714	ukb-d-22	|| id:u	TRUE	reported	igd	2	TRUE
rs558136	T	C	T	C	0.03104	-0.1668	0.01488	0.01198	FALSE	FALSE	FALSE	finn-b-K	8	62318022	0.1621		0.3035	Chronic	Chronic	Chronic	TRUE	igd	6.2E+07	0.00726	1.9E-05	8	89714	ukb-d-22	|| id:u	TRUE	reported	igd	2	TRUE
rs559280	A	G	A	G	-0.026	-0.1877	0.02501	0.01587	FALSE	FALSE	FALSE	finn-b-K	16	69750910	0.1438		0.192	Chronic	Chronic	Chronic	TRUE	igd	7E+07	0.00566	4.2E-06	16	89714	ukb-d-22	|| id:u	TRUE	reported	igd	2	TRUE
rs561694	T	C	T	C	-0.0141	0.0585	0.07356	0.05482	FALSE	FALSE	FALSE	finn-b-K	12	11715547	0.0782		0.4542	Chronic	Chronic	Chronic	TRUE	igd	1.2E+08	0.00347	4.9E-05	12	89714	ukb-d-22	|| id:u	TRUE	reported	igd	2	TRUE
rs576053	C	G	C	G	-0.0218	-0.0157	0.02771	0.04113	FALSE	TRUE	FALSE	finn-b-K	6	15183577	0.0874		0.8572	Chronic	Chronic	Chronic	TRUE	igd	1.5E+08	0.00534	4.6E-05	6	89714	ukb-d-22	|| id:u	TRUE	reported	igd	2	TRUE
rs611254	T	C	T	C	-0.0151	0.0306	0.08847	0.07035	FALSE	FALSE	FALSE	finn-b-K	20	19727485	0.0678		0.6516	Chronic	Chronic	Chronic	TRUE	igd	2E+07	0.00308	8.6E-07	20	89714	ukb-d-22	|| id:u	TRUE	reported	igd	2	TRUE
rs613060	C	G	C	G	-0.0103	0.0498	0.19003	0.2568	FALSE	TRUE	FALSE	finn-b-K	20	43033095	0.0399		0.2119	Chronic	Chronic	Chronic	TRUE	igd	4.3E+07	0.00223	3.8E-06	20	89714	ukb-d-22	|| id:u	TRUE	reported	igd	2	TRUE
rs620553	G	T	G	T	-0.014	-0.1209	0.08724	0.06716	FALSE	FALSE	FALSE	finn-b-K	16	30187673	0.0707		0.08731	Chronic	Chronic	Chronic	TRUE	igd	3E+07	0.00321	1.4E-05	16	89714	ukb-d-22	|| id:u	TRUE	reported	igd	2	TRUE
rs622080	G	A	G	A	0.02549	0.0001	0.02231	0.01686	FALSE	FALSE	FALSE	finn-b-K	20	62043227	0.1355		0.9994	Chronic	Chronic	Chronic	TRUE	igd	6.2E+07	0.00592	1.6E-05	20	89714	ukb-d-22	|| id:u	TRUE	reported	igd	2	TRUE
rs624773	T	A	T	A	0.02016	-0.1098	0.04486	0.04382	FALSE	TRUE	FALSE	finn-b-K	7	11493527	0.0844		0.193	Chronic	Chronic	Chronic	TRUE	igd	1.1E+08	0.00426	2.2E-06	7	89714	ukb-d-22	|| id:u	TRUE	reported	igd	2	TRUE
rs624961	T	C	T	C	0.02838	0.2082	0.02176	0.00764	FALSE	FALSE	FALSE	finn-b-K	8	9288470	0.2099		0.3213	Chronic	Chronic	Chronic	TRUE	igd	9288470	0.00632	7.2E-06	8	89714	ukb-d-22	|| id:u	TRUE	reported	igd	2	TRUE
rs688129	C	T	C	T	0.00745	0.011	0.58387	0.5651	FALSE	FALSE	FALSE	finn-b-K	5	11147494	0.035		0.7531	Chronic	Chronic	Chronic	TRUE	igd	1.1E+07	0.00177	2.7E-05	5	89714	ukb-d-22	|| id:u	TRUE	reported	igd	2	TRUE
rs689896	G	C	G	C	0.00762	0.0091	0.63769	0.6836	FALSE	TRUE	FALSE	finn-b-K	18	74078911	0.0377		0.81	Chronic	Chronic	Chronic	TRUE	igd	7.4E+07	0.00182	2.8E-05	18	89714	ukb-d-22	|| id:u	TRUE	reported	igd	2	TRUE
rs691490	C	A	C	A	0.01026	0.0632	0.14903	0.1591	FALSE	FALSE	FALSE	finn-b-K	6	13902152	0.0477		0.1849	Chronic	Chronic	Chronic	TRUE	igd	1.4E+08	0.00249	3.7E-05	6	89714	ukb-d-22	|| id:u	TRUE	reported	igd	2	TRUE
rs708246	A	G	A	G	-0.0085	-0.0435	0.67891	0.583	FALSE	FALSE	FALSE	finn-b-K	10	50486727	0.0353		0.2183	Chronic	Chronic	Chronic	TRUE	igd	5E+07	0.00188	6.2E-06	10	89714	ukb-d-22	|| id:u	TRUE	reported	igd	2	TRUE
rs720454	G	C	G	C	0.00922	0.0139	0.69183	0.5858	FALSE	TRUE	FALSE	finn-b-K	16	718514	0.0355		0.6947	Chronic	Chronic	Chronic	TRUE	igd	718514	0.00197	2.8E-06	16	89714	ukb-d-22	|| id:u	TRUE	reported	igd	2	TRUE
rs726791	A	G	A	G	0.01238	0.016	0.09252	0.1885	FALSE	FALSE	FALSE	finn-b-K	1	66625369	0.0445		0.7186	Chronic	Chronic	Chronic	TRUE	igd	6.7E+07	0.00303	4.5E-05	1	89714	ukb-d-22	|| id:u	TRUE	reported	igd	2	TRUE
rs727260	G	C	G	C	-0.0115	-0.0476	0.1251	0.1171	FALSE	TRUE	FALSE	finn-b-K	9	38072104	0.0538		0.3767	Chronic	Chronic	Chronic	TRUE	igd	3.8E+07	0.00267	1.5E-05	9	89714	ukb-d-22	|| id:u	TRUE	reported	igd	2	TRUE
rs727603	C	T	C	T	0.03144	-0.0153	0.01409	0.02608	FALSE	FALSE	FALSE	finn-b-K	15	84192374	0.1106		0.8901	Chronic	Chronic	Chronic	TRUE	igd	8.4E+07	0.00758	3.3E-05	15	89714	ukb-d-22	|| id:u	TRUE	reported	igd	2	TRUE
rs728961	A	G	A	G	0.0118	0.0113	0.1081	0.06909	FALSE	FALSE	FALSE	finn-b-K	11	5845481	0.0693		0.8706	Chronic	Chronic	Chronic	TRUE	igd	5845481	0.00291	5E-05	11	89714	ukb-d-22	|| id:u	TRUE	reported	igd	2	TRUE
rs732141	C	A	C	A	-0.0088	-0.0178	0.24763	0.2011	FALSE	FALSE	FALSE	finn-b-K	12	12147609	0.0437		0.6842	Chronic	Chronic	Chronic	TRUE	igd	1.2E+08	0.00203	1.7E-05	12	89714	ukb-d-22	|| id:u	TRUE	reported	igd	2	TRUE
rs733601	C	T	C	T	-0.0085	0.0108	0.25051	0.2141	FALSE	FALSE	FALSE	finn-b-K	21	41403037	0.0427		0.8	Chronic	Chronic	Chronic	TRUE	igd	4.1E+07	0.00203	3E-05	21	89714	ukb-d-22	|| id:u	TRUE	reported	igd	2	TRUE
rs733619	C	T	C	T	-0.0073	-0.0342	0.44239	0.4566	FALSE	FALSE	FALSE	finn-b-K	13	53914953	0.0348		0.3258	Chronic	Chronic	Chronic	TRUE	igd	5.4E+07	0.00178	4.3E-05	13	89714	ukb-d-22	|| id:u	TRUE	reported	igd	2	TRUE
rs744051	A	G	A	G	-0.0229	0.0547	0.02571	0.03513	FALSE	FALSE	FALSE	finn-b-K	2	20871152	0.0944		0.562	Chronic	Chronic	Chronic	TRUE	igd	2.1E+08	0.00563	5E-05	2	89714	ukb-d-22	|| id:u	TRUE	reported	igd	2	TRUE
rs746397	T	A	T	A	0.01254	-0.0114	0.08923	0.118	FALSE	TRUE	FALSE	finn-b-K	2	14488536	0.0541		0.8333	Chronic	Chronic	Chronic	TRUE	igd	1.4E+08	0.00307	4.5E-05	2	89714	ukb-d-22	|| id:u	TRUE	reported	igd	2	TRUE
rs762178	G	A	G	A	0.04401	0.0752	0.00963	0.01253	FALSE	FALSE	FALSE	finn-b-K	8	94600393	0.1568		0.6315	Chronic	Chronic	Chronic	TRUE	igd	9.5E+07	0.00898	9.5E-07	8	89714	ukb-d-22	|| id:u	TRUE	reported	igd	2	TRUE
rs768794	T	C	T	C	-0.0227	-0.1378	0.02696	0.00923	FALSE	FALSE	FALSE	finn-b-K	10	87990995	0.1828		0.4509	Chronic	Chronic	Chronic	TRUE	igd	8.8E+07	0.00541	2.8E-05	10	89714	ukb-d-22	|| id:u	TRUE	reported	igd	2	TRUE
rs772112	A	T	A	T	0.01135	0.0818	0.86874	0.8082	FALSE	TRUE	FALSE	finn-b-K	17	33621127	0.0446		0.06631	Chronic	Chronic	Chronic	TRUE	igd	3.4E+07	0.00265	1.8E-05	17	89714	ukb-d-22	|| id:u	TRUE	reported	igd	2	TRUE
rs773097	A	C	A	C	0.02921	-0.1085	0.01555	0.03935	FALSE	FALSE	FALSE	finn-b-K	7	73663178	0.0883		0.2192	Chronic	Chronic	Chronic	TRUE	igd	7.4E+07	0.00712	4.1E-05	7	89714	ukb-d-22	|| id:u	TRUE	reported	igd	2	TRUE
rs778055	T	G	T	G	0.01932	0.0377	0.05764	0.06151	FALSE	FALSE	FALSE	finn-b-K	6	14483431	0.0727		0.6045	Chronic	Chronic	Chronic	TRUE	igd	1.4E+08	0.0038	3.6E-07	6	89714	ukb-d-22	|| id:u	TRUE	reported	igd	2	TRUE
rs794986	G	A	G	A	-0.0277	-0.0387	0.02296	0.00416	FALSE	FALSE	FALSE	finn-b-K	8	13485528	0.2744		0.8879	Chronic	Chronic	Chronic	TRUE	igd	1.3E+08	0.00583	2E-06	8	89714	ukb-d-22	|| id:u	TRUE	reported	igd	2	TRUE
rs795195	C	T	C	T	-0.0218	0.0768	0.02895	0.01245	FALSE	FALSE	FALSE	finn-b-K	14	34720312	0.1631		0.6378	Chronic	Chronic	Chronic	TRUE	igd	3.5E+07	0.00521	2.9E-05	14	89714	ukb-d-22	|| id:u	TRUE	reported	igd	2	TRUE
rs797808	C	T	C	T	0.02474	-0.1181	0.02269	0.00826	FALSE	FALSE	FALSE	finn-b-K	7	10424795	0.1916		0.5374	Chronic	Chronic	Chronic	TRUE	igd	1E+08	0.00593	3E-05	7	89714	ukb-d-22	|| id:u	TRUE	reported	igd	2	TRUE
rs800655	C	G	C	G	0.01243	-0.0174	0.15964	0.179	FALSE	TRUE	FALSE	finn-b-K	14	67294069	0.0455		0.7025	Chronic	Chronic	Chronic	TRUE	igd	6.7E+07	0.00244	3.7E-07	14	89714	ukb-d-22	|| id:u	TRUE	reported	igd	2	TRUE
rs876462	G	T	G	T	-0.008	-0.0106	0.37434	0.4562	FALSE	FALSE	FALSE	finn-b-K	2	37515880	0.0349		0.7612	Chronic	Chronic	Chronic	TRUE	igd	3.8E+07	0.00181	1E-05	2	89714	ukb-d-22	|| id:u	TRUE	reported	igd	2	TRUE
rs884258	C	T	C	T	-0.0079	-0.0028	0.67693	0.6535	FALSE	FALSE	FALSE	finn-b-K	9	10705717	0.0365		0.9394	Chronic	Chronic	Chronic	TRUE	igd	1.1E+08	0.00189	3.3E-05	9	89714	ukb-d-22	|| id:u	TRUE	reported	igd	2	TRUE
rs914186	A	G	A	G	0.00773	0.0518	0.66006	0.6035	FALSE	FALSE	FALSE	finn-b-K	21	42619657	0.0355		0.1446	Chronic	Chronic	Chronic	TRUE	igd	4.3E+07	0.00186	3.3E-05	21	89714	ukb-d-22	|| id:u	TRUE	reported	igd	2	TRUE
rs936835	T	A	T	A	0.03017	0.1842	0.01474	0.03726	FALSE	TRUE	FALSE	finn-b-K	6	21808192	0.0915		0.04401	Chronic	Chronic	Chronic	TRUE	igd	2.2E+07	0.00733	3.9E-05	6	89714	ukb-d-22	|| id:u	TRUE	reported	igd	2	TRUE
rs953033	G	T	G	T	0.0077	0.016	0.65454	0.6283	FALSE	FALSE	FALSE	finn-b-K	13	75070201	0.036		0.6567	Chronic	Chronic	Chronic	TRUE	igd	7.5E+07	0.00185	3.1E-05	13	89714	ukb-d-22	|| id:u	TRUE	reported	igd	2	TRUE

Table S7. Detail Information of the Genetic Variants in the MR Analysis of SHS and IBD

SNP	effect_a	other_al	effect_a	other_al	beta.exr	beta.out	eaf.expc	eaf.outc	remove	palindrc	ambiguou	id.outcc	chr	pos	se.outcc	samplesi	pval.out	outcome	original	outcome.	mr_keep.	data_sou	pos.expos	se.expos	pval.exr	chr.expo	samplesi	id.expos	exposure	mr_keep.	pval_ori	data_sou	action	mr_keep
rs100090	A	G	A	G	-0.0076	-0.0084	0.53599	0.53326	FALSE	FALSE	FALSE	ieu-a-31	4	180336832	0.017	34652	0.6212	Inflamma	Inflamma	Inflamma	TRUE	igd	180336832	0.00176	1.4E-05	4	89714	ukb-d-22	|| id:u	TRUE	reported	igd	2	TRUE
rs102193	T	C	T	C	-0.0095	0.0213	0.1902	0.19537	FALSE	FALSE	FALSE	ieu-a-31	11	86339724	0.021	34652	0.3096	Inflamma	Inflamma	Inflamma	TRUE	igd	86339724	0.00224	2.4E-05	11	89714	ukb-d-22	|| id:u	TRUE	reported	igd	2	TRUE
rs104255	C	T	C	T	-0.0084	0.0145	0.70783	0.693	FALSE	FALSE	FALSE	ieu-a-31	19	836043	0.0264	34652	0.5831	Inflamma	Inflamma	Inflamma	TRUE	igd	836043	0.00204	3.6E-05	19	89714	ukb-d-22	|| id:u	TRUE	reported	igd	2	TRUE
rs109451	A	T	A	T	-0.0079	0.017	0.31369	0.301	FALSE	TRUE	FALSE	ieu-a-31	6	69354524	0.0183	34652	0.3548	Inflamma	Inflamma	Inflamma	TRUE	igd	69354524	0.00189	3.2E-05	6	89714	ukb-d-22	|| id:u	TRUE	reported	igd	2	TRUE
rs112169	A	G	A	G	0.03019	0.0159	0.01694	0.01044	FALSE	FALSE	FALSE	ieu-a-31	7	70641982	0.1006	34652	0.8746	Inflamma	Inflamma	Inflamma	TRUE	igd	70641982	0.00676	8.1E-06	7	89714	ukb-d-22	|| id:u	TRUE	reported	igd	2	TRUE
rs114134	T	C	T	C	0.04033	0.147	0.00945	0.01059	FALSE	FALSE	FALSE	ieu-a-31	6	41851676	0.116	34652	0.2049	Inflamma	Inflamma	Inflamma	TRUE	igd	41851676	0.00973	3.4E-05	6	89714	ukb-d-22	|| id:u	TRUE	reported	igd	2	TRUE
rs116249	T	C	T	C	-0.0289	-0.1002	0.01801	0.01852	FALSE	FALSE	FALSE	ieu-a-31	3	190482826	0.0784	34652	0.2015	Inflamma	Inflamma	Inflamma	TRUE	igd	190482826	0.00697	3.5E-05	3	89714	ukb-d-22	|| id:u	TRUE	reported	igd	2	TRUE
rs117410	G	A	G	A	-0.0415	0.0853	0.00888	0.011	FALSE	FALSE	FALSE	ieu-a-31	15	47979695	0.0861	34652	0.3216	Inflamma	Inflamma	Inflamma	TRUE	igd	47979695	0.00945	1.1E-05	15	89714	ukb-d-22	|| id:u	TRUE	reported	igd	2	TRUE
rs117514	T	G	T	G	0.00909	-0.0263	0.18988	0.18503	FALSE	FALSE	FALSE	ieu-a-31	22	49534383	0.0234	34652	0.2608	Inflamma	Inflamma	Inflamma	TRUE	igd	49534383	0.00223	4.5E-05	22	89714	ukb-d-22	|| id:u	TRUE	reported	igd	2	TRUE
rs117858	A	G	A	G	-0.0212	0.0989	0.03627	0.04024	FALSE	FALSE	FALSE	ieu-a-31	6	149289638	0.048	34652	0.03944	Inflamma	Inflamma	Inflamma	TRUE	igd	149289638	0.00484	1.2E-05	6	89714	ukb-d-22	|| id:u	TRUE	reported	igd	2	TRUE
rs118098	T	C	T	C	0.02983	0.1254	0.02017	0.0154	FALSE	FALSE	FALSE	ieu-a-31	21	33944718	0.0839	34652	0.1352	Inflamma	Inflamma	Inflamma	TRUE	igd	33944718	0.00656	5.4E-06	21	89714	ukb-d-22	|| id:u	TRUE	reported	igd	2	TRUE
rs118146	G	A	G	A	-0.0131	-0.0322	0.09386	0.07726	FALSE	FALSE	FALSE	ieu-a-31	8	22116219	0.0385	34652	0.4034	Inflamma	Inflamma	Inflamma	TRUE	igd	22116219	0.00312	2.5E-05	8	89714	ukb-d-22	|| id:u	TRUE	reported	igd	2	TRUE
rs120418	T	C	T	C	0.01394	-0.0106	0.08023	0.08219	FALSE	FALSE	FALSE	ieu-a-31	1	60484531	0.0314	34652	0.7347	Inflamma	Inflamma	Inflamma	TRUE	igd	60484531	0.00324	1.7E-05	1	89714	ukb-d-22	|| id:u	TRUE	reported	igd	2	TRUE
rs121057	G	T	G	T	0.00874	0.0032	0.36894	0.38463	FALSE	FALSE	FALSE	ieu-a-31	2	60736985	0.018	34652	0.8572	Inflamma	Inflamma	Inflamma	TRUE	igd	60736985	0.00184	2E-06	2	89714	ukb-d-22	|| id:u	TRUE	reported	igd	2	TRUE
rs124091	C	T	C	T	-0.0085	-0.0198	0.24869	0.25751	FALSE	FALSE	FALSE	ieu-a-31	1	114055488	0.02	34652	0.3214	Inflamma	Inflamma	Inflamma	TRUE	igd	114055488	0.00208	3.9E-05	1	89714	ukb-d-22	|| id:u	TRUE	reported	igd	2	TRUE
rs124840	T	C	T	C	0.00905	0.022	0.20587	0.19786	FALSE	FALSE	FALSE	ieu-a-31	22	51099862	0.0215	34652	0.306	Inflamma	Inflamma	Inflamma	TRUE	igd	51099862	0.00217	3E-05	22	89714	ukb-d-22	|| id:u	TRUE	reported	igd	2	TRUE
rs124932	G	T	G	T	0.01626	-0.0339	0.05371	0.05426	FALSE	FALSE	FALSE	ieu-a-31	3	16679114	0.0396	34652	0.3927	Inflamma	Inflamma	Inflamma	TRUE	igd	16679114	0.00388	2.8E-05	3	89714	ukb-d-22	|| id:u	TRUE	reported	igd	2	TRUE
rs126292	A	T	A	T	-0.0081	0.0115	0.33223	0.32537	FALSE	TRUE	FALSE	ieu-a-31	6	16806082	0.0187	34652	0.5388	Inflamma	Inflamma	Inflamma	TRUE	igd	16806082	0.00189	1.7E-05	6	89714	ukb-d-22	|| id:u	TRUE	reported	igd	2	TRUE
rs127653	T	C	T	C	0.00867	-0.0091	0.23241	0.23274	FALSE	FALSE	FALSE	ieu-a-31	10	7631014	0.0199	34652	0.6482	Inflamma	Inflamma	Inflamma	TRUE	igd	7631014	0.0021	3.7E-05	10	89714	ukb-d-22	|| id:u	TRUE	reported	igd	2	TRUE
rs128669	A	G	A	G	0.02524	0.1074	0.02351	0.02359	FALSE	FALSE	FALSE	ieu-a-31	13	110066103	0.0551	34652	0.05117	Inflamma	Inflamma	Inflamma	TRUE	igd	110066103	0.00579	1.3E-05	13	89714	ukb-d-22	|| id:u	TRUE	reported	igd	2	TRUE
rs138858	A	G	A	G	0.04308	-0.0507	0.00736	0.01161	FALSE	FALSE	FALSE	ieu-a-31	15	79099986	0.0926	34652	0.5841	Inflamma	Inflamma	Inflamma	TRUE	igd	79099986	0.01047	3.9E-05	15	89714	ukb-d-22	|| id:u	TRUE	reported	igd	2	TRUE
rs140647	G	A	G	A	0.04379	0.0225	0.0076	0.00837	FALSE	FALSE	FALSE	ieu-a-31	19	20290042	0.1072	34652	0.8335	Inflamma	Inflamma	Inflamma	TRUE	igd	20290042	0.01059	3.6E-05	19	89714	ukb-d-22	|| id:u	TRUE	reported	igd	2	TRUE
rs142563	A	G	A	G	-0.0344	0.0503	0.01473	0.01894	FALSE	FALSE	FALSE	ieu-a-31	13	23278334	0.0689	34652	0.4651	Inflamma	Inflamma	Inflamma	TRUE	igd	23278334	0.00745	4E-06	13	89714	ukb-d-22	|| id:u	TRUE	reported	igd	2	TRUE
rs143255	C	T	C	T	0.01694	-0.0171	0.04814	0.05363	FALSE	FALSE	FALSE	ieu-a-31	7	147431844	0.0392	34652	0.6631	Inflamma	Inflamma	Inflamma	TRUE	igd	147431844	0.00413	4.1E-05	7	89714	ukb-d-22	|| id:u	TRUE	reported	igd	2	TRUE
rs144479	A	G	A	G	0.05832	-0.1033	0.00476	0.00907	FALSE	FALSE	FALSE	ieu-a-31	3	122728248	0.1142	34652	0.3656	Inflamma	Inflamma	Inflamma	TRUE	igd	122728248	0.01297	6.9E-06	3	89714	ukb-d-22	|| id:u	TRUE	reported	igd	2	TRUE
rs145817	T	C	T	C	0.00807	0.0097	0.54956	0.57023	FALSE	FALSE	FALSE	ieu-a-31	12	41777144	0.0169	34652	0.5674	Inflamma	Inflamma	Inflamma	TRUE	igd	41777144	0.00176	4.4E-06	12	89714	ukb-d-22	|| id:u	TRUE	reported	igd	2	TRUE
rs147567	A	G	A	G	0.01947	-0.0262	0.03983	0.04063	FALSE	FALSE	FALSE	ieu-a-31	7	47544762	0.0507	34652	0.6058	Inflamma	Inflamma	Inflamma	TRUE	igd	47544762	0.0047	3.5E-05	7	89714	ukb-d-22	|| id:u	TRUE	reported	igd	2	TRUE
rs148904	C	G	C	G	0.03265	-0.0682	0.01511	0.0171	FALSE	TRUE	FALSE	ieu-a-31	22	25249949	0.0808	34652	0.3987	Inflamma	Inflamma	Inflamma	TRUE	igd	25249949	0.0076	1.7E-05	22	89714	ukb-d-22	|| id:u	TRUE	reported	igd	2	TRUE
rs149651	G	C	G	C	0.03813	0.1805	0.01176	0.01137	FALSE	TRUE	FALSE	ieu-a-31	6	160595479	0.0833	34652	0.03032	Inflamma	Inflamma	Inflamma	TRUE	igd	160595479	0.00836	5E-06	6	89714	ukb-d-22	|| id:u	TRUE	reported	igd	2	TRUE
rs149772	T	A	T	A	0.0282	0.0235	0.02041	0.02037	FALSE	TRUE	FALSE	ieu-a-31	4	12576228	0.0729	34652	0.7473	Inflamma	Inflamma	Inflamma	TRUE	igd	12576228	0.00662	2E-05	4	89714	ukb-d-22	|| id:u	TRUE	reported	igd	2	TRUE
rs150100	C	T	C	T	0.02882	-0.0668	0.02023	0.02226	FALSE	FALSE	FALSE	ieu-a-31	3	96018959	0.0567	34652	0.2391	Inflamma	Inflamma	Inflamma	TRUE	igd	96018959	0.00671	1.8E-05	3	89714	ukb-d-22	|| id:u	TRUE	reported	igd	2	TRUE
rs150689	A	G	A	G	0.03224	-0.0642	0.0145	0.01418	FALSE	FALSE	FALSE	ieu-a-31	13	23446586	0.0898	34652	0.4747	Inflamma	Inflamma	Inflamma	TRUE	igd	23446586	0.00765	2.5E-05	13	89714	ukb-d-22	|| id:u	TRUE	reported	igd	2	TRUE
rs171494	G	A	G	A	0.01389	-0.0753	0.08945	0.10414	FALSE	FALSE	FALSE	ieu-a-31	2	235265207	0.0309	34652	0.01489	Inflamma	Inflamma	Inflamma	TRUE	igd	235265207	0.00314	9.5E-06	2	89714	ukb-d-22	|| id:u	TRUE	reported	igd	2	TRUE
rs176026	A	G	A	G	0.03352	-0.0878	0.01586	0.01671	FALSE	FALSE	FALSE	ieu-a-31	12	42480604	0.0694	34652	0.2053	Inflamma	Inflamma	Inflamma	TRUE	igd	42480604	0.00714	2.7E-06	12	89714	ukb-d-22	|| id:u	TRUE	reported	igd	2	TRUE
rs189879	T	G	T	G	0.02926	-0.0614	0.01658	0.00664	FALSE	FALSE	FALSE	ieu-a-31	4	69058096	0.1399	34652	0.6607	Inflamma	Inflamma	Inflamma	TRUE	igd	69058096	0.00705	3.3E-05	4	89714	ukb-d-22	|| id:u	TRUE	reported	igd	2	TRUE
rs192230	A	G	A	G	0.09947	-0.1856	0.00159	0.00933	FALSE	FALSE	FALSE	ieu-a-31	19	50188478	0.1309	34652	0.1561	Inflamma	Inflamma	Inflamma	TRUE	igd	50188478	0.02342	2.2E-05	19	89714	ukb-d-22	|| id:u	TRUE	reported	igd	2	TRUE
rs192463	T	A	T	A	0.02663	-0.0012	0.01955	0.01263	FALSE	TRUE	FALSE	ieu-a-31	2	213744121	0.0815	34652	0.9882	Inflamma	Inflamma	Inflamma	TRUE	igd	213744121	0.00644	3.6E-05	2	89714	ukb-d-22	|| id:u	TRUE	reported	igd	2	TRUE
rs199341	G	C	G	C	0.0083	0.024	0.25339	0.26686	FALSE	TRUE	FALSE	ieu-a-31	10	63400853	0.019	34652	0.2074	Inflamma	Inflamma	Inflamma	TRUE	igd	63400853	0.00202	3.9E-05	10	89714	ukb-d-22	|| id:u	TRUE	reported	igd	2	TRUE
rs203146	C	T	C	T	0.00758	0.0053	0.62391	0.621	FALSE	FALSE	FALSE	ieu-a-31	10	131923448	0.0179	34652	0.767	Inflamma	Inflamma	Inflamma	TRUE	igd	131923448	0.00181	2.9E-05	10	89714	ukb-d-22	|| id:u	TRUE	reported	igd	2	TRUE
rs206940	G	A	G	A	-0.0084	0.0004	0.34093	0.32726	FALSE	FALSE	FALSE	ieu-a-31	12	56364321	0.0178	34652	0.9802	Inflamma	Inflamma	Inflamma	TRUE	igd	56364321	0.00185	5.7E-06	12	89714	ukb-d-22	|| id:u	TRUE	reported	igd	2	TRUE
rs207008	G	T	G	T	-0.0196	0.0022	0.94188	0.9389	FALSE	FALSE	FALSE	ieu-a-31	21	18735936	0.0364	34652	0.9528	Inflamma	Inflamma	Inflamma	TRUE	igd	18735936	0.00375	1.8E-07	21	89714	ukb-d-22	|| id:u	TRUE	reported	igd	2	TRUE
rs229649	G	A	G	A	-0.0113	-0.0085	0.11402	0.12074	FALSE	FALSE	FALSE	ieu-a-31	13	95096111	0.0258	34652	0.741	Inflamma	Inflamma	Inflamma	TRUE	igd	95096111	0.00277	4.3E-05	13	89714	ukb-d-22	|| id:u	TRUE	reported	igd	2	TRUE
rs268312	C	A	C	A	-0.0072	0.0056	0.46584	0.46363	FALSE	FALSE	FALSE	ieu-a-31	15	36024804	0.0175	34652	0.7474	Inflamma	Inflamma	Inflamma	TRUE	igd	36024804	0.00177	4.9E-05	15	89714	ukb-d-22	|| id:u	TRUE	reported	igd	2	TRUE
rs276583	A	G	A	G	0.01277	0.0498	0.12036	0.12286	FALSE	FALSE	FALSE	ieu-a-31	10	21034317	0.0268	34652	0.0629	Inflamma	Inflamma	Inflamma	TRUE	igd	21034317	0.00272	2.7E-06	10	89714	ukb-d-22	|| id:u	TRUE	reported	igd	2	TRUE
rs284717	T	C	T	C	0.00882	0.0133	0.63416	0.64423	FALSE	FALSE	FALSE	ieu-a-31	11	105877624	0.0176	34652	0.4483	Inflamma	Inflamma	Inflamma	TRUE	igd	105877624	0.00183	1.4E-06	11	89714	ukb-d-22	|| id:u	TRUE	reported	igd	2	TRUE
rs309354	A	G	A	G	-0.0185	-0.1237	0.04632	0.05333	FALSE	FALSE	FALSE	ieu-a-31	6	31539010	0.0383	34652	0.00123	Inflamma	Inflamma	Inflamma	TRUE	igd	31539010	0.00418	9.8E-06	6	89714	ukb-d-22	|| id:u	TRUE	reported	igd	2	TRUE
rs310653	A	C	A	C	0.01211	-0.0254	0.10649	0.10563	FALSE	FALSE	FALSE	ieu-a-31	8	131259140	0.0289	34652	0.3789	Inflamma	Inflamma	Inflamma	TRUE	igd	131259140	0.00284	2E-05	8	89714	ukb-d-22	|| id:u	TRUE	reported	igd	2	TRUE
rs353857	T	A	T	A	0.00794	-0.0193	0.63465	0.62514	FALSE	TRUE	FALSE	ieu-a-31	3	196071832	0.0179	34652	0.2814	Inflamma	Inflamma	Inflamma	TRUE	igd	196071832	0.00183	1.4E-05	3	89714	ukb-d-22	|| id:u	TRUE	reported	igd	2	TRUE
rs373969	A	G	A	G	0.00727	-0.0277	0.49641	0.48877	FALSE	FALSE	FALSE	ieu-a-31	9	114798940	0.0187	34652	0.1376	Inflamma	Inflamma	Inflamma	TRUE	igd	114798940	0.00177	3.9E-05	9	89714	ukb-d-22	|| id:u	TRUE	reported	igd	2	TRUE
rs381770	G	C	G	C	-0.0072	0.0111	0.57626	0.57963	FALSE	TRUE	TRUE	ieu-a-31	8	2054195	0.017	34652	0.5155	Inflamma	Inflamma	Inflamma	TRUE	igd	2054195	0.00178	4.9E-05	8	89714	ukb-d-22	|| id:u	TRUE	reported	igd	2	FALSE
rs382594	A	G	A	G	0.00829	0.0162	0.25777	0.25249	FALSE	FALSE	FALSE	ieu-a-31	15	74317362	0.0193	34652	0.4004	Inflamma	Inflamma	Inflamma	TRUE	igd	74317362	0.00202	3.9E-05	15	89714	ukb-d-22	|| id:u	TRUE	reported	igd	2	TRUE
rs389792	T	G	T	G	-0.0075	0.0127	0.431	0.45112	FALSE	FALSE	FALSE	ieu-a-31	3	101703056	0.0169	34652	0.4521	Inflamma	Inflamma	Inflamma	TRUE	igd	101703056	0.00177	2.1E-05	3	89714	ukb-d-22	|| id:u	TRUE	reported	igd	2	TRUE
rs413194	T	G	T	G	-0.0074	0.0023	0.51371	0.50837	FALSE	FALSE	FALSE	ieu-a-31	16	54862861	0.0175	34652	0.8944	Inflamma	Inflamma	Inflamma	TRUE	igd	54862861	0.00178	3.1E-05	16	89714	ukb-d-22	|| id:u	TRUE	reported	igd	2	TRUE
rs443395	T	A	T	A	0.0078	-0.0059	0.43142	0.42551	FALSE	TRUE	TRUE	ieu-a-31	2	238115406	0.0175	34652	0.736	Inflamma	Inflamma	Inflamma	TRUE	igd	238114802	0.00182	1.7E-05	2	89714	ukb-d-22	|| id:u	TRUE	reported	igd	2	FALSE
rs468829	T	C	T	C	0.01399	-0.0286	0.07414	0.07418	FALSE	FALSE	FALSE	ieu-a-31	3	62460883	0.0333	34652	0.3898	Inflamma	Inflamma	Inflamma	TRUE	igd	62460883	0.00341	4E-05	3	89714	ukb-d-22	|| id:u	TRUE	reported	igd	2	TRUE
rs481445	C	T	C	T	-0.0168	0.01	0.94596	0.94186	FALSE	FALSE	FALSE	ieu-a-31	20	16178198	0.036	34652	0.7801	Inflamma	Inflamma	Inflamma	TRUE	igd	16178198	0.00387	1.5E-05	20	89714	ukb-d-22	|| id:u	TRUE	reported	igd	2	TRUE
rs497501	A	G	A	G	0.00903	-0.0014	0.22088	0.218	FALSE	FALSE	FALSE	ieu-a-31	4	39423512	0.0214	34652	0.949	Inflamma	Inflamma	Inflamma	TRUE	igd	39423512	0.00213	2.2E-05	4	89714	ukb-d-22	|| id:u	TRUE	reported	igd	2	TRUE
rs513564	T	C	T	C	0.00846	0.037	0.24995	0.23949	FALSE	FALSE	FALSE	ieu-a-31	10	99780908	0.0209	34652	0.07602	Inflamma	Inflamma	Inflamma	TRUE	igd	99780908	0.00207	4.4E-05	10	89714	ukb-d-22	|| id:u	TRUE	reported	igd	2	TRUE
rs554912	C	G	C	G	-0.0124	-0.0539	0.10323	0.1024	FALSE	TRUE	FALSE	ieu-a-31	1	226737915	0.0287	34652	0.06039	Inflamma	Inflamma	Inflamma	TRUE	igd	226735908	0.00295	2.8E-05	1	89714	ukb-d-22	|| id:u	TRUE	reported	igd	2	TRUE
rs558136	T	C	T	C	0.03104	-0.1652	0.01488	0.01648	FALSE	FALSE	FALSE	ieu-a-31	8	62318022	0.0692	34652	0.01697	Inflamma	Inflamma	Inflamma	TRUE	igd	62318022	0.00726	1.9E-05	8	89714	ukb-d-22	|| id:u	TRUE	reported	igd	2	TRUE
rs559280	A	G	A	G	-0.026	-0.0239	0.02501	0.01997	FALSE	FALSE	FALSE	ieu-a-31	16	69956730	0.0653	34652	0.7147	Inflamma	Inflamma	Inflamma	TRUE	igd	69750910	0.00566	4.2E-06	16	89714	ukb-d-22	|| id:u	TRUE	reported	igd	2	TRUE
rs561694	T	C	T	C	-0.0141	0.0334	0.07356	0.06647	FALSE	FALSE	FALSE	ieu-a-31	12	117155472	0.0405	34652	0.4096	Inflamma	Inflamma	Inflamma	TRUE	igd	117155472	0.00347	4.9E-05	12	89714	ukb-d-22	|| id:u	TRUE	reported	igd	2	TRUE
rs576053	C	G	C	G	-0.0218	-0.0837	0.02771	0.03405	FALSE	TRUE	FALSE	ieu-a-31	6	151835770	0.0508	34652	0.09955	Inflamma	Inflamma	Inflamma	TRUE	igd	151835770	0.00534	4.6E-05	6	89714	ukb-d-22	|| id:u	TRUE	reported	igd	2	TRUE
rs611254	T	C	T	C	-0.0151	-0.0721	0.08847	0.08521	FALSE	FALSE	FALSE	ieu-a-31	20	19727485	0.0302	34652	0.01687	Inflamma	Inflamma	Inflamma	TRUE	igd	19727485	0.00308	8.6E-07	20	89714	ukb-d-22	|| id:u	TRUE	reported	igd	2	TRUE
rs613060	C	G	C	G	-0.0103	-0.053	0.19003	0.19051	FALSE	TRUE	FALSE	ieu-a-31	20	43033095	0.0225	34652	0.01859	Inflamma	Inflamma	Inflamma	TRUE	igd	43033095	0.00223	3.8E-06	20	89714	ukb-d-22	|| id:u	TRUE	reported	igd	2	TRUE
rs620553	G	T	G	T	-0.014	-0.0013	0.08724	0.07426	FALSE	FALSE	FALSE	ieu-a-31	16	30187673	0.0378	34652	0.9735	Inflamma	Inflamma	Inflamma	TRUE	igd	30187673	0.00321	1.4E-05	16	89714	ukb-d-22	|| id:u	TRUE	reported	igd	2	TRUE
rs622080	G	A	G	A	0.02549	0.0266	0.02231	0.021	FALSE	FALSE	FALSE	ieu-a-31	20	62043227	0.0682	34652	0.6962	Inflamma	Inflamma	Inflamma	TRUE	igd	62043227	0.00592	1.6E-05	20	89714	ukb-d-22	|| id:u	TRUE	reported	igd	2	TRUE
rs624773	T	A	T	A	0.02016	-0.0241	0.04486	0.03763	FALSE	TRUE	FALSE	ieu-a-31	7	114935274	0.0464	34652	0.6041	Inflamma	Inflamma	Inflamma	TRUE	igd	114935274	0.00426	2.2E-06	7	89714	ukb-d-22	|| id:u	TRUE	reported	igd	2	TRUE
rs624961	T	C	T	C	0.02838	0.0038	0.02176	0.01975	FALSE	FALSE	FALSE	ieu-a-31	8	9288470	0.0763	34652	0.9603	Inflamma	Inflamma	Inflamma	TRUE	igd	9288470	0.00632	7.2E-06	8	89714	ukb-d-22	|| id:u	TRUE	reported	igd	2	TRUE
rs688129	C	T	C	T	0.00745	0.0342	0.58387	0.58535	FALSE	FALSE	FALSE	ieu-a-31	5	11147494	0.0169	34652	0.043	Inflamma	Inflamma	Inflamma	TRUE	igd	11147494	0.00177	2.7E-05	5	89714	ukb-d-22	|| id:u	TRUE	reported	igd	2	TRUE
rs689896	G	C	G	C	0.00762	0.0035	0.63769	0.61812	FALSE	TRUE	FALSE	ieu-a-31	18	74078911	0.019	34652	0.8526	Inflamma	Inflamma	Inflamma	TRUE	igd	74078911	0.00182	2.8E-05	18	89714	ukb-d-22	|| id:u	TRUE	reported	igd	2	TRUE
rs691490	C	A	C	A	0.01026	0.02	0.14903	0.13874	FALSE	FALSE	FALSE	ieu-a-31	6	139021524	0.025	34652	0.4238	Inflamma	Inflamma	Inflamma	TRUE	igd	139021524	0.00249	3.7E-05	6	89714	ukb-d-22	|| id:u	TRUE	reported	igd	2	TRUE
rs708246	A	G	A	G	-0.0085	-0.0312	0.67891	0.67028	FALSE	FALSE	FALSE	ieu-a-31	10	50486727	0.0182	34652	0.08545	Inflamma	Inflamma	Inflamma	TRUE	igd	50486727	0.00188	6.2E-06	10	89714	ukb-d-22	|| id:u	TRUE	reported	igd	2	TRUE
rs720454	G	C	G	C	0.00922	-0.0165	0.69183	0.69477	FALSE	TRUE	FALSE	ieu-a-31	16	718514	0.02	34652	0.411	Inflamma	Inflamma	Inflamma	TRUE	igd	718514	0.00197	2.8E-06	16	89714	ukb-d-22	|| id:u	TRUE	reported	igd	2	TRUE
rs726791	A	G	A	G	0.01238	0.0045	0.09252	0.0919	FALSE	FALSE	FALSE	ieu-a-31	1	66625369	0.0292	34652	0.8768	Inflamma	Inflamma	Inflamma	TRUE	igd	66625369	0.00303	4.5E-05	1	89714	ukb-d-22	|| id:u	TRUE	reported	igd	2	TRUE
rs727260	G	C	G	C	-0.0115	-0.031	0.1251	0.11126	FALSE	TRUE	FALSE	ieu-a-31	9	38072104	0.0274	34652	0.2578	Inflamma	Inflamma	Inflamma	TRUE	igd	38072104	0.00267	1.5E-05	9	89714	ukb-d-22	|| id:u	TRUE	reported	igd	2	TRUE
rs727603	C	T	C	T	0.03144	0.0123	0.01409	0.01437	FALSE	FALSE	FALSE	ieu-a-31	15	84192374	0.0711	34652	0.8627	Inflamma	Inflamma	Inflamma	TRUE	igd	84192374	0.00758	3.3E-05	15	89714	ukb-d-22	|| id:u	TRUE	reported	igd	2	TRUE
rs728961	A	G	A	G	0.0118	0.0354	0.1081	0.09229	FALSE	FALSE	FALSE	ieu-a-31	11	5845481	0.0348	34652	0.31	Inflamma	Inflamma	Inflamma	TRUE	igd	5845481	0.00291	5E-05	11	89714	ukb-d-22	|| id:u	TRUE	reported	igd	2	TRUE
rs732141	C	A	C	A	-0.0088	-0.0381	0.24763	0.2314	FALSE	FALSE	FALSE	ieu-a-31	12	121476099	0.0207	34652	0.06521	Inflamma	Inflamma	Inflamma	TRUE	igd	121476099	0.00203	1.7E-05	12	89714	ukb-d-22	|| id:u	TRUE	reported	igd	2	TRUE
rs733601	C	T	C	T	-0.0085	-0.0176	0.25051	0.22514	FALSE	FALSE	FALSE	ieu-a-31	21	41403037	0.021	34652	0.4026	Inflamma	Inflamma	Inflamma	TRUE	igd	41403037	0.00203	3E-05	21	89714	ukb-d-22	|| id:u	TRUE	reported	igd	2	TRUE
rs733619	C	T	C	T	-0.0073	0.0075	0.44239	0.43737	FALSE	FALSE	FALSE	ieu-a-31	13	53911048	0.0174	34652	0.6682	Inflamma	Inflamma	Inflamma	TRUE	igd	53911048	0.00178	4.3E-05	13	89714	ukb-d-22	|| id:u	TRUE	reported	igd	2	TRUE
rs744051	A	G	A	G	-0.0229	-0.035	0.02571	0.02345	FALSE	FALSE	FALSE	ieu-a-31	2	208711524	0.0636	34652	0.5817	Inflamma	Inflamma	Inflamma	TRUE	igd	208711524	0.00563	5E-05	2	89714	ukb-d-22	|| id:u	TRUE	reported	igd	2	TRUE
rs746397	T	A	T	A	0.01254	-0.0175	0.08923	0.089	FALSE	TRUE	FALSE	ieu-a-31	2	144885367	0.0327	34652	0.5926	Inflamma	Inflamma	Inflamma	TRUE	igd	144885367	0.00307	4.5E-05	2	89714	ukb-d-22	|| id:u	TRUE	reported	igd	2	TRUE
rs762178	G	A	G	A	0.04401	-0.0074	0.00963	0.009	FALSE	FALSE	FALSE	ieu-a-31	8	94600393	0.1254	34652	0.9527	Inflamma	Inflamma	Inflamma	TRUE	igd	94600393	0.00898	9.5E-07	8	89714	ukb-d-22	|| id:u	TRUE	reported	igd	2	TRUE
rs768794	T	C	T	C	-0.0227	-0.0665	0.02696	0.01345	FALSE	FALSE	FALSE	ieu-a-31	10	87990995	0.0894	34652	0.4571	Inflamma	Inflamma	Inflamma	TRUE	igd	87990995	0.00541	2.8E-05	10	89714	ukb-d-22	|| id:u	TRUE	reported	igd	2	TRUE
rs772112	A	T	A	T	0.01135	-0.013	0.86874	0.87237	FALSE	TRUE	FALSE	ieu-a-31	17	33621127	0.0296	34652	0.6612	Inflamma	Inflamma	Inflamma	TRUE	igd	33621127	0.00265	1.8E-05	17	89714	ukb-d-22	|| id:u	TRUE	reported	igd	2	TRUE
rs773097	A	C	A	C	0.02921	0.071	0.01555	0.01488	FALSE	FALSE	FALSE	ieu-a-31	7	73663178	0.0725	34652	0.3274	Inflamma	Inflamma	Inflamma	TRUE	igd	73663178	0.00712	4.1E-05	7	89714	ukb-d-22	|| id:u	TRUE	reported	igd	2	TRUE
rs778055	T	G	T	G	0.01932	0.0105	0.05764	0.06149	FALSE	FALSE	FALSE	ieu-a-31	6	144834312	0.0373	34652	0.7793	Inflamma	Inflamma	Inflamma	TRUE	igd	144834312	0.0038	3.6E-07	6	89714	ukb-d-22	|| id:u	TRUE	reported	igd	2	TRUE
rs794986	G	A	G	A	-0.0277	-0.0804	0.02296	0.01826	FALSE	FALSE	FALSE	ieu-a-31	8	134855286	0.0752	34652	0.2853	Inflamma	Inflamma	Inflamma	TRUE	igd	134855286	0.00583	2E-06	8	89714	ukb-d-22	|| id:u	TRUE	reported	igd	2	TRUE
rs795195	C	T	C	T	-0.0218	0.04089	0.02895	0.023	FALSE	FALSE	FALSE	ieu-a-31	14	34720312	0.0638	34652	0.5218	Inflamma	Inflamma	Inflamma	TRUE	igd	34720312	0.00521	2.9E-05	14	89714	ukb-d-22	|| id:u	TRUE	reported	igd	2	TRUE
rs797808	C	T	C	T	0.02474	-0.1294	0.02269	0.02189	FALSE	FALSE	FALSE	ieu-a-31	7	104247958	0.0601	34652	0.03132	Inflamma	Inflamma	Inflamma	TRUE	igd	104247958	0.00593	3E-05	7	89714	ukb-d-22	|| id:u	TRUE	reported	igd	2	TRUE
rs800655	C	G	C	G	0.01243	0.021	0.15964	0.1816	FALSE	TRUE	FALSE	ieu-a-31	14	67294069	0.0226	34652	0.3522	Inflamma	Inflamma	Inflamma	TRUE	igd	67294069	0.00244	3.7E-07	14	89714	ukb-d-22	|| id:u	TRUE	reported	igd	2	TRUE
rs876462	G	T	G	T	-0.008	-0.0409	0.37434	0.36477	FALSE	FALSE	FALSE	ieu-a-31	2	37515880	0.0177	34652	0.02087	Inflamma	Inflamma	Inflamma	TRUE	igd	37515880	0.00181	1E-05	2	89714	ukb-d-22	|| id:u	TRUE	reported	igd	2	TRUE
rs884258	C	T	C	T	-0.0079	0.007	0.67693	0.67274	FALSE	FALSE	FALSE	ieu-a-31	9	107057177	0.0179	34652	0.6947	Inflamma	Inflamma	Inflamma	TRUE	igd	107057177	0.00189	3.3E-05	9	89714	ukb-d-22	|| id:u	TRUE	reported	igd	2	TRUE
rs914186	A	G	A	G	0.00773	0.0167	0.66006	0.6746	FALSE	FALSE	FALSE	ieu-a-31	21	42619657	0.0179	34652	0.3507	Inflamma	Inflamma	Inflamma	TRUE	igd	42619657	0.00186	3.3E-05	21	89714	ukb-d-22	|| id:u	TRUE	reported	igd	2	TRUE
rs936835	T	A	T	A	0.03017	0.2044	0.01474	0.02386	FALSE	TRUE	FALSE	ieu-a-31	6	21808192	0.0665	34652	0.00213	Inflamma	Inflamma	Inflamma	TRUE	igd	21808192	0.00733	3.9E-05	6	89714	ukb-d-22	|| id:u	TRUE	reported	igd	2	TRUE
rs953033	G	T	G	T	0.0077	-0.0278	0.65454	0.63877	FALSE	FALSE	FALSE	ieu-a-31	13	75070201	0.0175	34652	0.1111	Inflamma	Inflamma	Inflamma	TRUE	igd	75070201	0.00185	3.1E-05	13	89714	ukb-d-22	|| id:u	TRUE	reported	igd	2	TRUE

Table S8. Detail Information of the Genetic Variants in the MR Analysis of SHS and UC

SNP	effect_a	other_al	effect_a	other_al	beta.exp	beta.out	eaf.expo	eaf.outc	remove	palindro	ambiguou	id.outco	chr	pos	se.outco	samplesi	pval.out	outcome	original	outcome.	mr_keep.	data_sou	pos.exposu	se.expos	pval.exp	chr.expo	samplesi	id.expos	exposure	mr_keep.	pval_ori	data_sou	action	mr_keep
rs100090	A	G	A	G	-0.0076	-0.0275	0.53599	0.53398	FALSE	FALSE	FALSE	ieu-a-32	4	180336832	0.0213	27432	0.1975	Ulcerati	Ulcerati	Ulcerati	TRUE	igd	180336832	0.00176	1.4E-05	4	89714	ukb-d-22	|| id:u	TRUE	reported	igd	2	TRUE
rs102193	T	C	T	C	-0.0095	0.0344	0.1902	0.19625	FALSE	FALSE	FALSE	ieu-a-32	11	86339724	0.0263	27432	0.1917	Ulcerati	Ulcerati	Ulcerati	TRUE	igd	86339724	0.00224	2.4E-05	11	89714	ukb-d-22	|| id:u	TRUE	reported	igd	2	TRUE
rs104255	C	T	C	T	-0.0084	0.0279	0.70783	0.69449	FALSE	FALSE	FALSE	ieu-a-32	19	836043	0.0336	27432	0.4069	Ulcerati	Ulcerati	Ulcerati	TRUE	igd	836043	0.00204	3.6E-05	19	89714	ukb-d-22	|| id:u	TRUE	reported	igd	2	TRUE
rs109451	A	T	A	T	-0.0079	0.0082	0.31369	0.301	FALSE	TRUE	FALSE	ieu-a-32	6	69354524	0.023	27432	0.7199	Ulcerati	Ulcerati	Ulcerati	TRUE	igd	69354524	0.00189	3.2E-05	6	89714	ukb-d-22	|| id:u	TRUE	reported	igd	2	TRUE
rs112169	A	G	A	G	0.03019	0.0097	0.01694	0.01062	FALSE	FALSE	FALSE	ieu-a-32	7	70641982	0.1262	27432	0.9386	Ulcerati	Ulcerati	Ulcerati	TRUE	igd	70641982	0.00676	8.1E-06	7	89714	ukb-d-22	|| id:u	TRUE	reported	igd	2	TRUE
rs114134	T	C	T	C	0.04033	0.1587	0.00945	0.01023	FALSE	FALSE	FALSE	ieu-a-32	6	41851676	0.1465	27432	0.2786	Ulcerati	Ulcerati	Ulcerati	TRUE	igd	41851676	0.00973	3.4E-05	6	89714	ukb-d-22	|| id:u	TRUE	reported	igd	2	TRUE
rs116249	T	C	T	C	-0.0289	-0.1731	0.01801	0.01844	FALSE	FALSE	FALSE	ieu-a-32	3	190482826	0.1029	27432	0.09271	Ulcerati	Ulcerati	Ulcerati	TRUE	igd	190482826	0.00697	3.5E-05	3	89714	ukb-d-22	|| id:u	TRUE	reported	igd	2	TRUE
rs117410	G	A	G	A	-0.0415	0.0638	0.00888	0.01075	FALSE	FALSE	FALSE	ieu-a-32	15	47979695	0.1102	27432	0.5628	Ulcerati	Ulcerati	Ulcerati	TRUE	igd	47979695	0.00945	1.1E-05	15	89714	ukb-d-22	|| id:u	TRUE	reported	igd	2	TRUE
rs117514	T	G	T	G	0.00909	-0.019	0.18988	0.18622	FALSE	FALSE	FALSE	ieu-a-32	22	49534383	0.0291	27432	0.5142	Ulcerati	Ulcerati	Ulcerati	TRUE	igd	49534383	0.00223	4.5E-05	22	89714	ukb-d-22	|| id:u	TRUE	reported	igd	2	TRUE
rs117858	A	G	A	G	-0.0212	0.0884	0.03627	0.0392	FALSE	FALSE	FALSE	ieu-a-32	6	149289638	0.061	27432	0.1473	Ulcerati	Ulcerati	Ulcerati	TRUE	igd	149289638	0.00484	1.2E-05	6	89714	ukb-d-22	|| id:u	TRUE	reported	igd	2	TRUE
rs118098	T	C	T	C	0.02983	0.1037	0.02017	0.01588	FALSE	FALSE	FALSE	ieu-a-32	21	33944718	0.1039	27432	0.3181	Ulcerati	Ulcerati	Ulcerati	TRUE	igd	33944718	0.00656	5.4E-06	21	89714	ukb-d-22	|| id:u	TRUE	reported	igd	2	TRUE
rs118146	G	A	G	A	-0.0131	-0.0438	0.09386	0.07673	FALSE	FALSE	FALSE	ieu-a-32	8	22116219	0.0488	27432	0.3689	Ulcerati	Ulcerati	Ulcerati	TRUE	igd	22116219	0.00312	2.5E-05	8	89714	ukb-d-22	|| id:u	TRUE	reported	igd	2	TRUE
rs120418	T	C	T	C	0.01394	0.002	0.08023	0.08281	FALSE	FALSE	FALSE	ieu-a-32	1	60484531	0.0395	27432	0.9599	Ulcerati	Ulcerati	Ulcerati	TRUE	igd	60484531	0.00324	1.7E-05	1	89714	ukb-d-22	|| id:u	TRUE	reported	igd	2	TRUE
rs121057	G	T	G	T	0.00874	-0.0151	0.36894	0.38373	FALSE	FALSE	FALSE	ieu-a-32	2	60736985	0.0227	27432	0.5047	Ulcerati	Ulcerati	Ulcerati	TRUE	igd	60736985	0.00184	2E-06	2	89714	ukb-d-22	|| id:u	TRUE	reported	igd	2	TRUE
rs124091	C	T	C	T	-0.0085	-0.0353	0.24869	0.25671	FALSE	FALSE	FALSE	ieu-a-32	1	114055488	0.0252	27432	0.1619	Ulcerati	Ulcerati	Ulcerati	TRUE	igd	114055488	0.00208	3.9E-05	1	89714	ukb-d-22	|| id:u	TRUE	reported	igd	2	TRUE
rs124840	T	C	T	C	0.00905	0.0842	0.20587	0.20033	FALSE	FALSE	FALSE	ieu-a-32	22	51099862	0.0267	27432	0.00157	Ulcerati	Ulcerati	Ulcerati	TRUE	igd	51099862	0.00217	3E-05	22	89714	ukb-d-22	|| id:u	TRUE	reported	igd	2	TRUE
rs124932	G	T	G	T	0.01626	-0.0719	0.05371	0.05373	FALSE	FALSE	FALSE	ieu-a-32	3	16679114	0.0501	27432	0.151	Ulcerati	Ulcerati	Ulcerati	TRUE	igd	16679114	0.00388	2.8E-05	3	89714	ukb-d-22	|| id:u	TRUE	reported	igd	2	TRUE
rs126292	A	T	A	T	-0.0081	-0.0068	0.33223	0.32524	FALSE	TRUE	FALSE	ieu-a-32	6	16806082	0.0236	27432	0.7743	Ulcerati	Ulcerati	Ulcerati	TRUE	igd	16806082	0.00189	1.7E-05	6	89714	ukb-d-22	|| id:u	TRUE	reported	igd	2	TRUE
rs127653	T	C	T	C	0.00867	-0.0102	0.23241	0.23327	FALSE	FALSE	FALSE	ieu-a-32	10	7631014	0.0251	27432	0.6854	Ulcerati	Ulcerati	Ulcerati	TRUE	igd	7631014	0.0021	3.7E-05	10	89714	ukb-d-22	|| id:u	TRUE	reported	igd	2	TRUE
rs128669	A	G	A	G	0.02524	0.124	0.02351	0.02349	FALSE	FALSE	FALSE	ieu-a-32	13	110066103	0.0684	27432	0.06991	Ulcerati	Ulcerati	Ulcerati	TRUE	igd	110066103	0.00579	1.3E-05	13	89714	ukb-d-22	|| id:u	TRUE	reported	igd	2	TRUE
rs138858	A	G	A	G	0.04308	-0.119	0.00736	0.0117	FALSE	FALSE	FALSE	ieu-a-32	15	79099986	0.1149	27432	0.3003	Ulcerati	Ulcerati	Ulcerati	TRUE	igd	79099986	0.01047	3.9E-05	15	89714	ukb-d-22	|| id:u	TRUE	reported	igd	2	TRUE
rs140647	G	A	G	A	0.04379	-0.006	0.0076	0.009	FALSE	FALSE	FALSE	ieu-a-32	19	20290042	0.1309	27432	0.9635	Ulcerati	Ulcerati	Ulcerati	TRUE	igd	20290042	0.01059	3.6E-05	19	89714	ukb-d-22	|| id:u	TRUE	reported	igd	2	TRUE
rs142563	A	G	A	G	-0.0344	0.1301	0.01473	0.01935	FALSE	FALSE	FALSE	ieu-a-32	13	23278334	0.0852	27432	0.1269	Ulcerati	Ulcerati	Ulcerati	TRUE	igd	23278334	0.00745	4E-06	13	89714	ukb-d-22	|| id:u	TRUE	reported	igd	2	TRUE
rs143255	C	T	C	T	0.01694	0.0187	0.04814	0.053	FALSE	FALSE	FALSE	ieu-a-32	7	147431844	0.0489	27432	0.7023	Ulcerati	Ulcerati	Ulcerati	TRUE	igd	147431844	0.00413	4.1E-05	7	89714	ukb-d-22	|| id:u	TRUE	reported	igd	2	TRUE
rs144479	A	G	A	G	0.05832	-0.0674	0.00476	0.00934	FALSE	FALSE	FALSE	ieu-a-32	3	122728248	0.1564	27432	0.6667	Ulcerati	Ulcerati	Ulcerati	TRUE	igd	122728248	0.01297	6.9E-06	3	89714	ukb-d-22	|| id:u	TRUE	reported	igd	2	TRUE
rs145817	T	C	T	C	0.00807	0.0202	0.54956	0.56827	FALSE	FALSE	FALSE	ieu-a-32	12	41777144	0.0214	27432	0.3452	Ulcerati	Ulcerati	Ulcerati	TRUE	igd	41777144	0.00176	4.4E-06	12	89714	ukb-d-22	|| id:u	TRUE	reported	igd	2	TRUE
rs147567	A	G	A	G	0.01947	-0.0672	0.03983	0.04119	FALSE	FALSE	FALSE	ieu-a-32	7	47544762	0.064	27432	0.2937	Ulcerati	Ulcerati	Ulcerati	TRUE	igd	47544762	0.0047	3.5E-05	7	89714	ukb-d-22	|| id:u	TRUE	reported	igd	2	TRUE
rs148904	C	G	C	G	0.03265	-0.1153	0.01511	0.01685	FALSE	TRUE	FALSE	ieu-a-32	22	25249949	0.1032	27432	0.2638	Ulcerati	Ulcerati	Ulcerati	TRUE	igd	25249949	0.0076	1.7E-05	22	89714	ukb-d-22	|| id:u	TRUE	reported	igd	2	TRUE
rs149651	G	C	G	C	0.03813	0.22321	0.01176	0.01151	FALSE	TRUE	FALSE	ieu-a-32	6	160595479	0.1021	27432	0.02874	Ulcerati	Ulcerati	Ulcerati	TRUE	igd	160595479	0.00836	5E-06	6	89714	ukb-d-22	|| id:u	TRUE	reported	igd	2	TRUE
rs149772	T	A	T	A	0.0282	0.0015	0.02041	0.02125	FALSE	TRUE	FALSE	ieu-a-32	4	12576228	0.0899	27432	0.9864	Ulcerati	Ulcerati	Ulcerati	TRUE	igd	12576228	0.00662	2E-05	4	89714	ukb-d-22	|| id:u	TRUE	reported	igd	2	TRUE
rs150100	C	T	C	T	0.02882	-0.0674	0.02023	0.02249	FALSE	FALSE	FALSE	ieu-a-32	3	96018959	0.0715	27432	0.346	Ulcerati	Ulcerati	Ulcerati	TRUE	igd	96018959	0.00671	1.8E-05	3	89714	ukb-d-22	|| id:u	TRUE	reported	igd	2	TRUE
rs150689	A	G	A	G	0.03224	-0.0117	0.0145	0.01415	FALSE	FALSE	FALSE	ieu-a-32	13	23446586	0.1106	27432	0.9158	Ulcerati	Ulcerati	Ulcerati	TRUE	igd	23446586	0.00765	2.5E-05	13	89714	ukb-d-22	|| id:u	TRUE	reported	igd	2	TRUE
rs171494	G	A	G	A	0.01389	-0.0629	0.08945	0.10398	FALSE	FALSE	FALSE	ieu-a-32	2	235265207	0.0388	27432	0.1053	Ulcerati	Ulcerati	Ulcerati	TRUE	igd	235265207	0.00314	9.5E-06	2	89714	ukb-d-22	|| id:u	TRUE	reported	igd	2	TRUE
rs176026	A	G	A	G	0.03352	-0.0324	0.01586	0.01707	FALSE	FALSE	FALSE	ieu-a-32	12	42480604	0.0849	27432	0.7032	Ulcerati	Ulcerati	Ulcerati	TRUE	igd	42480604	0.00714	2.7E-06	12	89714	ukb-d-22	|| id:u	TRUE	reported	igd	2	TRUE
rs189879	T	G	T	G	0.02926	0.0362	0.01658	0.00666	FALSE	FALSE	FALSE	ieu-a-32	4	69058096	0.1711	27432	0.8324	Ulcerati	Ulcerati	Ulcerati	TRUE	igd	69058096	0.00705	3.3E-05	4	89714	ukb-d-22	|| id:u	TRUE	reported	igd	2	TRUE
rs192230	A	G	A	G	0.09947	-0.1222	0.00159	0.01015	FALSE	FALSE	FALSE	ieu-a-32	19	50188478	0.1523	27432	0.4223	Ulcerati	Ulcerati	Ulcerati	TRUE	igd	50188478	0.02342	2.2E-05	19	89714	ukb-d-22	|| id:u	TRUE	reported	igd	2	TRUE
rs192463	T	A	T	A	0.02663	-0.0027	0.01955	0.01275	FALSE	TRUE	FALSE	ieu-a-32	2	213744121	0.1026	27432	0.9789	Ulcerati	Ulcerati	Ulcerati	TRUE	igd	213744121	0.00644	3.6E-05	2	89714	ukb-d-22	|| id:u	TRUE	reported	igd	2	TRUE
rs199341	G	C	G	C	0.0083	0.0508	0.25339	0.26502	FALSE	TRUE	FALSE	ieu-a-32	10	63400853	0.0239	27432	0.03328	Ulcerati	Ulcerati	Ulcerati	TRUE	igd	63400853	0.00202	3.9E-05	10	89714	ukb-d-22	|| id:u	TRUE	reported	igd	2	TRUE
rs203146	C	T	C	T	0.00758	-0.0051	0.62391	0.62151	FALSE	FALSE	FALSE	ieu-a-32	10	131923448	0.0225	27432	0.8198	Ulcerati	Ulcerati	Ulcerati	TRUE	igd	131923448	0.00181	2.9E-05	10	89714	ukb-d-22	|| id:u	TRUE	reported	igd	2	TRUE
rs206940	G	A	G	A	-0.0084	-0.0047	0.34093	0.32649	FALSE	FALSE	FALSE	ieu-a-32	12	56364321	0.0224	27432	0.8343	Ulcerati	Ulcerati	Ulcerati	TRUE	igd	56364321	0.00185	5.7E-06	12	89714	ukb-d-22	|| id:u	TRUE	reported	igd	2	TRUE
rs207008	G	T	G	T	-0.0196	0.0081	0.94188	0.93905	FALSE	FALSE	FALSE	ieu-a-32	21	18735936	0.0453	27432	0.8588	Ulcerati	Ulcerati	Ulcerati	TRUE	igd	18735936	0.00375	1.8E-07	21	89714	ukb-d-22	|| id:u	TRUE	reported	igd	2	TRUE
rs229649	G	A	G	A	-0.0113	-0.0082	0.11402	0.12	FALSE	FALSE	FALSE	ieu-a-32	13	95096111	0.0325	27432	0.8004	Ulcerati	Ulcerati	Ulcerati	TRUE	igd	95096111	0.00277	4.3E-05	13	89714	ukb-d-22	|| id:u	TRUE	reported	igd	2	TRUE
rs268312	C	A	C	A	-0.0072	0.0273	0.46584	0.463	FALSE	FALSE	FALSE	ieu-a-32	15	36024804	0.0218	27432	0.2113	Ulcerati	Ulcerati	Ulcerati	TRUE	igd	36024804	0.00177	4.9E-05	15	89714	ukb-d-22	|| id:u	TRUE	reported	igd	2	TRUE
rs276583	A	G	A	G	0.01277	0.0299	0.12036	0.12202	FALSE	FALSE	FALSE	ieu-a-32	10	21034317	0.0335	27432	0.3711	Ulcerati	Ulcerati	Ulcerati	TRUE	igd	21034317	0.00272	2.7E-06	10	89714	ukb-d-22	|| id:u	TRUE	reported	igd	2	TRUE
rs284717	T	C	T	C	0.00882	0.0135	0.63416	0.64251	FALSE	FALSE	FALSE	ieu-a-32	11	105877624	0.0221	27432	0.543	Ulcerati	Ulcerati	Ulcerati	TRUE	igd	105877624	0.00183	1.4E-06	11	89714	ukb-d-22	|| id:u	TRUE	reported	igd	2	TRUE
rs309354	A	G	A	G	-0.0185	-0.1522	0.04632	0.05345	FALSE	FALSE	FALSE	ieu-a-32	6	31539010	0.0491	27432	0.00195	Ulcerati	Ulcerati	Ulcerati	TRUE	igd	31539010	0.00418	9.8E-06	6	89714	ukb-d-22	|| id:u	TRUE	reported	igd	2	TRUE
rs310653	A	C	A	C	0.01211	0.0151	0.10649	0.10827	FALSE	FALSE	FALSE	ieu-a-32	8	131259140	0.0349	27432	0.6651	Ulcerati	Ulcerati	Ulcerati	TRUE	igd	131259140	0.00284	2E-05	8	89714	ukb-d-22	|| id:u	TRUE	reported	igd	2	TRUE
rs353857	T	A	T	A	0.00794	-0.025	0.63465	0.62848	FALSE	TRUE	FALSE	ieu-a-32	3	196071832	0.0227	27432	0.2698	Ulcerati	Ulcerati	Ulcerati	TRUE	igd	196071832	0.00183	1.4E-05	3	89714	ukb-d-22	|| id:u	TRUE	reported	igd	2	TRUE
rs373969	A	G	A	G	0.00727	0.0059	0.49641	0.49249	FALSE	FALSE	FALSE	ieu-a-32	9	114798940	0.0232	27432	0.7983	Ulcerati	Ulcerati	Ulcerati	TRUE	igd	114798940	0.00177	3.9E-05	9	89714	ukb-d-22	|| id:u	TRUE	reported	igd	2	TRUE
rs381770	G	C	G	C	-0.0072	0.0263	0.57626	0.578	FALSE	TRUE	TRUE	ieu-a-32	8	2054195	0.0215	27432	0.2207	Ulcerati	Ulcerati	Ulcerati	TRUE	igd	2054195	0.00178	4.9E-05	8	89714	ukb-d-22	|| id:u	TRUE	reported	igd	2	FALSE
rs382594	A	G	A	G	0.00829	0.0243	0.25777	0.25103	FALSE	FALSE	FALSE	ieu-a-32	15	74317362	0.0242	27432	0.3162	Ulcerati	Ulcerati	Ulcerati	TRUE	igd	74317362	0.00202	3.9E-05	15	89714	ukb-d-22	|| id:u	TRUE	reported	igd	2	TRUE
rs389792	T	G	T	G	-0.0075	0.0227	0.431	0.44998	FALSE	FALSE	FALSE	ieu-a-32	3	101703056	0.0213	27432	0.2875	Ulcerati	Ulcerati	Ulcerati	TRUE	igd	101703056	0.00177	2.1E-05	3	89714	ukb-d-22	|| id:u	TRUE	reported	igd	2	TRUE
rs413194	T	G	T	G	-0.0074	0.0058	0.51371	0.51078	FALSE	FALSE	FALSE	ieu-a-32	16	54862861	0.0223	27432	0.795	Ulcerati	Ulcerati	Ulcerati	TRUE	igd	54862861	0.00178	3.1E-05	16	89714	ukb-d-22	|| id:u	TRUE	reported	igd	2	TRUE
rs443395	T	A	T	A	0.0078	0.0093	0.43142	0.42698	FALSE	TRUE	TRUE	ieu-a-32	2	238115406	0.022	27432	0.6718	Ulcerati	Ulcerati	Ulcerati	TRUE	igd	238114802	0.00182	1.7E-05	2	89714	ukb-d-22	|| id:u	TRUE	reported	igd	2	FALSE
rs468829	T	C	T	C	0.01399	0.0007	0.07414	0.07357	FALSE	FALSE	FALSE	ieu-a-32	3	62460883	0.042	27432	0.9871	Ulcerati	Ulcerati	Ulcerati	TRUE	igd	62460883	0.00341	4E-05	3	89714	ukb-d-22	|| id:u	TRUE	reported	igd	2	TRUE
rs481445	C	T	C	T	-0.0168	-0.0281	0.94596	0.9413	FALSE	FALSE	FALSE	ieu-a-32	20	16178198	0.0451	27432	0.5327	Ulcerati	Ulcerati	Ulcerati	TRUE	igd	16178198	0.00387	1.5E-05	20	89714	ukb-d-22	|| id:u	TRUE	reported	igd	2	TRUE
rs497501	A	G	A	G	0.00903	-0.0165	0.22088	0.21724	FALSE	FALSE	FALSE	ieu-a-32	4	39423512	0.0272	27432	0.5429	Ulcerati	Ulcerati	Ulcerati	TRUE	igd	39423512	0.00213	2.2E-05	4	89714	ukb-d-22	|| id:u	TRUE	reported	igd	2	TRUE
rs513564	T	C	T	C	0.00846	0.006	0.24995	0.23951	FALSE	FALSE	FALSE	ieu-a-32	10	99780908	0.0263	27432	0.8184	Ulcerati	Ulcerati	Ulcerati	TRUE	igd	99780908	0.00207	4.4E-05	10	89714	ukb-d-22	|| id:u	TRUE	reported	igd	2	TRUE
rs554912	C	G	C	G	-0.0124	-0.0385	0.10323	0.10475	FALSE	TRUE	FALSE	ieu-a-32	1	226737915	0.0358	27432	0.2823	Ulcerati	Ulcerati	Ulcerati	TRUE	igd	226735908	0.00295	2.8E-05	1	89714	ukb-d-22	|| id:u	TRUE	reported	igd	2	TRUE
rs558136	T	C	T	C	0.03104	-0.1447	0.01488	0.01664	FALSE	FALSE	FALSE	ieu-a-32	8	62318022	0.0875	27432	0.09798	Ulcerati	Ulcerati	Ulcerati	TRUE	igd	62318022	0.00726	1.9E-05	8	89714	ukb-d-22	|| id:u	TRUE	reported	igd	2	TRUE
rs559280	A	G	A	G	-0.026	-0.0217	0.02501	0.02032	FALSE	FALSE	FALSE	ieu-a-32	16	69956730	0.0831	27432	0.7937	Ulcerati	Ulcerati	Ulcerati	TRUE	igd	69750910	0.00566	4.2E-06	16	89714	ukb-d-22	|| id:u	TRUE	reported	igd	2	TRUE
rs561694	T	C	T	C	-0.0141	0.0061	0.07356	0.06697	FALSE	FALSE	FALSE	ieu-a-32	12	117155472	0.0505	27432	0.9036	Ulcerati	Ulcerati	Ulcerati	TRUE	igd	117155472	0.00347	4.9E-05	12	89714	ukb-d-22	|| id:u	TRUE	reported	igd	2	TRUE
rs576053	C	G	C	G	-0.0218	-0.0581	0.02771	0.03409	FALSE	TRUE	FALSE	ieu-a-32	6	151835770	0.0644	27432	0.367	Ulcerati	Ulcerati	Ulcerati	TRUE	igd	151835770	0.00534	4.6E-05	6	89714	ukb-d-22	|| id:u	TRUE	reported	igd	2	TRUE
rs611254	T	C	T	C	-0.0151	-0.0881	0.08847	0.08559	FALSE	FALSE	FALSE	ieu-a-32	20	19727485	0.0383	27432	0.02149	Ulcerati	Ulcerati	Ulcerati	TRUE	igd	19727485	0.00308	8.6E-07	20	89714	ukb-d-22	|| id:u	TRUE	reported	igd	2	TRUE
rs613060	C	G	C	G	-0.0103	-0.0829	0.19003	0.19098	FALSE	TRUE	FALSE	ieu-a-32	20	43033095	0.0278	27432	0.00289	Ulcerati	Ulcerati	Ulcerati	TRUE	igd	43033095	0.00223	3.8E-06	20	89714	ukb-d-22	|| id:u	TRUE	reported	igd	2	TRUE
rs620553	G	T	G	T	-0.014	-0.0588	0.08724	0.07624	FALSE	FALSE	FALSE	ieu-a-32	16	30187673	0.0467	27432	0.2085	Ulcerati	Ulcerati	Ulcerati	TRUE	igd	30187673	0.00321	1.4E-05	16	89714	ukb-d-22	|| id:u	TRUE	reported	igd	2	TRUE
rs622080	G	A	G	A	0.02549	-0.0059	0.02231	0.02075	FALSE	FALSE	FALSE	ieu-a-32	20	62043227	0.0859	27432	0.945	Ulcerati	Ulcerati	Ulcerati	TRUE	igd	62043227	0.00592	1.6E-05	20	89714	ukb-d-22	|| id:u	TRUE	reported	igd	2	TRUE
rs624773	T	A	T	A	0.02016	-0.0098	0.04486	0.03825	FALSE	TRUE	FALSE	ieu-a-32	7	114935274	0.0571	27432	0.8633	Ulcerati	Ulcerati	Ulcerati	TRUE	igd	114935274	0.00426	2.2E-06	7	89714	ukb-d-22	|| id:u	TRUE	reported	igd	2	TRUE
rs624961	T	C	T	C	0.02838	0.0932	0.02176	0.02095	FALSE	FALSE	FALSE	ieu-a-32	8	9288470	0.0939	27432	0.3208	Ulcerati	Ulcerati	Ulcerati	TRUE	igd	9288470	0.00632	7.2E-06	8	89714	ukb-d-22	|| id:u	TRUE	reported	igd	2	TRUE
rs688129	C	T	C	T	0.00745	0.0298	0.58387	0.58429	FALSE	FALSE	FALSE	ieu-a-32	5	11147494	0.0213	27432	0.1617	Ulcerati	Ulcerati	Ulcerati	TRUE	igd	11147494	0.00177	2.7E-05	5	89714	ukb-d-22	|| id:u	TRUE	reported	igd	2	TRUE
rs689896	G	C	G	C	0.00762	-0.0104	0.63769	0.61651	FALSE	TRUE	FALSE	ieu-a-32	18	74078911	0.0239	27432	0.6625	Ulcerati	Ulcerati	Ulcerati	TRUE	igd	74078911	0.00182	2.8E-05	18	89714	ukb-d-22	|| id:u	TRUE	reported	igd	2	TRUE
rs691490	C	A	C	A	0.01026	0.01521	0.14903	0.13876	FALSE	FALSE	FALSE	ieu-a-32	6	139021524	0.0314	27432	0.6276	Ulcerati	Ulcerati	Ulcerati	TRUE	igd	139021524	0.00249	3.7E-05	6	89714	ukb-d-22	|| id:u	TRUE	reported	igd	2	TRUE
rs708246	A	G	A	G	-0.0085	-0.0329	0.67891	0.67095	FALSE	FALSE	FALSE	ieu-a-32	10	50486727	0.0228	27432	0.1489	Ulcerati	Ulcerati	Ulcerati	TRUE	igd	50486727	0.00188	6.2E-06	10	89714	ukb-d-22	|| id:u	TRUE	reported	igd	2	TRUE
rs720454	G	C	G	C	0.00922	0.0005	0.69183	0.69597	FALSE	TRUE	FALSE	ieu-a-32	16	718514	0.0251	27432	0.9846	Ulcerati	Ulcerati	Ulcerati	TRUE	igd	718514	0.00197	2.8E-06	16	89714	ukb-d-22	|| id:u	TRUE	reported	igd	2	TRUE
rs726791	A	G	A	G	0.01238	0.0322	0.09252	0.0942	FALSE	FALSE	FALSE	ieu-a-32	1	66625369	0.0362	27432	0.3743	Ulcerati	Ulcerati	Ulcerati	TRUE	igd	66625369	0.00303	4.5E-05	1	89714	ukb-d-22	|| id:u	TRUE	reported	igd	2	TRUE
rs727260	G	C	G	C	-0.0115	0.0232	0.1251	0.11176	FALSE	TRUE	FALSE	ieu-a-32	9	38072104	0.0341	27432	0.4964	Ulcerati	Ulcerati	Ulcerati	TRUE	igd	38072104	0.00267	1.5E-05	9	89714	ukb-d-22	|| id:u	TRUE	reported	igd	2	TRUE
rs727603	C	T	C	T	0.03144	-0.0076	0.01409	0.01451	FALSE	FALSE	FALSE	ieu-a-32	15	84192374	0.087	27432	0.93	Ulcerati	Ulcerati	Ulcerati	TRUE	igd	84192374	0.00758	3.3E-05	15	89714	ukb-d-22	|| id:u	TRUE	reported	igd	2	TRUE
rs728961	A	G	A	G	0.0118	-0.0059	0.1081	0.09239	FALSE	FALSE	FALSE	ieu-a-32	11	5845481	0.0434	27432	0.892	Ulcerati	Ulcerati	Ulcerati	TRUE	igd	5845481	0.00291	5E-05	11	89714	ukb-d-22	|| id:u	TRUE	reported	igd	2	TRUE
rs732141	C	A	C	A	-0.0088	-0.0474	0.24763	0.23273	FALSE	FALSE	FALSE	ieu-a-32	12	121476099	0.0259	27432	0.06711	Ulcerati	Ulcerati	Ulcerati	TRUE	igd	121476099	0.00203	1.7E-05	12	89714	ukb-d-22	|| id:u	TRUE	reported	igd	2	TRUE
rs733601	C	T	C	T	-0.0085	-0.0345	0.25051	0.22748	FALSE	FALSE	FALSE	ieu-a-32	21	41403037	0.0264	27432	0.1914	Ulcerati	Ulcerati	Ulcerati	TRUE	igd	41403037	0.00203	3E-05	21	89714	ukb-d-22	|| id:u	TRUE	reported	igd	2	TRUE
rs733619	C	T	C	T	-0.0073	-0.0119	0.44239	0.437	FALSE	FALSE	FALSE	ieu-a-32	13	53911048	0.0219	27432	0.5872	Ulcerati	Ulcerati	Ulcerati	TRUE	igd	53911048	0.00178	4.3E-05	13	89714	ukb-d-22	|| id:u	TRUE	reported	igd	2	TRUE
rs744051	A	G	A	G	-0.0229	0.0016	0.02571	0.02416	FALSE	FALSE	FALSE	ieu-a-32	2	208711524	0.0793	27432	0.9844	Ulcerati	Ulcerati	Ulcerati	TRUE	igd	208711524	0.00563	5E-05	2	89714	ukb-d-22	|| id:u	TRUE	reported	igd	2	TRUE
rs746397	T	A	T	A	0.01254	-0.0151	0.08923	0.088	FALSE	TRUE	FALSE	ieu-a-32	2	144885367	0.0419	27432	0.7193	Ulcerati	Ulcerati	Ulcerati	TRUE	igd	144885367	0.00307	4.5E-05	2	89714	ukb-d-22	|| id:u	TRUE	reported	igd	2	TRUE
rs762178	G	A	G	A	0.04401	0.12541	0.00963	0.00925	FALSE	FALSE	FALSE	ieu-a-32	8	94600393	0.1731	27432	0.469	Ulcerati	Ulcerati	Ulcerati	TRUE	igd	94600393	0.00898	9.5E-07	8	89714	ukb-d-22	|| id:u	TRUE	reported	igd	2	TRUE
rs768794	T	C	T	C	-0.0227	-0.0902	0.02696	0.01345	FALSE	FALSE	FALSE	ieu-a-32	10	87990995	0.1121	27432	0.4211	Ulcerati	Ulcerati	Ulcerati	TRUE	igd	87990995	0.00541	2.8E-05	10	89714	ukb-d-22	|| id:u	TRUE	reported	igd	2	TRUE
rs772112	A	T	A	T	0.01135	-0.0607	0.86874	0.87024	FALSE	TRUE	FALSE	ieu-a-32	17	33621127	0.0366	27432	0.0967	Ulcerati	Ulcerati	Ulcerati	TRUE	igd	33621127	0.00265	1.8E-05	17	89714	ukb-d-22	|| id:u	TRUE	reported	igd	2	TRUE
rs773097	A	C	A	C	0.02921	0.1239	0.01555	0.01453	FALSE	FALSE	FALSE	ieu-a-32	7	73663178	0.0911	27432	0.1738	Ulcerati	Ulcerati	Ulcerati	TRUE	igd	73663178	0.00712	4.1E-05	7	89714	ukb-d-22	|| id:u	TRUE	reported	igd	2	TRUE
rs778055	T	G	T	G	0.01932	-0.0053	0.05764	0.06127	FALSE	FALSE	FALSE	ieu-a-32	6	144834312	0.0469	27432	0.9093	Ulcerati	Ulcerati	Ulcerati	TRUE	igd	144834312	0.0038	3.6E-07	6	89714	ukb-d-22	|| id:u	TRUE	reported	igd	2	TRUE
rs794986	G	A	G	A	-0.0277	-0.2277	0.02296	0.01798	FALSE	FALSE	FALSE	ieu-a-32	8	134855286	0.099	27432	0.02148	Ulcerati	Ulcerati	Ulcerati	TRUE	igd	134855286	0.00583	2E-06	8	89714	ukb-d-22	|| id:u	TRUE	reported	igd	2	TRUE
rs795195	C	T	C	T	-0.0218	0.0229	0.02895	0.023	FALSE	FALSE	FALSE	ieu-a-32	14	34720312	0.0794	27432	0.7728	Ulcerati	Ulcerati	Ulcerati	TRUE	igd	34720312	0.00521	2.9E-05	14	89714	ukb-d-22	|| id:u	TRUE	reported	igd	2	TRUE
rs797808	C	T	C	T	0.02474	-0.1345	0.02269	0.02224	FALSE	FALSE	FALSE	ieu-a-32	7	104247958	0.0753	27432	0.07396	Ulcerati	Ulcerati	Ulcerati	TRUE	igd	104247958	0.00593	3E-05	7	89714	ukb-d-22	|| id:u	TRUE	reported	igd	2	TRUE
rs800655	C	G	C	G	0.01243	0.0081	0.15964	0.17925	FALSE	TRUE	FALSE	ieu-a-32	14	67294069	0.0282	27432	0.7734	Ulcerati	Ulcerati	Ulcerati	TRUE	igd	67294069	0.00244	3.7E-07	14	89714	ukb-d-22	|| id:u	TRUE	reported	igd	2	TRUE
rs876462	G	T	G	T	-0.008	-0.0615	0.37434	0.36346	FALSE	FALSE	FALSE	ieu-a-32	2	37515880	0.0223	27432	0.00591	Ulcerati	Ulcerati	Ulcerati	TRUE	igd	37515880	0.00181	1E-05	2	89714	ukb-d-22	|| id:u	TRUE	reported	igd	2	TRUE
rs884258	C	T	C	T	-0.0079	0.0106	0.67693	0.67327	FALSE	FALSE	FALSE	ieu-a-32	9	107057177	0.0227	27432	0.6423	Ulcerati	Ulcerati	Ulcerati	TRUE	igd	107057177	0.00189	3.3E-05	9	89714	ukb-d-22	|| id:u	TRUE	reported	igd	2	TRUE
rs914186	A	G	A	G	0.00773	0.0002	0.66006	0.671	FALSE	FALSE	FALSE	ieu-a-32	21	42619657	0.0225	27432	0.9934	Ulcerati	Ulcerati	Ulcerati	TRUE	igd	42619657	0.00186	3.3E-05	21	89714	ukb-d-22	|| id:u	TRUE	reported	igd	2	TRUE
rs936835	T	A	T	A	0.03017	0.2423	0.01474	0.02352	FALSE	TRUE	FALSE	ieu-a-32	6	21808192	0.0811	27432	0.00282	Ulcerati	Ulcerati	Ulcerati	TRUE	igd	21808192	0.00733	3.9E-05	6	89714	ukb-d-22	|| id:u	TRUE	reported	igd	2	TRUE
rs953033	G	T	G	T	0.0077	-0.0315	0.65454	0.63975	FALSE	FALSE	FALSE	ieu-a-32	13	75070201	0.022	27432	0.1525	Ulcerati	Ulcerati	Ulcerati	TRUE	igd	75070201	0.00185	3.1E-05	13	89714	ukb-d-22	|| id:u	TRUE	reported	igd	2	TRUE

Table S9. Detail Information of the Genetic Variants in the MR Analysis of SHS and CD

SNP	effect_a	other_al	effect_a	other_al	beta.exp	beta.out	eaf.expo	eaf.outc	remove	palindro	ambiguou	id.outco	chr	pos	se.outco	samplesi	pval.out	outcome	original	outcome.	mr_keep.	data_sou	pos.expos	se.expos	pval.exp	chr.expo	samplesi	id.expos	exposure	mr_keep.	pval_ori	data_sou	action	mr_keep
rs100090	A	G	A	G	-0.0076	0.0118	0.53599	0.532	FALSE	FALSE	FALSE	ieu-a-30	4	180336832	0.0232	20883	0.6112	Crohn's	Crohn's	Crohn's	TRUE	igd	180336832	0.00176	1.4E-05	4	89714	ukb-d-22	|| id:u	TRUE	reported	igd	2	TRUE
rs102193	T	C	T	C	-0.0095	-0.0022	0.1902	0.19672	FALSE	FALSE	FALSE	ieu-a-30	11	86339724	0.0286	20883	0.9381	Crohn's	Crohn's	Crohn's	TRUE	igd	86339724	0.00224	2.4E-05	11	89714	ukb-d-22	|| id:u	TRUE	reported	igd	2	TRUE
rs104255	C	T	C	T	-0.0084	-0.0029	0.70783	0.69272	FALSE	FALSE	FALSE	ieu-a-30	19	836043	0.0353	20883	0.9341	Crohn's	Crohn's	Crohn's	TRUE	igd	836043	0.00204	3.6E-05	19	89714	ukb-d-22	|| id:u	TRUE	reported	igd	2	TRUE
rs109451	A	T	A	T	-0.0079	0.023804	0.31369	0.30057	FALSE	TRUE	FALSE	ieu-a-30	6	69354524	0.0252	20883	0.3449	Crohn's	Crohn's	Crohn's	TRUE	igd	69354524	0.00189	3.2E-05	6	89714	ukb-d-22	|| id:u	TRUE	reported	igd	2	TRUE
rs112169	A	G	A	G	0.03019	0.063998	0.01694	0.00972	FALSE	FALSE	FALSE	ieu-a-30	7	70641982	0.1394	20883	0.646	Crohn's	Crohn's	Crohn's	TRUE	igd	70641982	0.00676	8.1E-06	7	89714	ukb-d-22	|| id:u	TRUE	reported	igd	2	TRUE
rs114134	T	C	T	C	0.04033	0.097	0.00945	0.01089	FALSE	FALSE	FALSE	ieu-a-30	6	41851676	0.1543	20883	0.5296	Crohn's	Crohn's	Crohn's	TRUE	igd	41851676	0.00973	3.4E-05	6	89714	ukb-d-22	|| id:u	TRUE	reported	igd	2	TRUE
rs116249	T	C	T	C	-0.0289	-0.0084	0.01801	0.01926	FALSE	FALSE	FALSE	ieu-a-30	3	190482826	0.1015	20883	0.9339	Crohn's	Crohn's	Crohn's	TRUE	igd	190482826	0.00697	3.5E-05	3	89714	ukb-d-22	|| id:u	TRUE	reported	igd	2	TRUE
rs117410	G	A	G	A	-0.0415	0.129995	0.00888	0.01129	FALSE	FALSE	FALSE	ieu-a-30	15	47979695	0.1135	20883	0.2521	Crohn's	Crohn's	Crohn's	TRUE	igd	47979695	0.00945	1.1E-05	15	89714	ukb-d-22	|| id:u	TRUE	reported	igd	2	TRUE
rs117514	T	G	T	G	0.00909	-0.0398	0.18988	0.18372	FALSE	FALSE	FALSE	ieu-a-30	22	49534383	0.0326	20883	0.2216	Crohn's	Crohn's	Crohn's	TRUE	igd	49534383	0.00223	4.5E-05	22	89714	ukb-d-22	|| id:u	TRUE	reported	igd	2	TRUE
rs117858	A	G	A	G	-0.0212	0.085499	0.03627	0.04106	FALSE	FALSE	FALSE	ieu-a-30	6	149289638	0.0642	20883	0.1827	Crohn's	Crohn's	Crohn's	TRUE	igd	149289638	0.00484	1.2E-05	6	89714	ukb-d-22	|| id:u	TRUE	reported	igd	2	TRUE
rs118098	T	C	T	C	0.02983	0.148299	0.02017	0.01533	FALSE	FALSE	FALSE	ieu-a-30	21	33944718	0.114	20883	0.193	Crohn's	Crohn's	Crohn's	TRUE	igd	33944718	0.00656	5.4E-06	21	89714	ukb-d-22	|| id:u	TRUE	reported	igd	2	TRUE
rs118146	G	A	G	A	-0.0131	-0.0843	0.09386	0.08214	FALSE	FALSE	FALSE	ieu-a-30	8	22116219	0.0514	20883	0.1015	Crohn's	Crohn's	Crohn's	TRUE	igd	22116219	0.00312	2.5E-05	8	89714	ukb-d-22	|| id:u	TRUE	reported	igd	2	TRUE
rs120418	T	C	T	C	0.01394	-0.0279	0.08023	0.08057	FALSE	FALSE	FALSE	ieu-a-30	1	60484531	0.043	20883	0.5161	Crohn's	Crohn's	Crohn's	TRUE	igd	60484531	0.00324	1.7E-05	1	89714	ukb-d-22	|| id:u	TRUE	reported	igd	2	TRUE
rs121057	G	T	G	T	0.00874	0.008304	0.36894	0.38543	FALSE	FALSE	FALSE	ieu-a-30	2	60736985	0.0245	20883	0.7347	Crohn's	Crohn's	Crohn's	TRUE	igd	60736985	0.00184	2E-06	2	89714	ukb-d-22	|| id:u	TRUE	reported	igd	2	TRUE
rs124091	C	T	C	T	-0.0085	0.011	0.24869	0.26072	FALSE	FALSE	FALSE	ieu-a-30	1	114055488	0.0273	20883	0.6864	Crohn's	Crohn's	Crohn's	TRUE	igd	114055488	0.00208	3.9E-05	1	89714	ukb-d-22	|| id:u	TRUE	reported	igd	2	TRUE
rs124840	T	C	T	C	0.00905	-0.0424	0.20587	0.18943	FALSE	FALSE	FALSE	ieu-a-30	22	51099862	0.0301	20883	0.1585	Crohn's	Crohn's	Crohn's	TRUE	igd	51099862	0.00217	3E-05	22	89714	ukb-d-22	|| id:u	TRUE	reported	igd	2	TRUE
rs124932	G	T	G	T	0.01626	0.015205	0.05371	0.05629	FALSE	FALSE	FALSE	ieu-a-30	3	16679114	0.053	20883	0.7736	Crohn's	Crohn's	Crohn's	TRUE	igd	16679114	0.00388	2.8E-05	3	89714	ukb-d-22	|| id:u	TRUE	reported	igd	2	TRUE
rs126292	A	T	A	T	-0.0081	0.027304	0.33223	0.32671	FALSE	TRUE	FALSE	ieu-a-30	6	16806082	0.0255	20883	0.2834	Crohn's	Crohn's	Crohn's	TRUE	igd	16806082	0.00189	1.7E-05	6	89714	ukb-d-22	|| id:u	TRUE	reported	igd	2	TRUE
rs127653	T	C	T	C	0.00867	0.0004	0.23241	0.231	FALSE	FALSE	FALSE	ieu-a-30	10	7631014	0.0272	20883	0.9883	Crohn's	Crohn's	Crohn's	TRUE	igd	7631014	0.0021	3.7E-05	10	89714	ukb-d-22	|| id:u	TRUE	reported	igd	2	TRUE
rs128669	A	G	A	G	0.02524	0.057505	0.02351	0.02291	FALSE	FALSE	FALSE	ieu-a-30	13	110066103	0.0755	20883	0.4462	Crohn's	Crohn's	Crohn's	TRUE	igd	110066103	0.00579	1.3E-05	13	89714	ukb-d-22	|| id:u	TRUE	reported	igd	2	TRUE
rs138858	A	G	A	G	0.04308	0.080796	0.00736	0.01116	FALSE	FALSE	FALSE	ieu-a-30	15	79099986	0.1235	20883	0.5127	Crohn's	Crohn's	Crohn's	TRUE	igd	79099986	0.01047	3.9E-05	15	89714	ukb-d-22	|| id:u	TRUE	reported	igd	2	TRUE
rs140647	G	A	G	A	0.04379	0.187101	0.0076	0.00729	FALSE	FALSE	FALSE	ieu-a-30	19	20290042	0.1531	20883	0.2216	Crohn's	Crohn's	Crohn's	TRUE	igd	20290042	0.01059	3.6E-05	19	89714	ukb-d-22	|| id:u	TRUE	reported	igd	2	TRUE
rs142563	A	G	A	G	-0.0344	-0.0921	0.01473	0.0187	FALSE	FALSE	FALSE	ieu-a-30	13	23278334	0.0979	20883	0.3469	Crohn's	Crohn's	Crohn's	TRUE	igd	23278334	0.00745	4E-06	13	89714	ukb-d-22	|| id:u	TRUE	reported	igd	2	TRUE
rs143255	C	T	C	T	0.01694	-0.0327	0.04814	0.053	FALSE	FALSE	FALSE	ieu-a-30	7	147431844	0.054	20883	0.5446	Crohn's	Crohn's	Crohn's	TRUE	igd	147431844	0.00413	4.1E-05	7	89714	ukb-d-22	|| id:u	TRUE	reported	igd	2	TRUE
rs144479	A	G	A	G	0.05832	-0.1032	0.00476	0.00927	FALSE	FALSE	FALSE	ieu-a-30	3	122728248	0.1378	20883	0.4537	Crohn's	Crohn's	Crohn's	TRUE	igd	122728248	0.01297	6.9E-06	3	89714	ukb-d-22	|| id:u	TRUE	reported	igd	2	TRUE
rs145817	T	C	T	C	0.00807	0.008405	0.54956	0.57028	FALSE	FALSE	FALSE	ieu-a-30	12	41777144	0.023	20883	0.7152	Crohn's	Crohn's	Crohn's	TRUE	igd	41777144	0.00176	4.4E-06	12	89714	ukb-d-22	|| id:u	TRUE	reported	igd	2	TRUE
rs147567	A	G	A	G	0.01947	-0.0423	0.03983	0.0414	FALSE	FALSE	FALSE	ieu-a-30	7	47544762	0.0686	20883	0.5376	Crohn's	Crohn's	Crohn's	TRUE	igd	47544762	0.0047	3.5E-05	7	89714	ukb-d-22	|| id:u	TRUE	reported	igd	2	TRUE
rs148904	C	G	C	G	0.03265	0.002198	0.01511	0.01769	FALSE	TRUE	FALSE	ieu-a-30	22	25249949	0.1082	20883	0.9841	Crohn's	Crohn's	Crohn's	TRUE	igd	25249949	0.0076	1.7E-05	22	89714	ukb-d-22	|| id:u	TRUE	reported	igd	2	TRUE
rs149651	G	C	G	C	0.03813	0.0494	0.01176	0.01172	FALSE	TRUE	FALSE	ieu-a-30	6	160595479	0.1191	20883	0.678	Crohn's	Crohn's	Crohn's	TRUE	igd	160595479	0.00836	5E-06	6	89714	ukb-d-22	|| id:u	TRUE	reported	igd	2	TRUE
rs149772	T	A	T	A	0.0282	-0.0224	0.02041	0.02029	FALSE	TRUE	FALSE	ieu-a-30	4	12576228	0.0997	20883	0.8219	Crohn's	Crohn's	Crohn's	TRUE	igd	12576228	0.00662	2E-05	4	89714	ukb-d-22	|| id:u	TRUE	reported	igd	2	TRUE
rs150100	C	T	C	T	0.02882	-0.0225	0.02023	0.02272	FALSE	FALSE	FALSE	ieu-a-30	3	96018959	0.0765	20883	0.7691	Crohn's	Crohn's	Crohn's	TRUE	igd	96018959	0.00671	1.8E-05	3	89714	ukb-d-22	|| id:u	TRUE	reported	igd	2	TRUE
rs150689	A	G	A	G	0.03224	-0.1099	0.0145	0.01496	FALSE	FALSE	FALSE	ieu-a-30	13	23446586	0.1229	20883	0.3711	Crohn's	Crohn's	Crohn's	TRUE	igd	23446586	0.00765	2.5E-05	13	89714	ukb-d-22	|| id:u	TRUE	reported	igd	2	TRUE
rs171494	G	A	G	A	0.01389	-0.0633	0.08945	0.10486	FALSE	FALSE	FALSE	ieu-a-30	2	235265207	0.0424	20883	0.1356	Crohn's	Crohn's	Crohn's	TRUE	igd	235265207	0.00314	9.5E-06	2	89714	ukb-d-22	|| id:u	TRUE	reported	igd	2	TRUE
rs176026	A	G	A	G	0.03352	-0.1247	0.01586	0.0161	FALSE	FALSE	FALSE	ieu-a-30	12	42480604	0.0989	20883	0.2075	Crohn's	Crohn's	Crohn's	TRUE	igd	42480604	0.00714	2.7E-06	12	89714	ukb-d-22	|| id:u	TRUE	reported	igd	2	TRUE
rs189879	T	G	T	G	0.02926	-0.0903	0.01658	0.00667	FALSE	FALSE	FALSE	ieu-a-30	4	69058096	0.1969	20883	0.6463	Crohn's	Crohn's	Crohn's	TRUE	igd	69058096	0.00705	3.3E-05	4	89714	ukb-d-22	|| id:u	TRUE	reported	igd	2	TRUE
rs192230	A	G	A	G	0.09947	-0.0568	0.00159	0.00756	FALSE	FALSE	FALSE	ieu-a-30	19	50188478	0.2041	20883	0.7808	Crohn's	Crohn's	Crohn's	TRUE	igd	50188478	0.02342	2.2E-05	19	89714	ukb-d-22	|| id:u	TRUE	reported	igd	2	TRUE
rs192463	T	A	T	A	0.02663	0.0044	0.01955	0.013	FALSE	TRUE	FALSE	ieu-a-30	2	213744121	0.1122	20883	0.969	Crohn's	Crohn's	Crohn's	TRUE	igd	213744121	0.00644	3.6E-05	2	89714	ukb-d-22	|| id:u	TRUE	reported	igd	2	TRUE
rs199341	G	C	G	C	0.0083	-0.0012	0.25339	0.27157	FALSE	TRUE	FALSE	ieu-a-30	10	63400853	0.0259	20883	0.9636	Crohn's	Crohn's	Crohn's	TRUE	igd	63400853	0.00202	3.9E-05	10	89714	ukb-d-22	|| id:u	TRUE	reported	igd	2	TRUE
rs203146	C	T	C	T	0.00758	0.011203	0.62391	0.619	FALSE	FALSE	FALSE	ieu-a-30	10	131923448	0.0246	20883	0.6494	Crohn's	Crohn's	Crohn's	TRUE	igd	131923448	0.00181	2.9E-05	10	89714	ukb-d-22	|| id:u	TRUE	reported	igd	2	TRUE
rs206940	G	A	G	A	-0.0084	0.001401	0.34093	0.328	FALSE	FALSE	FALSE	ieu-a-30	12	56364321	0.0242	20883	0.9536	Crohn's	Crohn's	Crohn's	TRUE	igd	56364321	0.00185	5.7E-06	12	89714	ukb-d-22	|| id:u	TRUE	reported	igd	2	TRUE
rs207008	G	T	G	T	-0.0196	0.017095	0.94188	0.93719	FALSE	FALSE	FALSE	ieu-a-30	21	18735936	0.0506	20883	0.7351	Crohn's	Crohn's	Crohn's	TRUE	igd	18735936	0.00375	1.8E-07	21	89714	ukb-d-22	|| id:u	TRUE	reported	igd	2	TRUE
rs229649	G	A	G	A	-0.0113	0.030098	0.11402	0.12143	FALSE	FALSE	FALSE	ieu-a-30	13	95096111	0.0351	20883	0.3906	Crohn's	Crohn's	Crohn's	TRUE	igd	95096111	0.00277	4.3E-05	13	89714	ukb-d-22	|| id:u	TRUE	reported	igd	2	TRUE
rs268312	C	A	C	A	-0.0072	-0.0129	0.46584	0.46443	FALSE	FALSE	FALSE	ieu-a-30	15	36024804	0.0242	20883	0.5928	Crohn's	Crohn's	Crohn's	TRUE	igd	36024804	0.00177	4.9E-05	15	89714	ukb-d-22	|| id:u	TRUE	reported	igd	2	TRUE
rs276583	A	G	A	G	0.01277	0.042504	0.12036	0.12214	FALSE	FALSE	FALSE	ieu-a-30	10	21034317	0.037	20883	0.2505	Crohn's	Crohn's	Crohn's	TRUE	igd	21034317	0.00272	2.7E-06	10	89714	ukb-d-22	|| id:u	TRUE	reported	igd	2	TRUE
rs284717	T	C	T	C	0.00882	-0.0032	0.63416	0.64986	FALSE	FALSE	FALSE	ieu-a-30	11	105877624	0.024	20883	0.8934	Crohn's	Crohn's	Crohn's	TRUE	igd	105877624	0.00183	1.4E-06	11	89714	ukb-d-22	|| id:u	TRUE	reported	igd	2	TRUE
rs309354	A	G	A	G	-0.0185	-0.1019	0.04632	0.05486	FALSE	FALSE	FALSE	ieu-a-30	6	31539010	0.0519	20883	0.04943	Crohn's	Crohn's	Crohn's	TRUE	igd	31539010	0.00418	9.8E-06	6	89714	ukb-d-22	|| id:u	TRUE	reported	igd	2	TRUE
rs310653	A	C	A	C	0.01211	-0.06481	0.10649	0.10049	FALSE	FALSE	FALSE	ieu-a-30	8	131259140	0.0423	20883	0.1257	Crohn's	Crohn's	Crohn's	TRUE	igd	131259140	0.00284	2E-05	8	89714	ukb-d-22	|| id:u	TRUE	reported	igd	2	TRUE
rs353857	T	A	T	A	0.00794	-0.0312	0.63465	0.62443	FALSE	TRUE	FALSE	ieu-a-30	3	196071832	0.0242	20883	0.196	Crohn's	Crohn's	Crohn's	TRUE	igd	196071832	0.00183	1.4E-05	3	89714	ukb-d-22	|| id:u	TRUE	reported	igd	2	TRUE
rs373969	A	G	A	G	0.00727	-0.0684	0.49641	0.48758	FALSE	FALSE	FALSE	ieu-a-30	9	114798940	0.0259	20883	0.00837	Crohn's	Crohn's	Crohn's	TRUE	igd	114798940	0.00177	3.9E-05	9	89714	ukb-d-22	|| id:u	TRUE	reported	igd	2	TRUE
rs381770	G	C	G	C	-0.0072	-0.0146	0.57626	0.58143	FALSE	TRUE	TRUE	ieu-a-30	8	2054195	0.0231	20883	0.5266	Crohn's	Crohn's	Crohn's	TRUE	igd	2054195	0.00178	4.9E-05	8	89714	ukb-d-22	|| id:u	TRUE	reported	igd	2	FALSE
rs382594	A	G	A	G	0.00829	0.002397	0.25777	0.25257	FALSE	FALSE	FALSE	ieu-a-30	15	74317362	0.0264	20883	0.9283	Crohn's	Crohn's	Crohn's	TRUE	igd	74317362	0.00202	3.9E-05	15	89714	ukb-d-22	|| id:u	TRUE	reported	igd	2	TRUE
rs389792	T	G	T	G	-0.0075	0.003703	0.431	0.45643	FALSE	FALSE	FALSE	ieu-a-30	3	101703056	0.0231	20883	0.8729	Crohn's	Crohn's	Crohn's	TRUE	igd	101703056	0.00177	2.1E-05	3	89714	ukb-d-22	|| id:u	TRUE	reported	igd	2	TRUE
rs413194	T	G	T	G	-0.0074	0.007105	0.51371	0.50143	FALSE	FALSE	FALSE	ieu-a-30	16	54862861	0.0234	20883	0.7624	Crohn's	Crohn's	Crohn's	TRUE	igd	54862861	0.00178	3.1E-05	16	89714	ukb-d-22	|| id:u	TRUE	reported	igd	2	TRUE
rs443395	T	A	T	A	0.0078	-0.0258	0.43142	0.42629	FALSE	TRUE	TRUE	ieu-a-30	2	238115406	0.024	20883	0.281	Crohn's	Crohn's	Crohn's	TRUE	igd	238114802	0.00182	1.7E-05	2	89714	ukb-d-22	|| id:u	TRUE	reported	igd	2	FALSE
rs468829	T	C	T	C	0.01399	-0.0436	0.07414	0.07596	FALSE	FALSE	FALSE	ieu-a-30	3	62460883	0.045	20883	0.3323	Crohn's	Crohn's	Crohn's	TRUE	igd	62460883	0.00341	4E-05	3	89714	ukb-d-22	|| id:u	TRUE	reported	igd	2	TRUE
rs481445	C	T	C	T	-0.0168	0.061397	0.94596	0.94163	FALSE	FALSE	FALSE	ieu-a-30	20	16178198	0.0493	20883	0.2125	Crohn's	Crohn's	Crohn's	TRUE	igd	16178198	0.00387	1.5E-05	20	89714	ukb-d-22	|| id:u	TRUE	reported	igd	2	TRUE
rs497501	A	G	A	G	0.00903	0.002896	0.22088	0.222	FALSE	FALSE	FALSE	ieu-a-30	4	39423512	0.029	20883	0.9201	Crohn's	Crohn's	Crohn's	TRUE	igd	39423512	0.00213	2.2E-05	4	89714	ukb-d-22	|| id:u	TRUE	reported	igd	2	TRUE
rs513564	T	C	T	C	0.00846	0.088203	0.24995	0.23828	FALSE	FALSE	FALSE	ieu-a-30	10	99780908	0.0285	20883	0.00194	Crohn's	Crohn's	Crohn's	TRUE	igd	99780908	0.00207	4.4E-05	10	89714	ukb-d-22	|| id:u	TRUE	reported	igd	2	TRUE
rs554912	C	G	C	G	-0.0124	-0.08379	0.10323	0.09998	FALSE	TRUE	FALSE	ieu-a-30	1	226737915	0.0398	20883	0.03505	Crohn's	Crohn's	Crohn's	TRUE	igd	226735908	0.00295	2.8E-05	1	89714	ukb-d-22	|| id:u	TRUE	reported	igd	2	TRUE
rs558136	T	C	T	C	0.03104	-0.1908	0.01488	0.01754	FALSE	FALSE	FALSE	ieu-a-30	8	62318022	0.0934	20883	0.04092	Crohn's	Crohn's	Crohn's	TRUE	igd	62318022	0.00726	1.9E-05	8	89714	ukb-d-22	|| id:u	TRUE	reported	igd	2	TRUE
rs559280	A	G	A	G	-0.026	-0.0941	0.02501	0.02146	FALSE	FALSE	FALSE	ieu-a-30	16	69956730	0.0876	20883	0.2827	Crohn's	Crohn's	Crohn's	TRUE	igd	69750910	0.00566	4.2E-06	16	89714	ukb-d-22	|| id:u	TRUE	reported	igd	2	TRUE
rs561694	T	C	T	C	-0.0141	0.014997	0.07356	0.06577	FALSE	FALSE	FALSE	ieu-a-30	12	117155472	0.0559	20883	0.7886	Crohn's	Crohn's	Crohn's	TRUE	igd	117155472	0.00347	4.9E-05	12	89714	ukb-d-22	|| id:u	TRUE	reported	igd	2	TRUE
rs576053	C	G	C	G	-0.0218	-0.1241	0.02771	0.03514	FALSE	TRUE	FALSE	ieu-a-30	6	151835770	0.0694	20883	0.0737	Crohn's	Crohn's	Crohn's	TRUE	igd	151835770	0.00534	4.6E-05	6	89714	ukb-d-22	|| id:u	TRUE	reported	igd	2	TRUE
rs611254	T	C	T	C	-0.0151	-0.0521	0.08847	0.08639	FALSE	FALSE	FALSE	ieu-a-30	20	19727485	0.0413	20883	0.2065	Crohn's	Crohn's	Crohn's	TRUE	igd	19727485	0.00308	8.6E-07	20	89714	ukb-d-22	|| id:u	TRUE	reported	igd	2	TRUE
rs613060	C	G	C	G	-0.0103	-0.035	0.19003	0.18957	FALSE	TRUE	FALSE	ieu-a-30	20	43033095	0.0319	20883	0.2717	Crohn's	Crohn's	Crohn's	TRUE	igd	43033095	0.00223	3.8E-06	20	89714	ukb-d-22	|| id:u	TRUE	reported	igd	2	TRUE
rs620553	G	T	G	T	-0.014	0.0455	0.08724	0.073	FALSE	FALSE	FALSE	ieu-a-30	16	30187673	0.0526	20883	0.387	Crohn's	Crohn's	Crohn's	TRUE	igd	30187673	0.00321	1.4E-05	16	89714	ukb-d-22	|| id:u	TRUE	reported	igd	2	TRUE
rs622080	G	A	G	A	0.02549	0.066995	0.02231	0.02172	FALSE	FALSE	FALSE	ieu-a-30	20	62043227	0.0939	20883	0.4758	Crohn's	Crohn's	Crohn's	TRUE	igd	62043227	0.00592	1.6E-05	20	89714	ukb-d-22	|| id:u	TRUE	reported	igd	2	TRUE
rs624773	T	A	T	A	0.02016	-0.0381	0.04486	0.03672	FALSE	TRUE	FALSE	ieu-a-30	7	114935274	0.0653	20883	0.5596	Crohn's	Crohn's	Crohn's	TRUE	igd	114935274	0.00426	2.2E-06	7	89714	ukb-d-22	|| id:u	TRUE	reported	igd	2	TRUE
rs624961	T	C	T	C	0.02838	-0.0533	0.02176	0.01887	FALSE	FALSE	FALSE	ieu-a-30	8	9288470	0.1059	20883	0.6147	Crohn's	Crohn's	Crohn's	TRUE	igd	9288470	0.00632	7.2E-06	8	89714	ukb-d-22	|| id:u	TRUE	reported	igd	2	TRUE
rs688129	C	T	C	T	0.00745	0.044495	0.58387	0.58214	FALSE	FALSE	FALSE	ieu-a-30	5	11147494	0.023	20883	0.05342	Crohn's	Crohn's	Crohn's	TRUE	igd	11147494	0.00177	2.7E-05	5	89714	ukb-d-22	|| id:u	TRUE	reported	igd	2	TRUE
rs689896	G	C	G	C	0.00762	0.008002	0.63769	0.61914	FALSE	TRUE	FALSE	ieu-a-30	18	74078911	0.0258	20883	0.7576	Crohn's	Crohn's	Crohn's	TRUE	igd	74078911	0.00182	2.8E-05	18	89714	ukb-d-22	|| id:u	TRUE	reported	igd	2	TRUE
rs691490	C	A	C	A	0.01026	0.003998	0.14903	0.139	FALSE	FALSE	FALSE	ieu-a-30	6	139021524	0.0342	20883	0.908	Crohn's	Crohn's	Crohn's	TRUE	igd	139021524	0.00249	3.7E-05	6	89714	ukb-d-22	|| id:u	TRUE	reported	igd	2	TRUE
rs708246	A	G	A	G	-0.0085	-0.028	0.67891	0.67243	FALSE	FALSE	FALSE	ieu-a-30	10	50486727	0.0248	20883	0.2603	Crohn's	Crohn's	Crohn's	TRUE	igd	50486727	0.00188	6.2E-06	10	89714	ukb-d-22	|| id:u	TRUE	reported	igd	2	TRUE
rs720454	G	C	G	C	0.00922	-0.0312	0.69183	0.69414	FALSE	TRUE	FALSE	ieu-a-30	16	718514	0.0274	20883	0.255	Crohn's	Crohn's	Crohn's	TRUE	igd	718514	0.00197	2.8E-06	16	89714	ukb-d-22	|| id:u	TRUE	reported	igd	2	TRUE
rs726791	A	G	A	G	0.01238	-0.0374	0.09252	0.08719	FALSE	FALSE	FALSE	ieu-a-30	1	66625369	0.0412	20883	0.3645	Crohn's	Crohn's	Crohn's	TRUE	igd	66625369	0.00303	4.5E-05	1	89714	ukb-d-22	|| id:u	TRUE	reported	igd	2	TRUE
rs727260	G	C	G	C	-0.0115	-0.0886	0.1251	0.109	FALSE	TRUE	FALSE	ieu-a-30	9	38072104	0.0383	20883	0.02074	Crohn's	Crohn's	Crohn's	TRUE	igd	38072104	0.00267	1.5E-05	9	89714	ukb-d-22	|| id:u	TRUE	reported	igd	2	TRUE
rs727603	C	T	C	T	0.03144	0.059697	0.01409	0.013	FALSE	FALSE	FALSE	ieu-a-30	15	84192374	0.1034	20883	0.5638	Crohn's	Crohn's	Crohn's	TRUE	igd	84192374	0.00758	3.3E-05	15	89714	ukb-d-22	|| id:u	TRUE	reported	igd	2	TRUE
rs728961	A	G	A	G	0.0118	0.0938	0.1081	0.09161	FALSE	FALSE	FALSE	ieu-a-30	11	5845481	0.0481	20883	0.05109	Crohn's	Crohn's	Crohn's	TRUE	igd	5845481	0.00291	5E-05	11	89714	ukb-d-22	|| id:u	TRUE	reported	igd	2	TRUE
rs732141	C	A	C	A	-0.0088	-0.0373	0.24763	0.22729	FALSE	FALSE	FALSE	ieu-a-30	12	121476099	0.0284	20883	0.1902	Crohn's	Crohn's	Crohn's	TRUE	igd	121476099	0.00203	1.7E-05	12	89714	ukb-d-22	|| id:u	TRUE	reported	igd	2	TRUE
rs733601	C	T	C	T	-0.0085	0.017604	0.25051	0.22072	FALSE	FALSE	FALSE	ieu-a-30	21	41403037	0.0288	20883	0.5416	Crohn's	Crohn's	Crohn's	TRUE	igd	41403037	0.00203	3E-05	21	89714	ukb-d-22	|| id:u	TRUE	reported	igd	2	TRUE
rs733619	C	T	C	T	-0.0073	0.024303	0.44239	0.43614	FALSE	FALSE	FALSE	ieu-a-30	13	53911048	0.0237	20883	0.3064	Crohn's	Crohn's	Crohn's	TRUE	igd	53911048	0.00178	4.3E-05	13	89714	ukb-d-22	|| id:u	TRUE	reported	igd	2	TRUE
rs744051	A	G	A	G	-0.0229	-0.0976	0.02571	0.0226	FALSE	FALSE	FALSE	ieu-a-30	2	208711524	0.0899	20883	0.2773	Crohn's	Crohn's	Crohn's	TRUE	igd	208711524	0.00563	5E-05	2	89714	ukb-d-22	|| id:u	TRUE	reported	igd	2	TRUE
rs746397	T	A	T	A	0.01254	-0.0185	0.08923	0.09	FALSE	TRUE	FALSE	ieu-a-30	2	144885367	0.0441	20883	0.6751	Crohn's	Crohn's	Crohn's	TRUE	igd	144885367	0.00307	4.5E-05	2	89714	ukb-d-22	|| id:u	TRUE	reported	igd	2	TRUE
rs762178	G	A	G	A	0.04401	-0.1159	0.00963	0.00872	FALSE	FALSE	FALSE	ieu-a-30	8	94600393	0.156	20883	0.4574	Crohn's	Crohn's	Crohn's	TRUE	igd	94600393	0.00898	9.5E-07	8	89714	ukb-d-22	|| id:u	TRUE	reported	igd	2	TRUE
rs768794	T	C	T	C	-0.0227	0.077803	0.02696	0.01347	FALSE	FALSE	FALSE	ieu-a-30	10	87990995	0.1237	20883	0.5296	Crohn's	Crohn's	Crohn's	TRUE	igd	87990995	0.00541	2.8E-05	10	89714	ukb-d-22	|| id:u	TRUE	reported	igd	2	TRUE
rs772112	A	T	A	T	0.01135	0.0482	0.86874	0.87543	FALSE	TRUE	FALSE	ieu-a-30	17	33621127	0.0416	20883	0.2458	Crohn's	Crohn's	Crohn's	TRUE	igd	33621127	0.00265	1.8E-05	17	89714	ukb-d-22	|| id:u	TRUE	reported	igd	2	TRUE
rs773097	A	C	A	C	0.02921	0.082004	0.01555	0.01474	FALSE	FALSE	FALSE	ieu-a-30	7	73663178	0.0962	20883	0.394	Crohn's	Crohn's	Crohn's	TRUE	igd	73663178	0.00712	4.1E-05	7	89714	ukb-d-22	|| id:u	TRUE	reported	igd	2	TRUE
rs778055	T	G	T	G	0.01932	0.0752	0.05764	0.06157	FALSE	FALSE	FALSE	ieu-a-30	6	144834312	0.0512	20883	0.142	Crohn's	Crohn's	Crohn's	TRUE	igd	144834312	0.0038	3.6E-07	6	89714	ukb-d-22	|| id:u	TRUE	reported	igd	2	TRUE
rs794986	G	A	G	A	-0.0277	0.128299	0.02296	0.01929	FALSE	FALSE	FALSE	ieu-a-30	8	134855286	0.0935	20883	0.1701	Crohn's	Crohn's	Crohn's	TRUE	igd	134855286	0.00583	2E-06	8	89714	ukb-d-22	|| id:u	TRUE	reported	igd	2	TRUE
rs795195	C	T	C	T	-0.0218	0.057396	0.02895	0.02229	FALSE	FALSE	FALSE	ieu-a-30	14	34720312	0.0873	20883	0.5111	Crohn's	Crohn's	Crohn's	TRUE	igd	34720312	0.00521	2.9E-05	14	89714	ukb-d-22	|| id:u	TRUE	reported	igd	2	TRUE
rs797808	C	T	C	T	0.02474	-0.1538	0.02269	0.02214	FALSE	FALSE	FALSE	ieu-a-30	7	104247958	0.0828	20883	0.06312	Crohn's	Crohn's	Crohn's	TRUE	igd	104247958	0.00593	3E-05	7	89714	ukb-d-22	|| id:u	TRUE	reported	igd	2	TRUE
rs800655	C	G	C	G	0.01243	0.036101	0.15964	0.18557	FALSE	TRUE	FALSE	ieu-a-30	14	67294069	0.0311	20883	0.2452	Crohn's	Crohn's	Crohn's	TRUE	igd	67294069	0.00244	3.7E-07	14	89714	ukb-d-22	|| id:u	TRUE	reported	igd	2	TRUE
rs876462	G	T	G	T	-0.008	-0.0091	0.37434	0.36672	FALSE	FALSE	FALSE	ieu-a-30	2	37515880	0.0241	20883	0.7057	Crohn's	Crohn's	Crohn's	TRUE	igd	37515880	0.00181	1E-05	2	89714	ukb-d-22	|| id:u	TRUE	reported	igd	2	TRUE
rs884258	C	T	C	T	-0.0079	0.006199	0.67693	0.67	FALSE	FALSE	FALSE	ieu-a-30	9	107057177	0.0241	20883	0.7971	Crohn's	Crohn's	Crohn's	TRUE	igd	107057177	0.00189	3.3E-05	9	89714	ukb-d-22	|| id:u	TRUE	reported	igd	2	TRUE
rs914186	A	G	A	G	0.00773	0.057703	0.66006	0.67799	FALSE	FALSE	FALSE	ieu-a-30	21	42619657	0.0245	20883	0.01862	Crohn's	Crohn's	Crohn's	TRUE	igd	42619657	0.00186	3.3E-05	21	89714	ukb-d-22	|| id:u	TRUE	reported	igd	2	TRUE
rs936835	T	A	T	A	0.03017	0.086299	0.01474	0.02186	FALSE	TRUE	FALSE	ieu-a-30	6	21808192	0.0937	20883	0.3569	Crohn's	Crohn's	Crohn's	TRUE	igd	21808192	0.00733	3.9E-05	6	89714	ukb-d-22	|| id:u	TRUE	reported	igd	2	TRUE
rs953033	G	T	G	T	0.0077	-0.0178	0.65454	0.63529	FALSE	FALSE	FALSE	ieu-a-30	13	75070201	0.0237	20883	0.4528	Crohn's	Crohn's	Crohn's	TRUE	igd	75070201	0.00185	3.1E-05	13	89714	ukb-d-22	|| id:u	TRUE	reported	igd	2	TRUE

Supplementary Figure 3: MR leave-one-out sensitivity analysis of Second-Hand Smoking on nonmalignant Digestive System Diseases. Circles indicate the results of MR analysis of remaining SNPs on Second-Hand Smoking on nonmalignant Digestive System Diseases after omitting each SNP in turn. Bars indicate CI. (A) gastroesophageal reflux disease; (B) irritable bowel syndrome; (C) cholelithiasis; (D) acute pancreatitis; (E) chronic pancreatitis; (F) inflammatory bowel disease; (G) ulcerative colitis; (H) Crohn's disease.

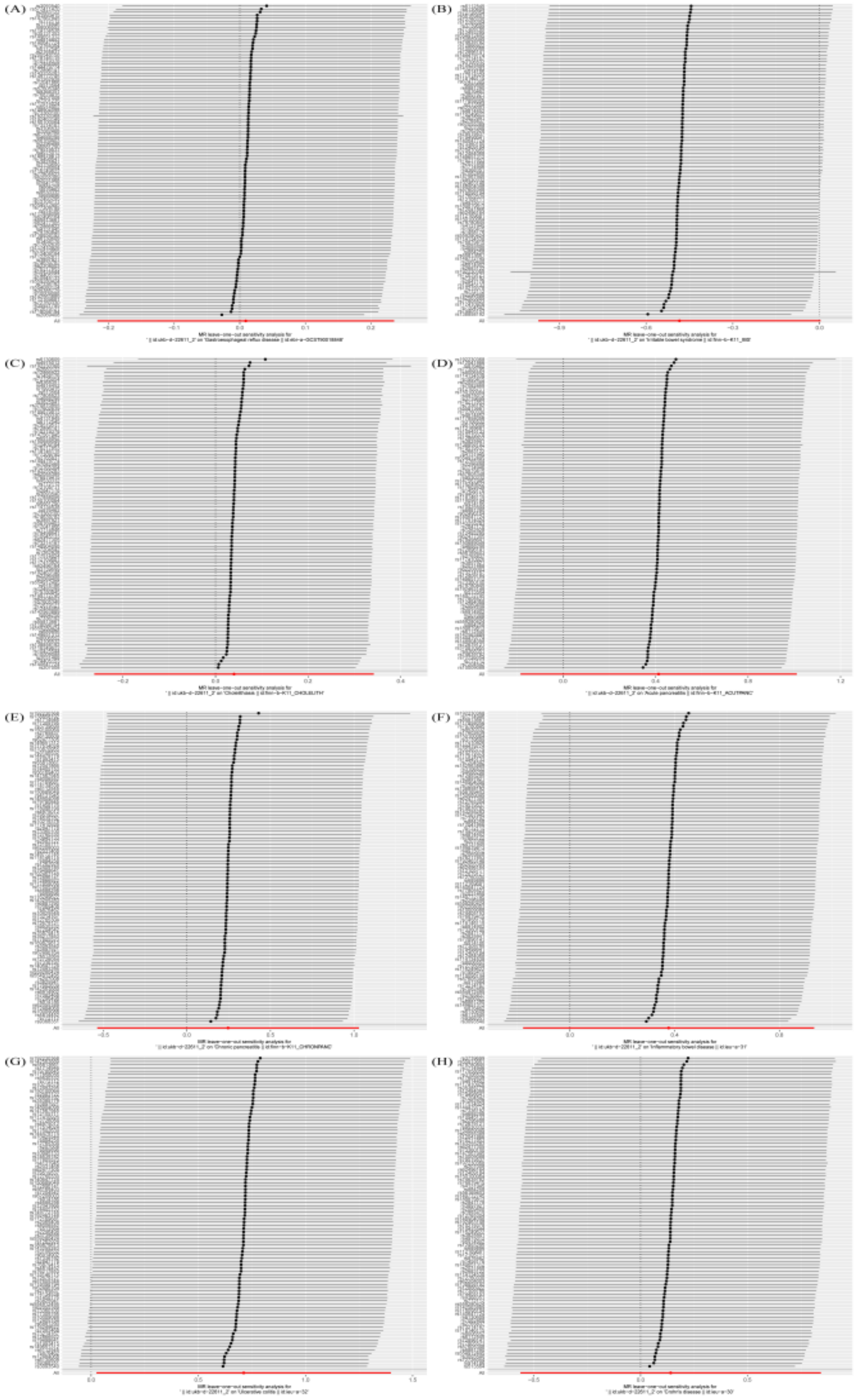

Supplementary Figure 4: Estimating heterogeneity using funnel plots of individual causal relationships between Second-Hand Smoking and nonmalignant Digestive System Diseases. (A) gastroesophageal reflux disease; (B) irritable bowel syndrome; (C) cholelithiasis; (D) acute pancreatitis; (E) chronic pancreatitis; (F) inflammatory bowel disease; (G) ulcerative colitis; (H) Crohn's disease.

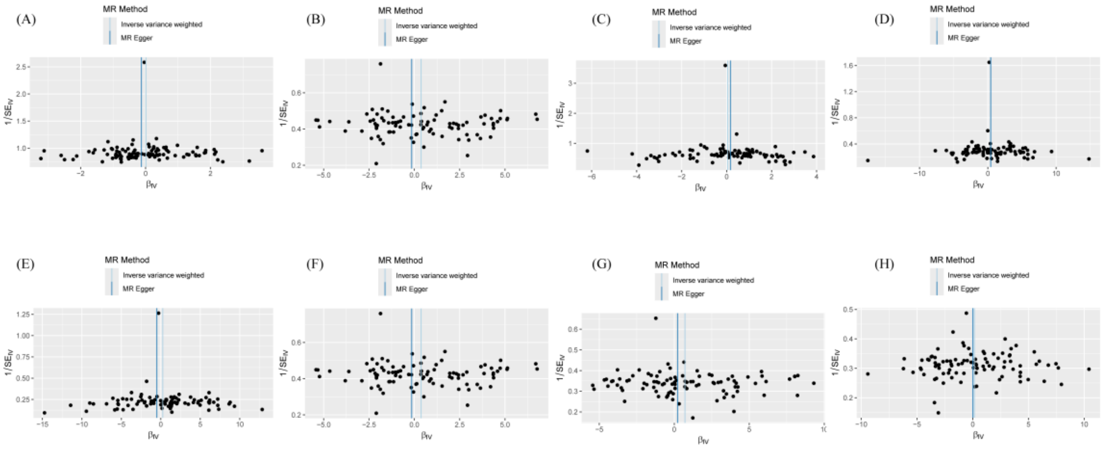

© 2025 Yu Y. and Jin Y.
